# Complex molecular logic gates from simple molecules†

**DOI:** 10.1039/d1ra00930c

**Published:** 2021-06-14

**Authors:** Osvaldo J. Quintana-Romero, Armando Ariza-Castolo

**Affiliations:** Departamento de Química, Centro de Investigación y de Estudios Avanzados del Instituto Politécnico Nacional Av. IPN 2508, San Pedro Zacatenco 07360, Ciudad de México Mexico aariza@cinvestav.mx

## Abstract

Molecular logic gates (MLGs) are compounds that can solve Boolean logic operations to give an answer (OUTPUT) upon receiving a stimulus (INPUT). These derivatives can be used as biological sensors and are promising substitutes for the present logic gates. Although MLGs with complex molecular structures have been reported, they often show stability problems. To address this problem, we describe herein six stable pseudo-hemiindigo-derived MLGs capable of solving complex logic operations. MLGs 7, 8, 9, and 10 can solve a complex logic operation connecting 4 logic gates using 2 different wavelengths (445 nm and 400 nm) and the presence of *p*-TsOH and triethylamine (TEA) as inputs; MLG 11 solves a complex logic operation connecting 3 logic gates and uses 3 inputs, one wavelength of 445 nm and the presence of *p*-TsOH and TEA; and MLG 12 can only solve one logic operation (INH) and uses only the presence of *p*-TsOH and TEA as an input. Each operating method of the MLGs was evaluated with several techniques; proton interactions with MLGs were screened with NMR by titrating with *p*-TsOH, the photochemical properties were examined with absorption ultraviolet-visible (UV-Vis) spectroscopy, and the isomerization dynamics were examined with NMR using the two wavelengths for isomerization (photostationary isomer). The results indicate that the pseudo-hemiindigo-derived MLGs described herein can be applied as multiplexers or data selectors that are necessary for the transient flow of information for biological and computer systems. Finally, to design different MLGs and a system that can treat more information as complex logic gates (demultiplexers), two and three MLGs were mixed in different experiments. In both cases, four inputs were employed (445 nm, 400 nm, *p*-TsOH and TEA), yielding more outputs. Detailed information about the system dynamics was obtained from NMR experiments.

## Abstract 2

Herein we describe a protocol to mimic an electronic device. The MLG could function as a transmitter of information at a molecular level and this could be read using the variation of the magnetic field in the molecules.

## Graphical abstract

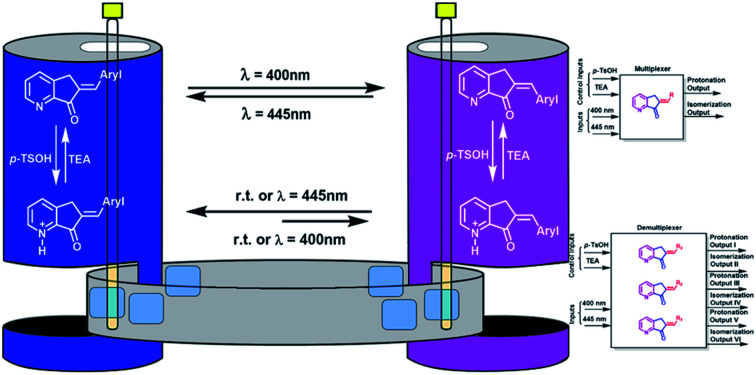

## Introduction

The development of molecular machines is of great interest due to their potential application for solving problems in daily life.^1,2^ This research field, which started as a mental exercise,^1,3,4^ has led to the synthesis of molecules such as catenanes, rotaxanes,^1,3,5^ molecular walkers,^4^ molecular switches^5–9^ and molecular rotors,^5–7,9–12^ which can find applications as smart drugs,^13,14^ materials with memory, organocatalysts^15,16^ and protecting groups, among others.^17,18^

**Fig. 1:**
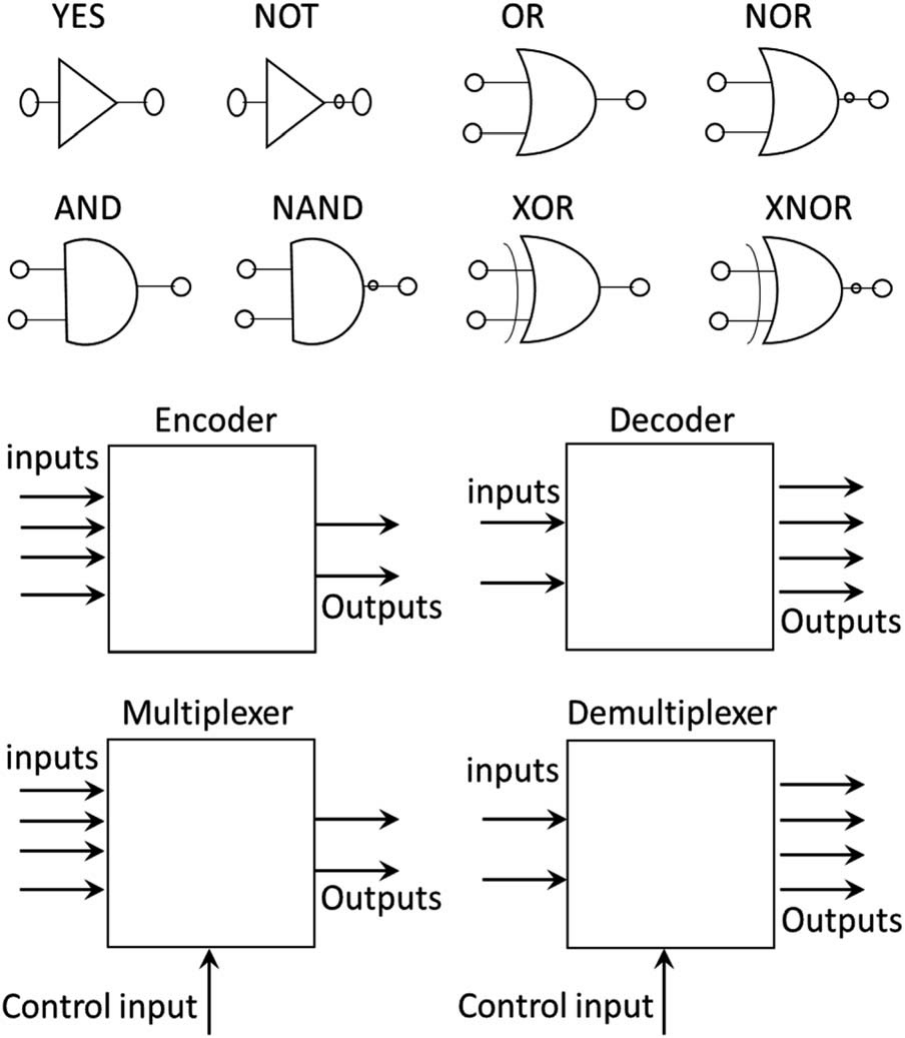
Representation of basic logic gates.

At the same time, molecular machines were developed to introduce molecular-scale logic gates (MLGs). A logic gate is an electronic device that performs a logic operation as required in computers, cellular phones and digital devices; analogously, molecular logic gates are molecules that can perform logic operations.^19–24^ These molecular logic gates were developed to replace electronic circuits with molecules and make smaller devices that can store and handle more information. To date, MLGs that perform basic logical operations, such as AND, OR, NOT, NAND, NOR, XOR and XNOR (Fig. 1), or even more complex logic gates, such as combined circuits that can be constructed with basic logic gates, such as half-adders, half-subtractors, encoders, decoders, multiplexers and demultiplexers, have been reported.^19,22–29^ To expand the interactions of MLGs and their responses in electronic devices, complex MLGs have been developed that can solve various logic operations. For a molecule to be an MLG, based on the definition of a logic gate, it must respond to an external stimulus (input) and provide a response (output).^19–24^

In this work, MLGs are considered molecular machines that can perform complex work, such as solving logic operations, and upon application of an external stimulus (inputs) can be chemical, such as the pH,^7^ REDOX potential,^5^ ionic species, subtracts,^5,22,27^ or polarity of the environment, or physical, such as temperature or light.^20,23,25,26,28,29^ Similarly, the responses (outputs) can be chemical or physical. There are different ways to receive these responses depending on the MLG.^19–24^ Further information about the main concepts has been published in several reviews.^20–22,24,25,30^

**Scheme 1:**
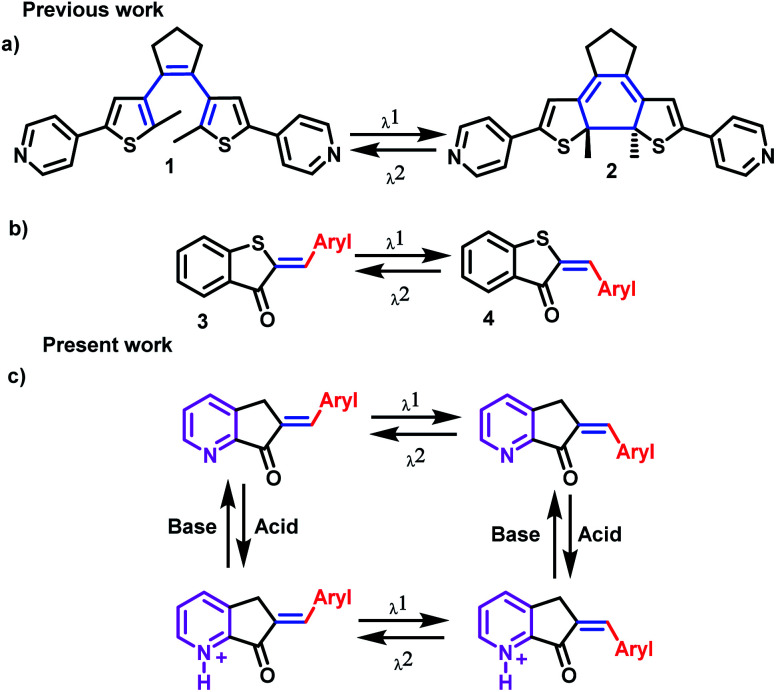
(a) Molecular switch with open-closed structure absorbing at two different wavelengths. (b) Molecular switch, which undergoes *Z*–*E* isomerism at two different wavelengths. (c) Molecular structure of MLGs reported in the present work. The blue mark in the double bond highlights the *E*–*Z* isomerism around the pseudo-hemiindigo base fragment; the nitrogen is responsible for the interaction with protons and is marked in violet.

Molecular switches, which are molecules that can experience chemical changes, such as isomerism responses, are examples of successful types of MLGs. For an MLG to be functional, these isomers must be stable enough to be detected as an output, and with another stimulus, they must be capable of returning to the initial isomer form. The two most common mechanisms by which molecular switches operate are cyclization (open and cyclized isomer) and *Z* and *E* isomerization (Scheme 1a and b).^8,9,25,26,31^

Although several MLGs have been reported to date, they often have complex structures, their preparation is difficult, they are sometimes not stable enough to become an ideal MLG, and demand by the scientific community for new protocols to prepare powerful and small devices is continuously growing. For these reasons, we undertook the task of developing a simple MLG that can perform basic and complex logic operations.^19,20^

In our case, the MLG design is based on a molecular switch that undergoes *Z* and *E* isomerism; for this reason, the axis of the molecular switch must be a double bond. The next condition imposed on our MLG is that one of the inputsbe visible light since it can be readily controlled, although care should be taken to avoid fatiguing the MLG and prevent breakdown or alternative photochemical reactions.^8,11,12,25,26^ In addition, the MLG should be capable of interacting with protons to have another input, in this case, the pyridine fragment,^7,10^ while electron delocalization allows interactions with visible light. With these characteristics, we selected the hemiindigo structure, as it has been shown to be excellent for the design of molecular switches due to its interaction with visible light. In addition, *Z* and *E* pseudo-hemiindigo isomers present characteristic absorption spectra and, consequently, can be used for selective isomerization (Scheme 1c).^32–36^

The outputs of the pseudo-hemiindigo derivatives were determined from their absorption and emission UV-Vis spectra,^19^ in combination with nuclear magnetic resonance (NMR) experiments, which provided important information about the isomer structure and dynamics.^7,37–39^ A problem that was envisioned was the difficulty in analyzing complex mixtures and making unequivocal structural assignments in the reaction medium. In addition to the UV-Vis absorption and emission spectra of the MLG, dynamic studies must be supported by nuclear magnetic resonance (NMR).^7,37–39^ In this work, we focused on NMR spectroscopy to study pseudo-hemiindigo MLGs because a variation in the magnetic field can provide an answer that can be used to store information. Therefore, we created a protocol to mimic an electronic device. The MLG could function as a transmitter of information at a molecular level, which could be read using the variation in the magnetic field in the molecules.^40^

A multiplexer is a device that selects from several input signals and forwards the selected input to a single output. In our case, the inputs are irradiation at 400 nm or 445 nm, the control inputs are *p*-TsOH and TEA, and the outputs are protonation or isomerization; therefore, they function in a mode similar to that of the multiplexer. Conversely, a demultiplexer is a device that takes a single input and select signals of the output. In this case, the inputs and control inputs are similar to those of multiplexer, but the outputs are the selective protonation or isomerization of a mixture of MLGs.

## Results and discussion

### Synthesis of MLGs

**Scheme 2:**
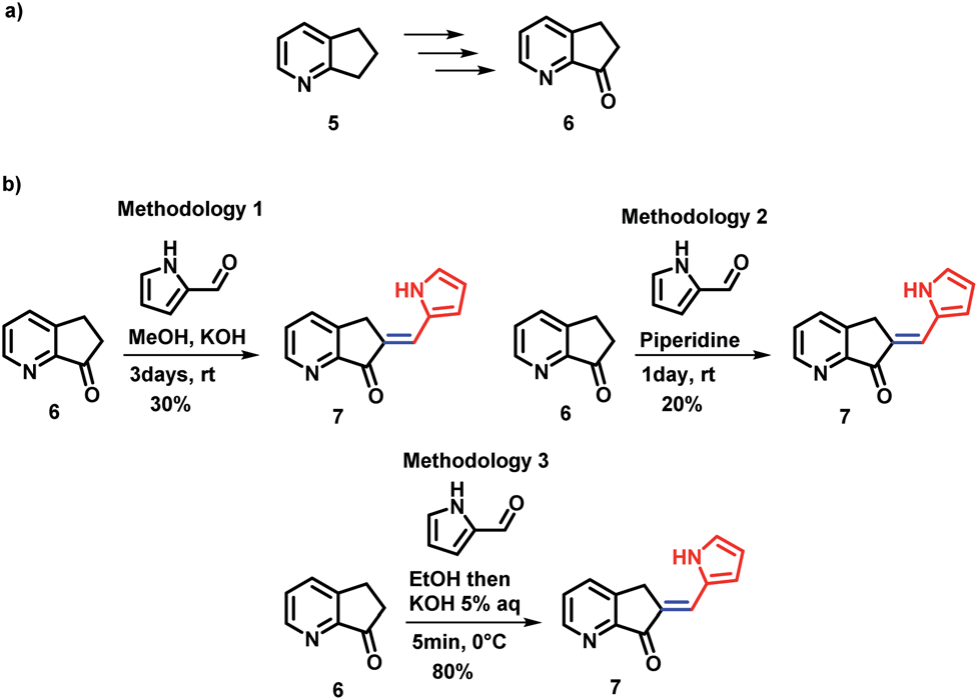
(a) Ketone 6 precursor. (b) Methodologies explored for the synthesis of compound 7.

Several synthetic methods have been explored for the preparation of MLG 7. Block 6 was obtained in four steps in 85% yield from 2,3-cyclopentenopyridine 5 using the protocol reported by Andreotti *et al.*^41^ (Scheme 2a). To optimize the preparation of MLG 7, ketone 6 was reacted with pyrrol-2-carboxaldehyde using three methodologies (Scheme 2 and ESI,† Synthesis section). The best results were obtained when a 5% potassium hydroxide aqueous solution was added dropwise to an EtOH solution of ketone 6 and pyrrol-2-carboxaldehyde at 0 °C until a precipitate was observed, and the solution was frozen (methodology 3). Then, the product was filtered under vacuum to obtain MLG 7 with an 80% yield and 97% purity (Scheme 2b). The same procedure was used to obtain rotors 7 to 12.^42,43^

**Chart 1:**
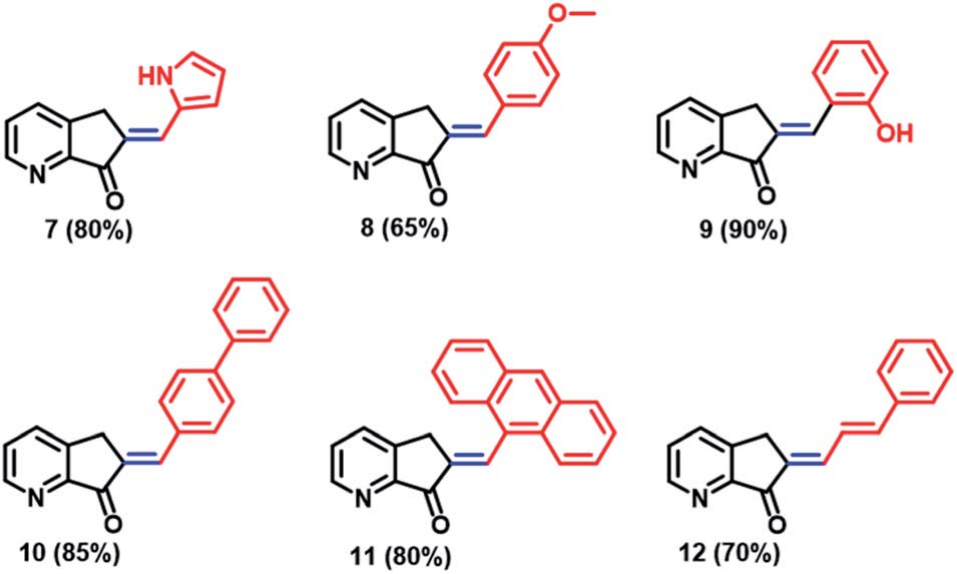
Chemical structure and yields of the MLGs prepared in this work. All compounds were prepared with methodology 3 and identified by ^1^H and ^13^C NMR.

The MLG series prepared, along with the reaction yields, are presented in Chart 1. Starting with the *p*-anisaldehyde derivative, which was obtained in moderate yield (65%), aldol condensation with salicylaldehyde afforded the product with a 90% yield and provided less water during the process of product precipitation. In general, protic solvents were used to increase product solubility. In the case of compounds such as *p*-phenylbenzaldehyde or 9-anthracenecarboxaldehyde, it became necessary to heat the alcohol mixture and add a small amount of dichloromethane as a cosolvent to dissolve the reactants; the products were obtained with 85% and 80% yields. In addition, when the derivative of cinnamaldehyde was prepared with the same methodology, the yield was 70% (Chart 1).

**Fig. 2:**
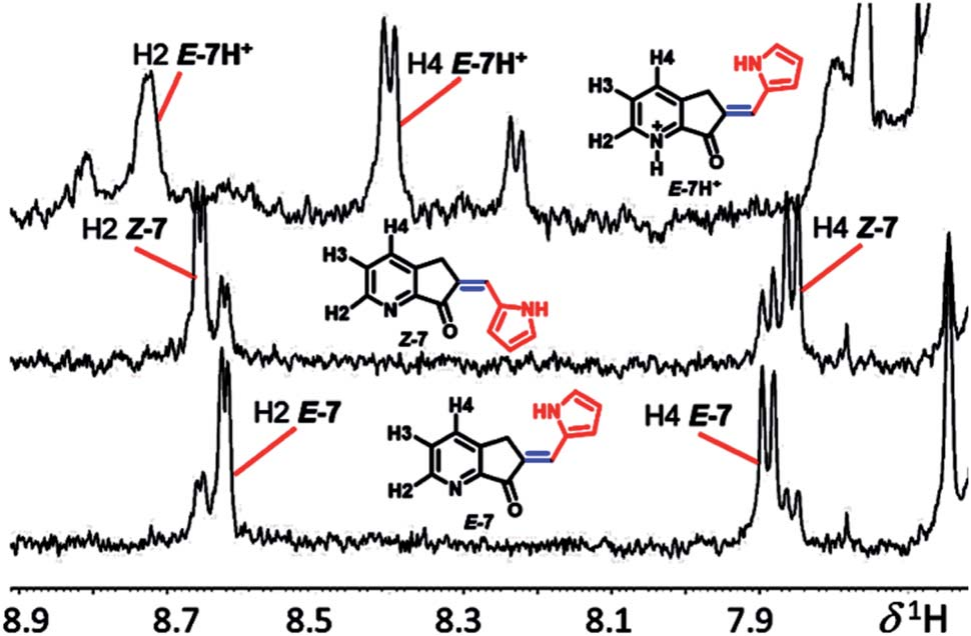
^1^H NMR spectra of compound 7, isomers *E*, *Z* and *E*-7H^+^.

All compounds were characterized by UV-Vis, MS, and ^1^H and ^13^C NMR using 2D HSQC, HMBC, and nuclear Overhauser enhancement. NMR provides the most reliable information in the determination of molecular structure, conformation, and conjugate acid formation in solution; in agreement with Dube *et al.*,^44^ the double bond conjugated with the ketone group with the hydrogen *ortho* (H2) and *para* (H4) to the nitrogen of pyridine exhibits a long-range push–pull effect, which is evidence of the *E* or *Z* geometry of exocyclic C=C. In the ^1^H NMR spectrum, isomer *E*-7-*E* (H2) is at low frequency from *Z*-7, and the H-4 in isomer *E*-7 is shifted compared to that in *Z*-7, while in the less coplanar systems (Fig. 2), MLGs 8–12 have the opposite assignment. In general, it is possible to determine all hydrogens as well as the molar fraction of each isomer (the *E*/*Z* relationship: 7, 7.2/2.8; 8, 1/0; 9, 8.5/1.5; 10, 8.2/1.8; 11, 1/0; 12, 1/0). The most evident assignments without overlapping hydrogens are the labels H2 and H4 in the NMR spectra of Fig. 2. The carbon–carbon double bond geometry was confirmed by 1D nuclear Overhauser enhancement experiments.

**Fig. 3:**
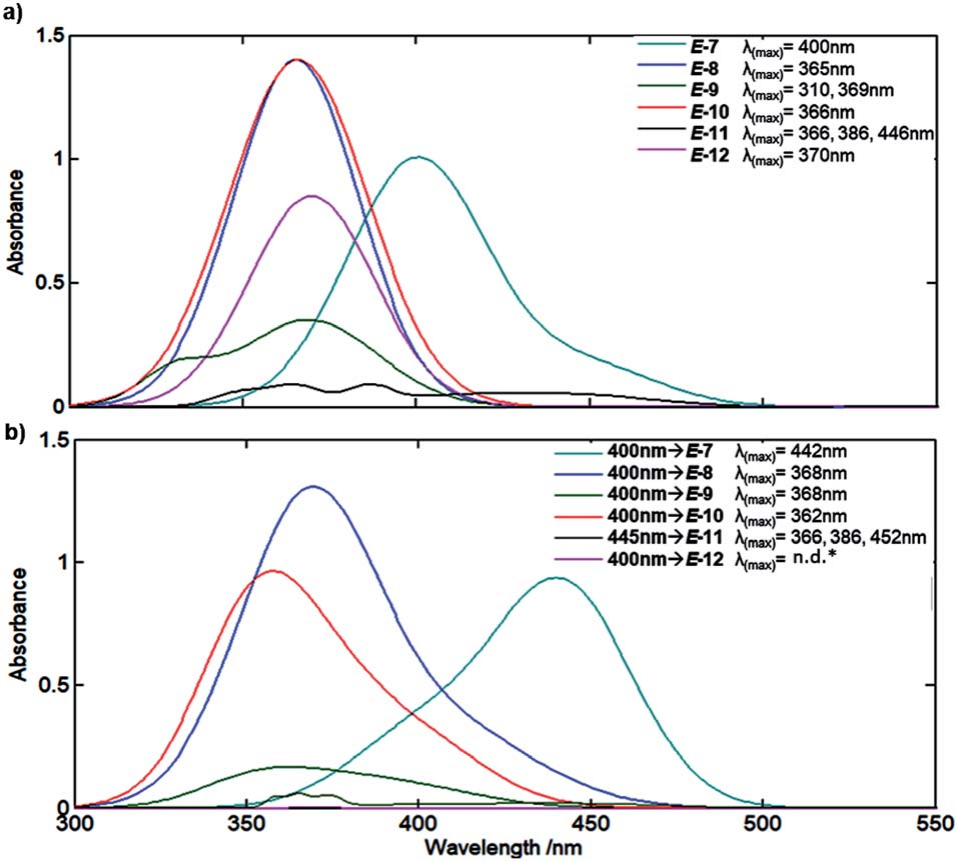
(a) Absorption UV-Vis of MLG *E*-7–*E*-12 in solution of CHCl_3_ at room temperature with concentration of 5 × 10^−5^ M. (b) Absorption UV-Vis of MLG *E*-7–*E*-12 after 8 min of irradiation with light in solution of CHCl_3_ at room temperature with concentration of 5 × 10^−5^ M. *n.d. Not determinated.

Since the aim is to prepare MLGs that operate in the visible region, we determined the extinction coefficients and maximum wavelength of the absorption of all molecules when C=C has an *E* configuration^45^ (Fig. 3). The MLGs have two transitions, n → π* and π → π*, as previously described by de Vivie-Riedle *et al.*^46^ for hemithioindigo compounds. The preliminary theoretical estimation (ESI†) and experimental data show the transitions of the ground to the excited states. MLGs *E*-7, *E*-8, *E*-10 and *E*-12 have a similar transition and band shape in the absorption spectrum; the maximum absorption band and the longer wavelength correspond to the π → π* transition, the other important transition happened to n → π*, which is a low band in the shorter wavelength. MLG 9 shows a maximum absorption signal at 310 nm and 369 nm. The transition of 369 nm is assumed with π → π* (HOMO–LUMO); the transition of 310 nm is π → π* of the other orbital π localized in the phenol part. These two transitions have an overlap band, which is related to the coplanarity of the system. In the case of MLG 11, the maximum absorption band at 446 nm correspond to the π → π* HOMO–LUMO transition in the anthracene fragment; the next wavelength at 369 nm and 310 nm correspond to the n → π* transition, the n orbital to the carbonyl group of LUMO π* of the anthracene, and has an overlap with the important π → π* transition of the HOMO–LUMO in anthracene. In the majority of cases of this MLG, the π* LUMO corresponds to the antiphase of the isomerizable double bond; this transition allows the isomerization. Notice that the UV/Vis absorption and NMR spectra are the mixtures of the two isomers *E* and *Z*; the relation depended on the medium, temperature, and stimuli.

### Study of the MLG operation

Based on the spectral data of Fig. 3, all prepared compounds interact with light at 400 nm and/or 445 nm; for this reason, we performed a rapid test by irradiating at these wavelengths for 8 minutes (irradiation was generated with a microphotochemical reactor).^47^ As summarized in Fig. 3, compound *E*-7 showed a bathochromic shift from 400 nm for the *E* isomer to 442 nm, as well as a hypochromic effect at the maximum absorption band. In turn, compounds *E*-8 and *E*-10 showed bathochromic and hypsochromic shifts, respectively, with broadening of the bands after irradiation at 400 nm, and the hypochromic effect was more marked for *E*-10 than *E*-8. Compound *E*-9 exhibited a hypochromic effect as well as broadening and changes in the band form after irradiation at 400 nm. MLG *E*-11 showed a bathochromic shift (see ESI†), broadening and a slight change in the band after irradiation at 445 nm (the wavelength was changed because *E*-11 was the first molecule to be analyzed by NMR, whereby isomerization was more clearly observed at 445 nm). On the other hand, the signals of the absorption spectrum of *E*-12 vanish after irradiation at 445 nm and 400 nm. The follow-up of the isomerization was performed through NMR experiments as described in the method section.

**Fig. 4:**
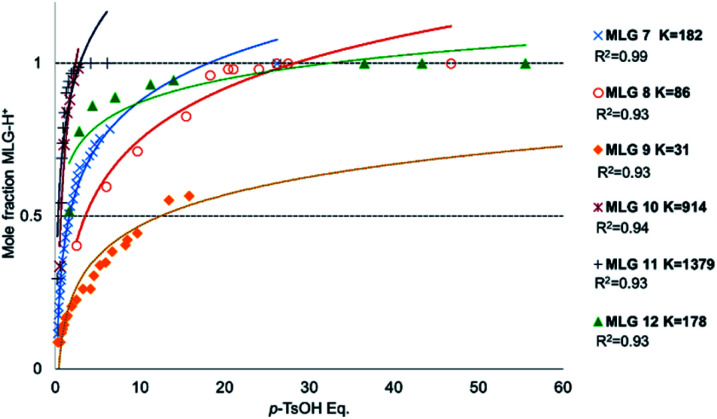
Graphics of mole fraction of MLG with *p*-TsOH solution. Each dot was analysed by ^1^H NMR spectroscopy recorded at 500 MHz in CDCl_3_. At room temperature, the concentration of MLG was between 0.005 M and 0.003 M. *K* calculated with local analysis.^49^

Protonation of the pyridine fragment was studied with ^1^H NMR in CDCl_3_ by titrating with *p*-TsOH. Graphics showing the determination of the chemical equilibrium for the protonation of the pyridine fragment in all MLGs are shown in Fig. 4. The *K* values were calculated according to Macomber,^48^ local analysis^49^ and Thordarson^50^ methods (Fig. 4 and ESI†).

The equilibrium constants values allow us to conclude that 11 and 10 show stronger interactions, that 7 and 12 exhibit moderate rates, and that 8 and 9 are the least protonated (Fig. 4).

After unambiguous characterization, the six MLGs were evaluated under different conditions (irradiation with light and chemical inputs) to understand the input dynamics. Note that only MLGs 7, 11, and 12 are discussed in detail, while 8, 9, and 10 are included in the Experimental determination of MLG operation section of the ESI† because they are similar to 7.

**Scheme 3:**
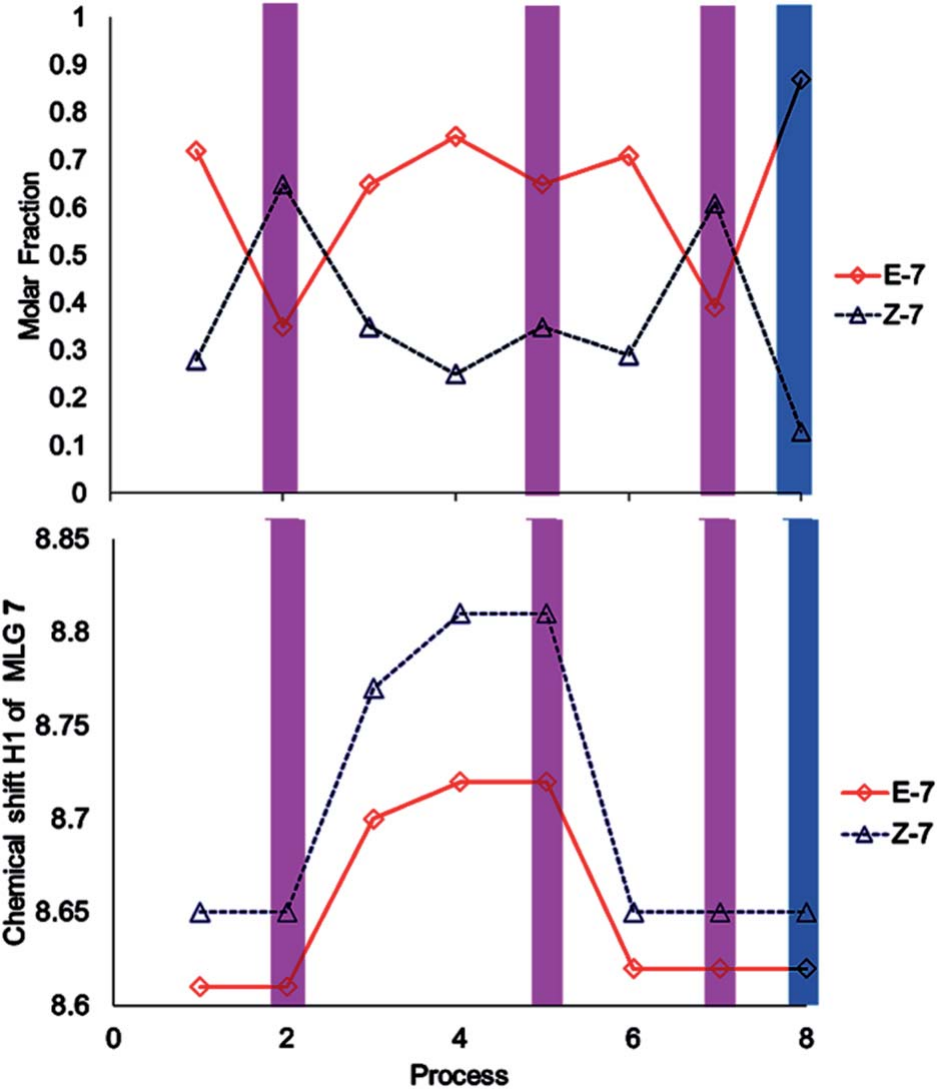
Operation cycle of MLG 7. Each process was used in these experiments was 2 mM. Process 1: start; processes 2, 5 and 7: 10 min irradiation at 400 nm (violet); process 3: 12 eq. *p*-TsOH; process 4: 15 eq. *p*-TsOH; process 6: 33.33 eq. TEA; process 8: 10 min at 445 nm (blue).

The performance of compound 7 used as a logic gate was evaluated by ^1^H NMR (Fig. 2), and the process was repeated twice (process 2, Scheme 3). Thus, irradiation at 400 nm for 10 min induced *E* to *Z* isomerization, where the latter isomer was obtained as the main product (65%). Then, 12 equivalents of a *p*-TsOH solution were added to observe partial *Z* to *E* reversal (process 3, Scheme 3), as evidenced by the shifts in the signals corresponding to H2 from the pyridine fragment of both isomers; these hydrogens turned out to be the key for the analysis of all MLGs. Further transformation of the *Z* isomer was attained by the addition of another 15 equivalents of *p*-TsOH, in which a shift in the resonance of both isomers was observed (process 4, Scheme 3). The acidic solution was irradiated at 400 nm for 10 minutes, and minor *E* to *Z* isomerization was observed in comparison with the initial solution without *p*-TsOH (process 5, Scheme 3). To verify that protonation is the origin of this effect, the solution was neutralized with triethylamine and irradiated at 400 nm for 10 minutes to detect the isomerization of compound 7 from *E* to *Z*, giving results similar to those obtained in the absence of *p*-TsOH (process 7, Scheme 3). Subsequent irradiation at 445 nm allowed *Z* to *E* isomerization (process 8, Scheme 3), thus completing the operation cycle of MLG 7.

**Scheme 4:**
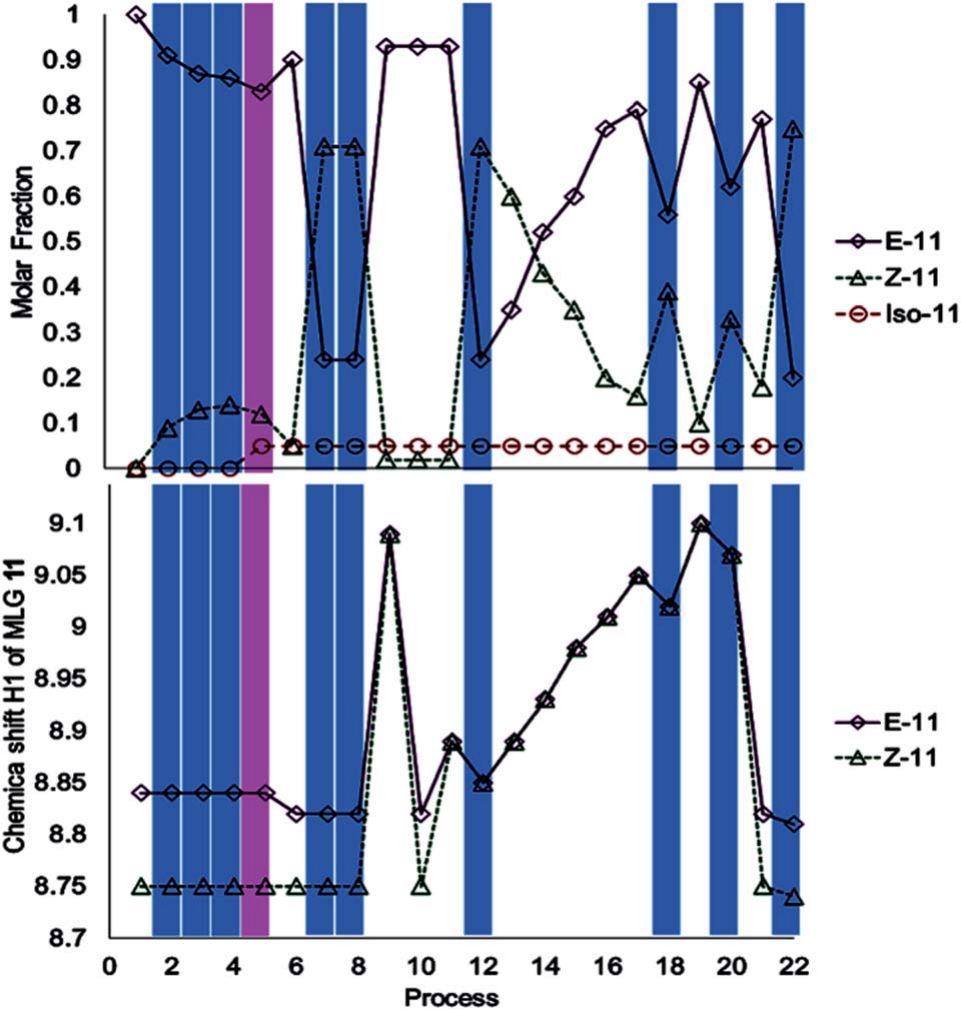
Operation cycle of MLG 11. Each process was recorded by NMR at 500 MHz in CDCl_3_ at room temperature. The concentration of MLG used in these experiments was 2 mM. Process 1: start; process 2: 2 min at 445 nm; process 3: 5 min at 445 nm; processes 4, 7, 12, 18, 20 and 22: 10 min at 445 nm; process 5: 20 min at 400 nm; process 6: 1 eq. TEA; process 8: 5 min at 445 nm; process 9: 2 eq. *p*-TsOH; process 10: 2.5 eq. *p*-TsOH; process 11: 2.5 eq. TEA; process 13: 3 eq. *p*-TsOH; process 14: 3.5 eq. *p*-TsOH; process 15: 4 eq. *p*-TsOH; process 16: 4.5 eq. *p*-TsOH; process 17: 5 eq. *p*-TsOH; process 19: 5.5 eq. *p*-TsOH; process 21: 12 eq. TEA. Blue irradiation at 445 nm; violet irradiation at 400 nm.

The investigation of MLG 11 started with irradiation at 445 nm for 17 minutes, followed by irradiation with 400 nm light for 20 minutes (processes 2–5, Scheme 4). Surprisingly, isomerization did not occur; instead, irradiation at 400 nm led to the appearance of a new NMR signal at *δ*_H_ = 4.00 (process 5, Scheme 4), which corresponded to another compound, as confirmed by mass spectrometry (ESI†). The addition of an equivalent of triethylamine under irradiation-initiated isomerization; under these conditions, it could be expected that the MLG is basic enough to react with methanol. Next, the sample was irradiated at 445 nm for 15 minutes to obtain the *Z* isomer as the major product (process 8, Scheme 4), followed by the addition of two equivalents of *p*-TsOH to obtain the *E* isomer as the major product (process 9, Scheme 4). The solution was neutralized with 2.5 equivalents of triethylamine to verify that it corresponded to the *E* isomer, as further confirmed by ^1^H NMR (process 10, Scheme 4). The addition of 0.5 equivalents of *p*-TsOH followed by two consecutive irradiations with 445 nm light for 10 minutes resulted in a lower degree of *E* to *Z* isomerization than that achieved with process 7 (process 12, Scheme 4 and process 17, Scheme 4). Further addition of 5.5 equivalents of *p*-TsOH increased the ratio of the *E* isomer, as evidenced by the ^1^H NMR signals of the isomer, which shifted to higher frequencies (process 19, Scheme 4). This last sample was irradiated at 445 nm for 10 minutes, resulting in a lower degree of isomerization than that in entry 7 (process 22, Scheme 4). This step was carried out to finish the operation cycle of MLG 11, but note that irradiation with 400 nm light did not lead to *E* or *Z* isomerization.

The study of MLG 12 started with irradiation at 445 nm, followed by irradiation at 400 nm. In this case, each irradiation led to decomposition, and the procedure was repeated 5 times. The addition of *p*-TsOH did not provide the initial isomers, and NMR revealed the presence of MLG 12 only. These results allowed us to conclude that the MLG decomposed under irradiation with light (ESI†).

**Scheme 5:**
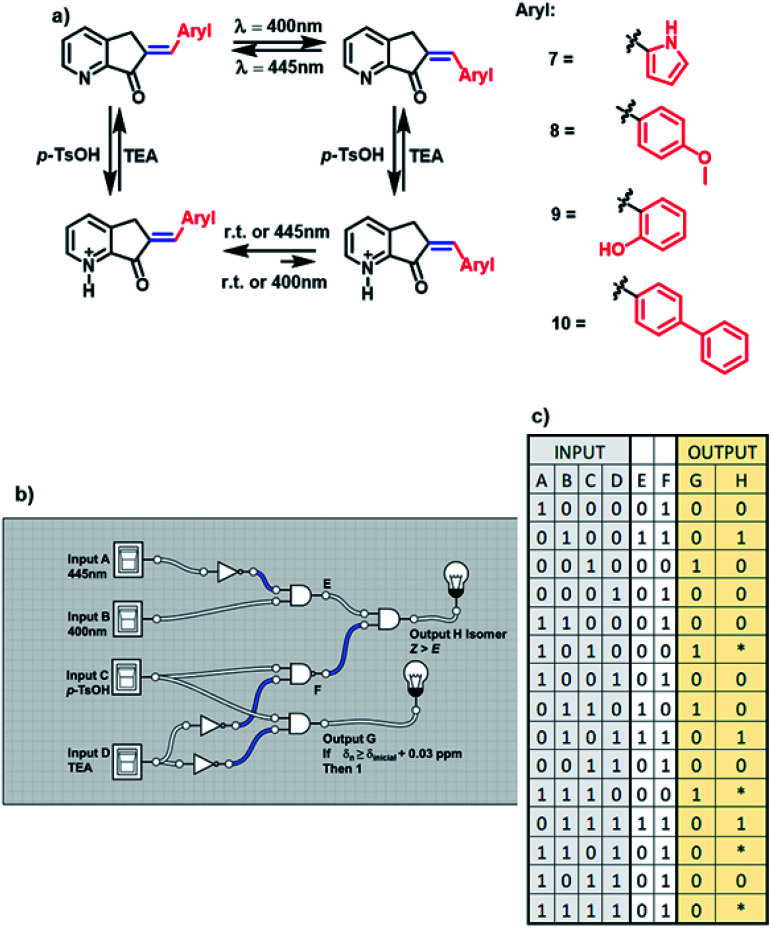
(a) Operation cycles of MLGs 7, 8, 9 and 10. (b) Diagram representation of the logic gates of the complex circuit generated from the MLGs. (c) Table of the digitalization of the inputs and outputs through the logic operations.

As mentioned above, MLGs 7, 8, 9, and 10 had the same operation cycle when *E* to *Z* isomerization was carried out with light at 400 nm, and isomerization was reversible at 445 nm. Upon the addition of acid, the MLGs are protonated, and their equilibrium is shifted toward the *E* isomer. Note that the more equivalents of the proton source added, the greater the shift to the *E* isomer. Another characteristic that should be highlighted is that when these protonated MLGs are irradiated with 400 nm light, the *E* isomer is favored. In contrast, irradiation at 445 nm provides the *Z* isomer in small or almost null ratios (figure a, Scheme 5).

As the functions of these MLGs are similar, they are capable of performing the same logic operations. In agreement with the convention of the analog to the digital system, in the case of the MLG, the inputs, as well as the outputs, are assigned to experimental conditions^19^ as 0 or 1. These MLGs (7, 8, 9 and 10) have four inputs:

Input *A* is assigned as blue light irradiation with 445 nm LEDs.

Input *B* is assigned as violet light irradiation with 400 nm LEDs.

Input *C* is the addition of *p*-TsOH.

Input *D* is the addition of trimethylamine.

Intermediate values are *E* and *F*.

The absence of these factors is denoted 0, and the presence of these inputs is denoted 1. Solving the logic system provides two outputs. For output *G*, when the protonation of the compound induces a shift in the signal of the *ortho* protons of at least 0.03 ppm, in the ^1^H NMR spectrum, the response is denoted 1. If no change in the chemical shift is observed, as the response is denoted 0. Output *H* is the response to *E* to *Z* isomerization, and in this condition, the *Z* isomer should be the major product. These conditions were used to build the MLG. The relationship between input *A* and input *B* is determined by the logic gate of INH A; in this case, input *A* carries out the logic process called NOT (when the answer is 0 and 1 if there is a response) before reaching the logic gate AND, and the answer is the next matrix. Input *C* and input *D* have the logic gate relation INH D, where input *D* carries out the logic process NOT before the logic gate AND, giving the matrix shown in table c of Scheme 5. This logic gate gives output *G*.

To relate the four inputs, the relationship between the logic gate AND changes for NAND between input *C* and input *D* so that the output of this logic operation is related to the result of the logic gate of input *A* and input *B*. The logic gate that connects these two outputs is AND, where the result of this logic gate gives output *H* (diagram b, Scheme 5).

The matrix of table c and Scheme 5 summarize the responses. An important factor of this logic gate is related to the methodology; the two wavelengths cannot be applied at the same time, and thus, the response of output *H* depends on the order in which irradiation is done. For this reason, in the matrix of Scheme 5, table c, the asterisk in the matrix (not 1 or 0) refers to the dependence on the applied time of the inputs.

**Scheme 6:**
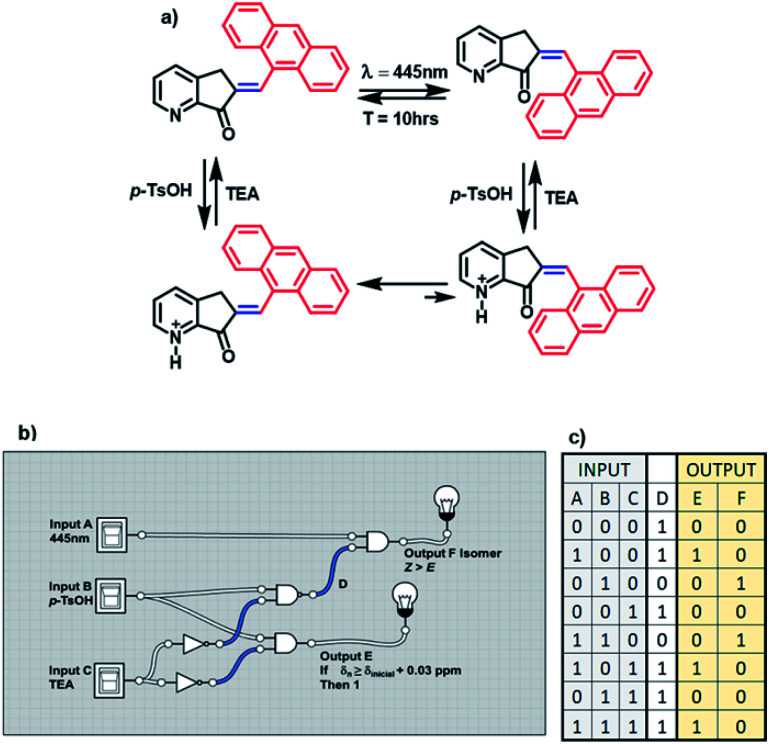
(a) Operation cycle of MLG 11. (b) Diagram representation of the logic gates of the complex circuit created by the MLGs. (c) Table of the digitalization of the inputs and outputs through the logic operations.

MLG 11 is different because its operation cycle does not exhibit *Z* to *E* isomerization induced by light, although it slowly isomerizes at room temperature after 10 h. Similarly, this MLG shifts to the *E* isomer upon increasing the equivalents of protons added (figure a, Scheme 6). For this reason, 11 has three inputs. Input *A* is assigned to blue light, input *B* is due to the addition of *p*-TsOH, and input *C* is the addition of triethylamine.

The presence (1) and absence (0) of inputs are defined as previously stated, and the response of the logic gate is two outputs. Output *F* induces shifts of H2 greater than 0.03 ppm in the ^1^H NMR spectrum upon protonation with an acid (denoted 1), while the absence of any shift in the NMR spectrum is denoted 0. Output *E* is the response to *E* to *Z* isomerization if the major product is isomer *Z*. Input *B* and input *C* are related to the logic gate INH C, where input *C* has the operation NOT before making the logic gate AND, and the result is output *F*.

The relationship between these three inputs is that input *A* is directly related to the result of the operation between inputs *B* and *C*. To relate it with input *A*, it is necessary to change the logic operations AND for NAND and relate the output with input *A* using the logic gate AND, and the result is output *E*. Finally, MLG 11 only has *D* as the intermediate value (diagram b, Scheme 6).

**Scheme 7:**
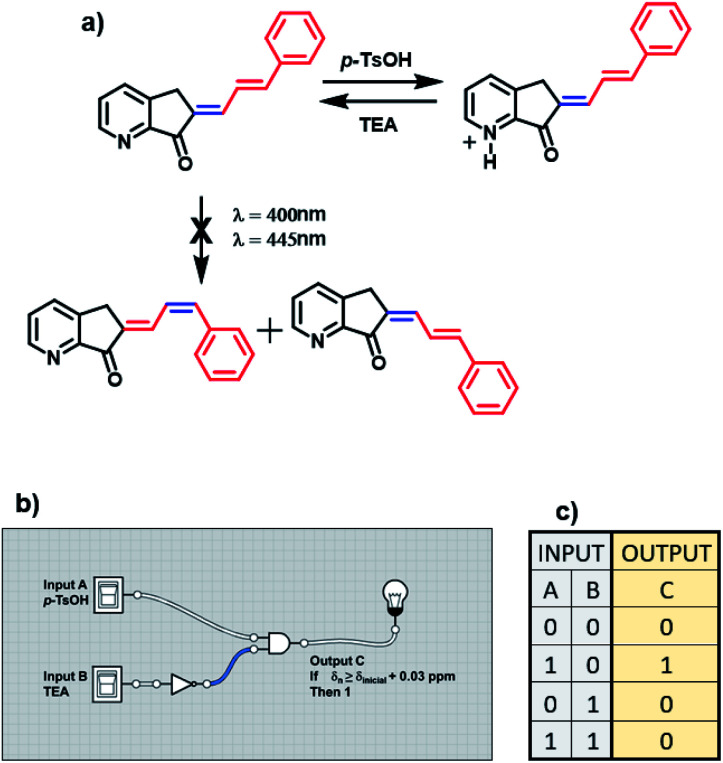
(a) Operation cycle of MLG 12. (b) Diagram representation of the logic gates of the complex circuit created by the MLGs. (c) Table of the digitalization of the inputs and outputs through the logic operations.

It is impossible to perform a light logic operation with MLG 12 because this compound decomposed; for this reason, it only depends on protonation (figure a, Scheme 7). Therefore, it only features the INH molecular logic gate. Input *A* is the addition of acid, and input *B* is the addition of triethylamine (denoted 1 for the presence and 0 for the absence). The resulting output of this MLG is output *C*, with the condition that the *ortho* and *para* hydrogens are shifted more than 0.03 ppm in the ^1^H NMR spectrum upon protonation (denoted 1). As mentioned above, the relationship between input *A* and input *B* is the molecular logic gate INH B, where input *B* undergoes the nonlogic operation before performing the AND logic gate operation (diagrams b and c, Scheme 7).

**Scheme 8:**
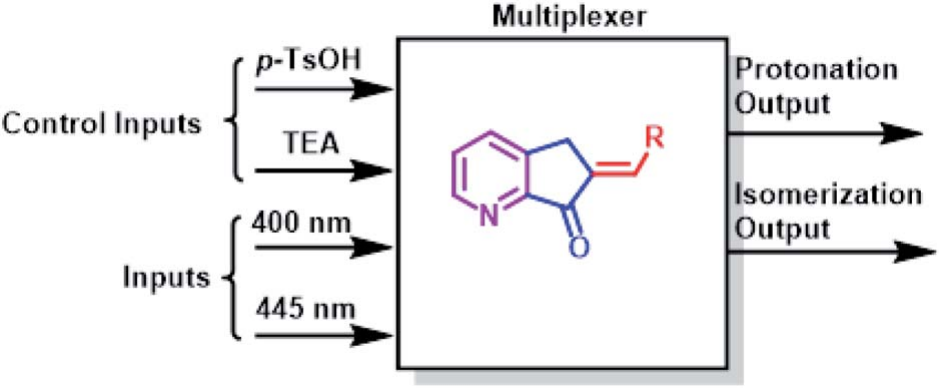
The individual operation of MLG 7–11 as a multiplexer.

The operation processes of MLGs 7, 8, 9, 10, and 11 were analyzed individually. First, 4 to 3 inputs are considered that give 2 outputs (4 or 3 : 2 inputs : outputs). Second, *p*-TsOH and TEA are the control inputs because the related outputs depend on the response to these inputs to solve the logic operation and interference directly on the operation of the light inputs. Therefore, the total response outputs control and inform the system based on *p*-TsOH/TEA inputs. For example, if the system had an excess of *p*-TsOH, MLGs *E*-7 to *E*-11 could not undergo isomerization with light inputs. The operation process of these MLGs can be described as multiplexers (Scheme 8). MLG 12 can only be a basic logic gate and, in this case, an INH gate.

Based on the operation pattern of the MLG, an experiment was proposed to create a more complex system using two MLGs with two different logic operations, 7 and 11, in the same NMR tube.

**Scheme 9:**
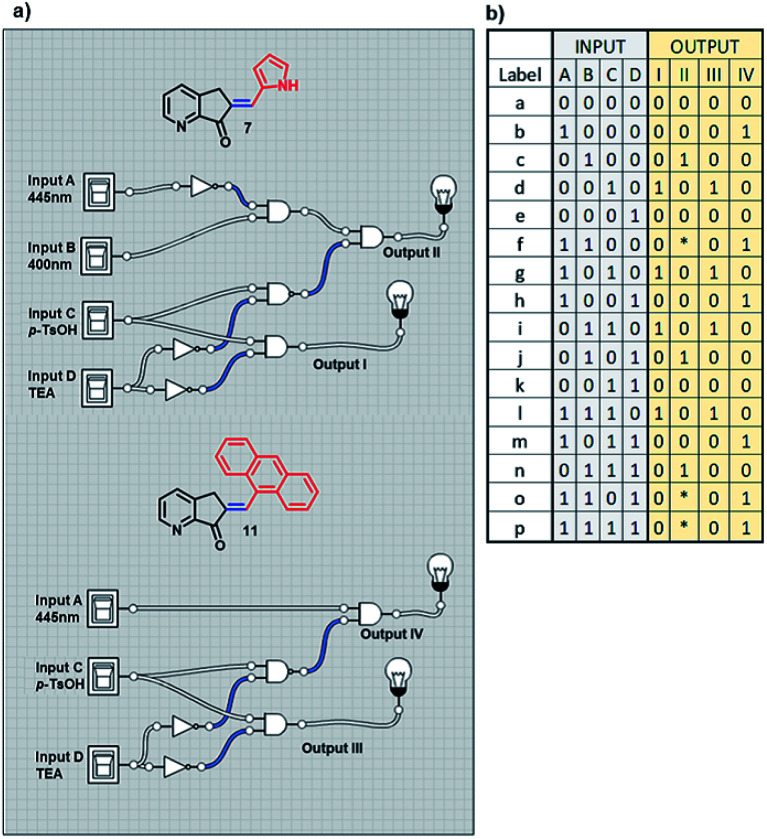
(a) Diagram of the logic gates of MLGs 7 and 11. (b) The table with the theoretical response obtained by Logicly and the inputs of the different sequence of addition.

With previous knowledge of the individual operation patterns of MLGs 7 and 11, the solution to the logic operation of this MLG was estimated, and the matrix was generated with Logicly software^51^ (table b, Scheme 9). The elements that define the matrix of Scheme 9 are as follows. Both MLGs have four inputs: input *A* is 445 nm irradiation for 10 minutes, input *B* is 400 nm irradiation for 7 minutes, input *C* is the addition of *p*-TsOH, and input *D* is the addition of triethylamine. These four inputs used in different sequences go through the logic operation created by the MLG, giving four outputs as a response: output I is the response to the *E* to *Z* isomerization of MLG 7, output II is the response to the protonation of MLG 7, output III is the response to the *E* to *Z* isomerization of MLG 11, and output IV is the response to the protonation of MLG 11. With these inputs, different addition sequences are processed for MLGs 7 and 11, giving a solution with different patterns of the four outputs (diagram a, Scheme 9).

**Scheme 10:**
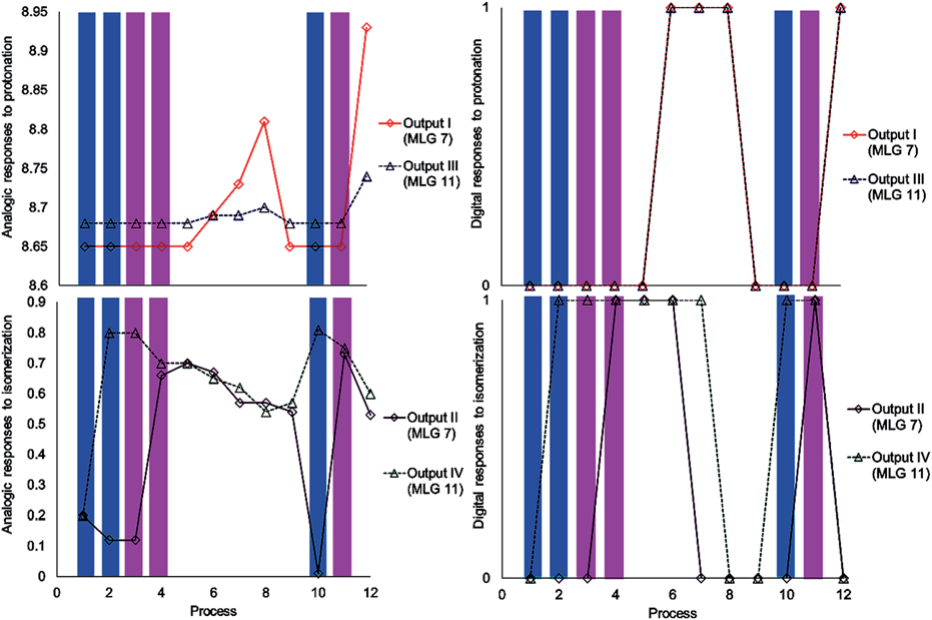
Graphics of the digitalization (left: analogic and right; digital) of the process made in each experiment. The concentrations of MLGs 7 (output I and II) and 11 (output III and IV) were 1.5 mM in CDCl_3_ : DMSO-d_6_ (3 : 0.2 volume ratio), and each process is described in Scheme 11.

**Scheme 11:**
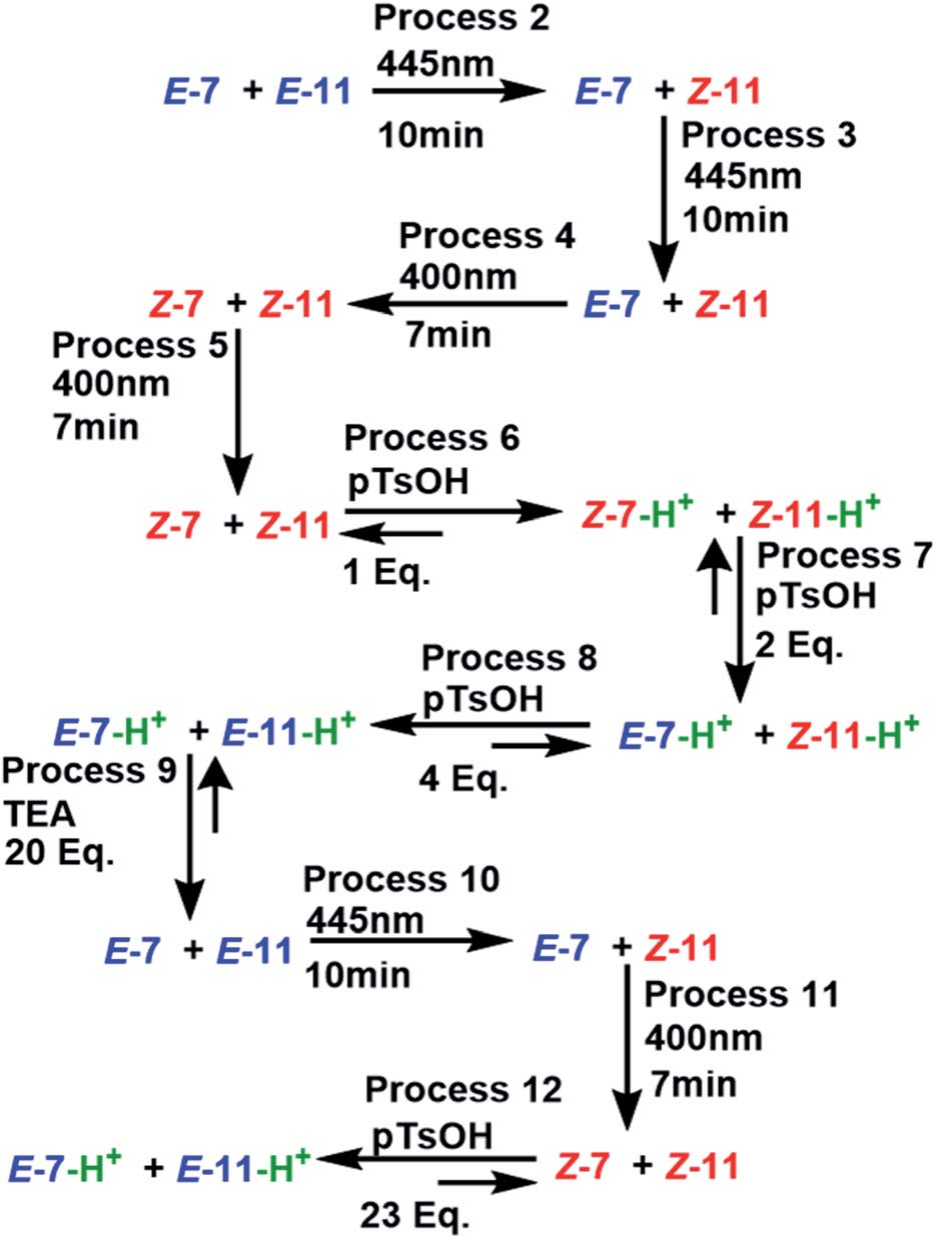
Processes applied to the mixture of MLG 7 and 11. Molecules in red indicate a 0 value of outputs II and IV, blue molecules correspond to the 1 value of outputs II and IV, the absence of protons is represented as 0 for outputs I and III, the presence of protons (green) corresponds to the 1 value of outputs I and III.

The experiment started with the verification of the NMR chemical shift of the major isomer, which in MLGs 7 and 11 is *E* (process 1, Schemes 10 and 11). Next, the sample was irradiated with blue light (445 nm) for 10 minutes, and only 11 underwent *E* to *Z* isomerization, with the next irradiation at 445 nm giving the same response (processes 2 and 3, Schemes 10 and 11). Next, irradiation with 400 nm light for 7 minutes led to the *E* to *Z* isomerization of 7, and further irradiation (7 min) maintained *Z*-7 and *Z*-11 as the major isomers (processes 4 and 5, Schemes 10 and 11). The major *Z* isomer in both MLGs was treated with one equivalent of *p*-TsOH (twice) and analyzed by ^1^H NMR. After the addition of four equivalents of *p*-TsOH, *Z* to *E* isomerization and shifts in the NMR signals to a high frequency were observed; with this process, both MLGs had an equilibrium of approximately 50% *Z* and *E* isomers (processes 6, 7, and 8, Schemes 10 and 11). With the addition of triethylamine, the proton NMR chemical shifts of both MLGs returned to their initial values. Further irradiation with 445 nm light for 10 minutes isomerized MLG 11 from *E* to *Z* and completed the isomerization of MLG 7 from *Z* to *E* (Processes 10, Schemes 10 and 11). The sample was then irradiated with violet light for 7 minutes to obtain *Z*-7 (Process 11, Schemes 10 and 11). The *Z* isomer was the major product in both MLGs, and *Z* to *E* isomerization was achieved after the addition of 23 equivalents of *p*-TsOH (Process 12, Schemes 10 and 11).

The next step of this experiment was data digitalization and identification of the dynamic range of the response, denoted 0 and 1. In these experiments, the output that depends on isomerization is 1 when the fraction of *Z* is as high as 0.6, and the output that depends on the chemical shift should be 1 when the NMR signal of one of the isomer signals shifts at least 0.01 ppm. Scheme 10 shows how digitalization is determined.

An important step of digitalization is to generate the matrix of Table S2 (ESI†), which corresponds to how the order of the addition of the inputs in each process affects the outputs. Next to the outputs are the labels that relate the theoretical table to the experimental results (table b, Scheme 9). In Schemes 10, 11 and in Table S2,† the solutions to the logic operation of the MLG correspond to the inputs (switch on) and the agreement with the outputs, where the focus is on obtaining a positive response.

**Scheme 12:**
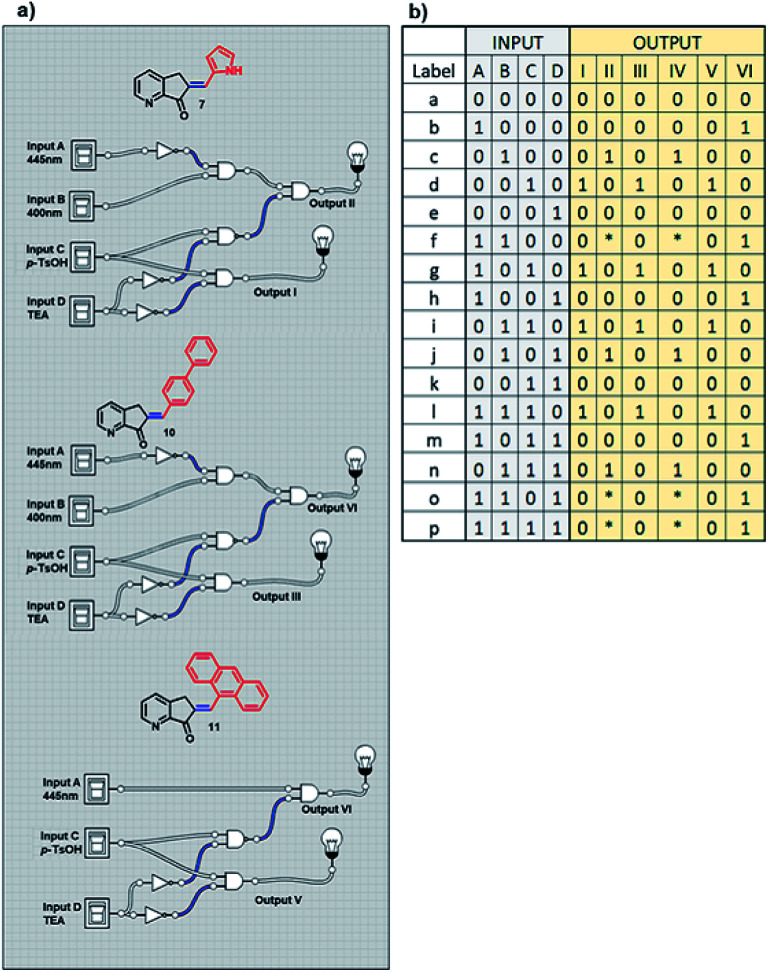
(a) Diagram of the logic gates of MLGs 7, 10 and 11. (b) Table with the theoretical reply obtained by Logicly and the inputs of the different patterns of addition.

The next experiment involved three MLGs (7, 10, and 11) in the same NMR tube. The matrix in table b, Scheme 12 was calculated according to the operation of each individual MLG,^51^ and the possible truth table of responses to input additions can be solved, generating 6 outputs. Analysis of the responses of the three MLGs results in a demultiplexer. As in the previous experiment, there are 4 inputs, which when submitted to the conditions give 6 outputs: output I is the *E*–*Z* isomerization of MLG 7, output II is the protonation of MLG 7, output III is the *E*–*Z* isomerization of MLG 10, output IV is the protonation of MLG 10, output V is the *E*–*Z* isomerization of MLG 11 and output VI is the protonation of MLG 11 (diagram a, Scheme 12).

**Scheme 13:**
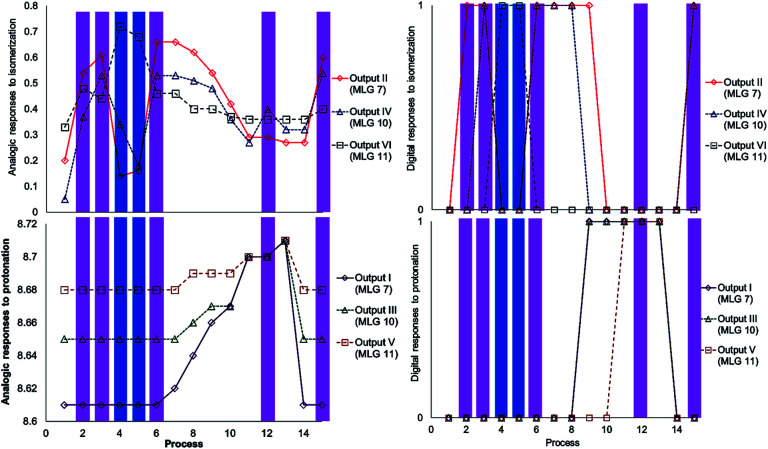
Graphics of digitalization (analogic graphics at left, digital graphics at right) of the processes carried out in each experiment. The concentrations of MLGs 7 (output I and II), 10 (output III and IV) and 11 (output V and VI) were 1.5 mM in CDCl_3_ : DMSO-d_6_ (3 : 0.2 volume ratio), and each process was recorded by NMR at 500 MHz. The description of each process is given in Scheme 14.

**Scheme 14:**
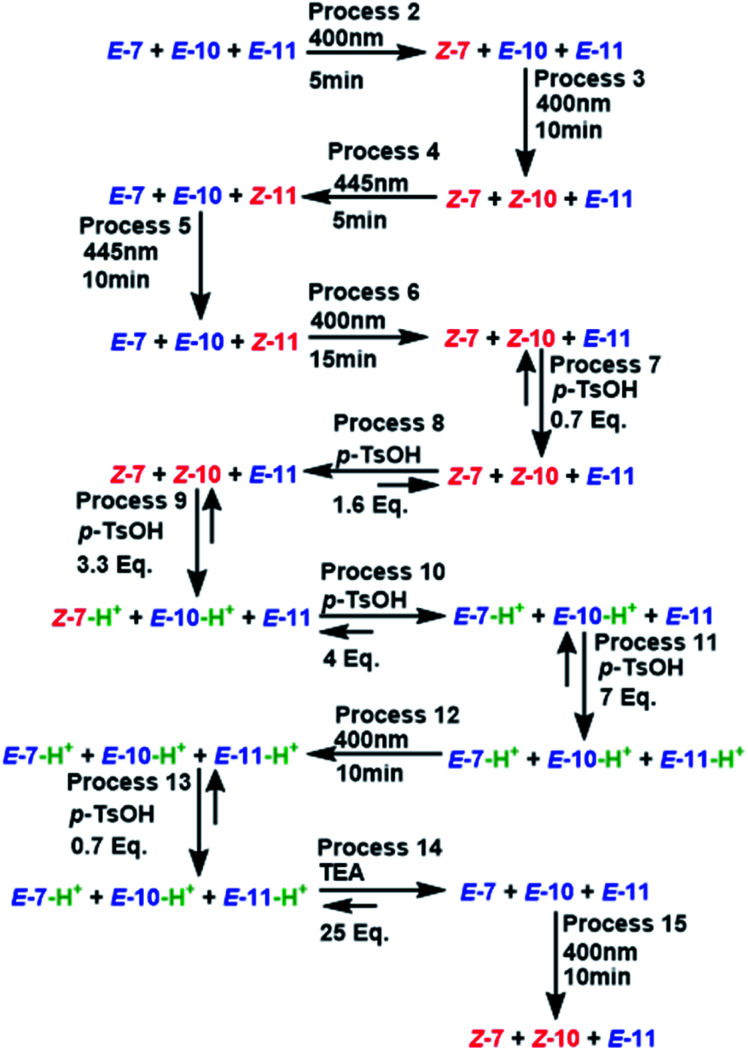
All processes applied to the mixture of MLG 7, 10 and 11. The molecules in red represent the 0 value of outputs II, IV and VI; the molecules in blue represent the 1 value of outputs II, IV and VI. The absence of protons represents the 0 value of outputs I, III and V, the presence of proton (green) represents the 1 value of outputs I, III and V.

The initial state of the sequence of inputs of these three MLGs was analyzed by NMR, showing that the *E* isomer is a major product under neutral conditions (process 1, Schemes 12 and 13). The sequence started by performing irradiation at 445 nm for 15 minutes, resulting in the *E*–*Z* isomerization of MLGs 7 and 10, while 11 remained unchanged (processes 2 and 3, Schemes 13 and 14). Then, the solution was irradiated with 445 nm light for 15 minutes, which caused the *E*–*Z* isomerization of 11, while *Z*7 and *Z*10 underwent *Z*–*E* isomerization. This process afforded *E*7, *E*10 and *Z*11 as the major products (processes 4 and 5, Schemes 13 and 14). Once the MLGs were mostly in the *Z* geometry, 0.7 equivalents of *p*-TsOH were added, and proton NMR spectra were recorded, showing no significant changes in the signals of these three MLGs (process 7, Schemes 13 and 14). The addition of 1.6 equivalents of *p*-TsOH shifted the signals of the three MLGs as a result of *Z*–*E* light-induced isomerization (process 8, Schemes 13 and 14). After the addition of 3.3 equivalents of *p*-TsOH, the ^1^H NMR signals shift to a high frequency, in agreement with *Z*–*E* isomerization. In particular, the resonance of 7 is shifted to a high frequency, and 10 and 11 have the *E* isomer as the main product (process 9, Schemes 13 and 14). When 7 equivalents of *p*-TsOH were added, the *E* isomer was the major product for the three compounds. In addition, the ^1^H chemical shift was observed at a high frequency (process 11, Schemes 12 and 13). After protonation of the three MLGs, the solution was irradiated with violet light for 10 minutes to induce *Z*–*E* isomerization; however, only partial isomerization of 10 was observed, while 11 and 7 maintained the same isomer ratio (process 12, Schemes 13 and 14). When no further *E*–*Z* isomerization was induced by light, 10.5 equivalents of *p*-TsOH were added to obtain the *Z* derivative as the major product in the three MLGs (process 13, Schemes 13 and 14), as confirmed by the ^1^H chemical shift, which appeared at a high frequency. To achieve complete isomerization, 25 equivalents of triethylamine were added, and the solution was irradiated at 400 nm for 10 minutes to induce *E*–*Z* isomerization of 7 and 10 (process 15, Schemes 13 and 14).

As in the previous experiment, the data were digitalized, with responses of 0 and 1. In this experiment, the output that depends on isomerization is 1 when the major isomer is *Z*, and the output that depends on the ^1^H NMR chemical shift is 1 when the signal is shifted by at least 0.02 ppm (Scheme 13).

An important part of digitalization is to generate the matrix of Table S4 (ESI†); in the experiment, this corresponds to how the order of the addition of the inputs of each process affects the outputs. Placed at one side of the outputs is the label that relates the theoretical table with the experiment.

The experiment shows how the inputs of each process are added and the responses (outputs). In addition to the outputs, the labels that relate to table b of Scheme 12 are theoretically created with experimental data. Additionally, Table S4 and Scheme S5 in the ESI† show how the logic operations of the MLGs are solved by adding the inputs that turn on the switches and how the responses coincide with the outputs that we proposed.

**Scheme 15:**
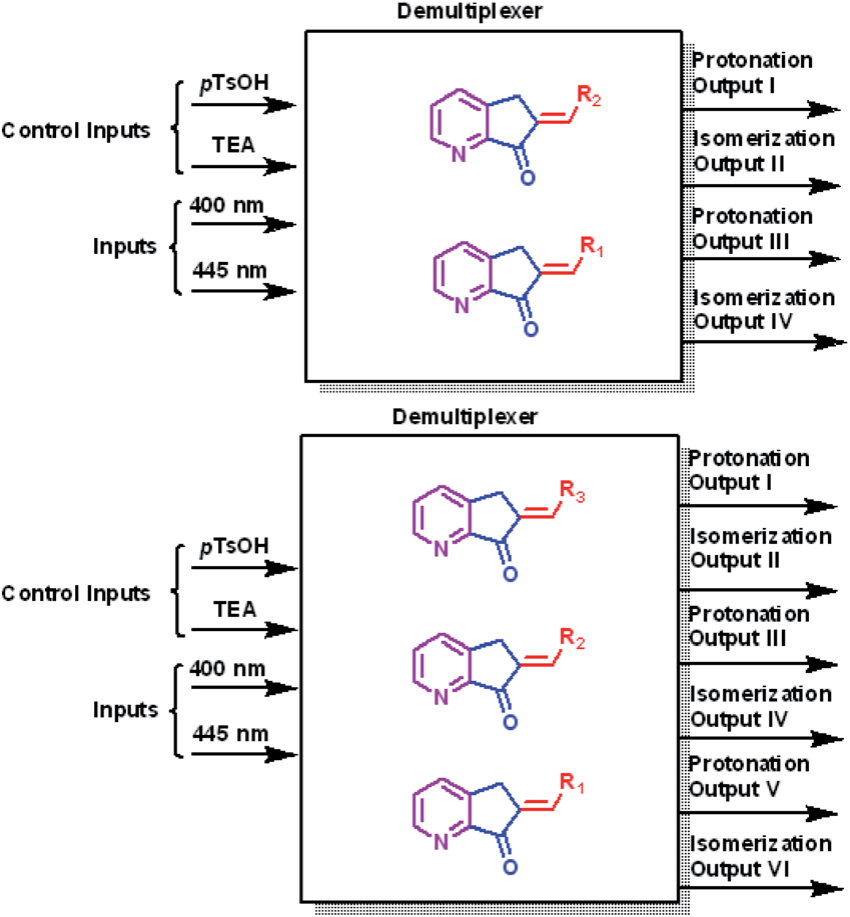
The general diagram of the two systems of multiple MLG as demultiplexers.

With this information, multiple MLG systems were analyzed to assign the type of combined circuit. The first system has 4 inputs for 4 outputs (4 : 4 inputs : outputs), and the second system has 4 inputs for 6 outputs (4 : 6 inputs : outputs). The two MLG systems, as individual MLGs, have *p*-TsOH/TEA inputs as control inputs, and the responses of all outputs depend on the state of control inputs; the state of *p*-TsOH/TEA inputs affects the processing of light inputs. Since the two systems have the control inputs/inputs and have a pattern with an equal number of or more outputs than inputs, the two multiple MLG systems can be assigned as demultiplexers (Scheme 15).

## Conclusions

MLGs were synthesized using simple molecules that can function as complex logic gates. Compounds 7, 8, 9 and 10 are MLGs of the same type that work with two light inputs (445 nm and 400 nm) and with protonation inputs. MLG 11 operates only with a light input (445 nm) and with protonation inputs. MLG 12 operates solely with protonation inputs. MLGs 7-11 can operate individually as multiplexors, and with the systems of multiple MLGs, demultiplexers can be constructed. With this protocol, we illustrate the possibility of creating more molecular complex systems and molecular combined circuits with small and simple MLGs. NMR is a useful technique to study these processes, even when the analyzed systems contain two to three MLGs, providing high precision and more information regarding the behavior of the system. ^1^H NMR analysis of systems with a combination of MLGs allowed us to conclude that NMR is the best tool to study the dynamics of this type of molecular switch. These types of MLGs could have many applications, for example, as specific intracellular indicators or chemical sensors. It has been suggested that this type of MLG with two inputs can function as a real logic gate, as suggested by Yang *et al.*^52^

## Experimental

### Spectra

The NMR spectra were recorded at 21.1 °C using a Jeol ECA-500 system at *B*_0_ = 11.75 T (^1^H at 500.159 MHz and ^13^C at 125.76 MHz). All spectra were recorded in a CDCl_3_ solution in 5 mm OD tubes.

The structures of the compounds were assigned using the pfg-COSY, pfg-HMBC and pfg-HSQC and DPFGSE-NOE pulse sequences.^53,54^

The UV-Vis spectra were determined using a PerkinElmer Lambda 2S spectrometer. Mass spectra were recorded with ESI-API.

### NMR titration

*p*-TsOH was added with a micropipette to the solution of the corresponding MLG in CDCl_3_ at 3 mM in a 5 mm OD NMR tube, and the ^1^H NMR spectrum was recorded at 500 MHz.

### Operation cycle determination

In a 5 mm OD NMR tube, a solution of the corresponding MLG at the indicated concentration was prepared. The NMR tube was irradiated with an Aldrich® Micro photochemical reactor; the scheme can be found in the ESI.† The NMR tube was placed close to the LEDs using an aluminum tube to maintain a firm vertical position. Blue irradiation at a wavelength of 445 nm and violet irradiation at a wavelength of 400 nm were used. All ^1^H NMR spectra were recorded at 500 MHz.

### Synthesis

In a 15 mL vial, 0.37 mmol of pyrrol-2-carboxaldehyde, 0.37 mmol of ketone 6, and 4 mL of ethanol were placed, and the mixture was stirred at room temperature until a homogenous solution was observed. The reaction mixture was chilled with ice, and a 5% solution of potassium hydroxide was added dropwise until a precipitate formed. Then, 5 mL of water was added, and the reaction mixture was frozen. Finally, the solution was allowed to reach room temperature and filtered under vacuum.

## Author contributions

Osvaldo J. Quintana-Romero carried out the conceptualization, formal analysis, methodology and writing – original draft. Armando Ariza-Castolo carried out the founding acquisition, project administration, resources, supervision, writing – review & editing.

## Conflicts of interest

There are no conflicts to declare.

## Supplementary Material

RA-011-D1RA00930C-s001
