# Supplementary material for: Complex molecular logic gates from simple molecules
Source: RSC Adv. 2021 Jun 14;11(34):20933–43. doi: 10.1039/d1ra00930c (PMC9034006; doi:10.1039/d1ra00930c)
Supplement: RA-011-D1RA00930C-s001 [file RA-011-D1RA00930C-s001.pdf]

Electronic Supplementary Material (ESI) for RSC Advances.
This journal is © The Royal Society of Chemistry 2021

Electronic Supplementary Information

Complex Molecular Logic Gates from Simple Molecules

Osvaldo J. Quintana-Romero, Armando Ariza-Castolo
Departamento de Química, Centro de Investigación y Estudios Avanzados del Instituto Politécnico Nacional, Av. IPN 2508, San Pedro Zacatenco, 07360, Ciudad de México.

Content:

General information	S3
Synthesis: methodology 1–3	S3
Spectra conditions	S3
Operation cycle determination methodology information	S4
Molecular logic gates	S4-S23
5,6-dihydro-7H-cyclopenta[b]pyridin-7-one (**6**)	S4
^{1}H NMR spectra of compound **6**	S5
(E)-6-((1H-pyrrol-2-yl)methylene)-5,6-dihydro-7H-cyclopenta[b]pyridin-7-one (**7**):	S6
^{1}H and ^{13}C NMR spectra of compound **7**	S7-S8
(E)-6-(4-methoxybenzylidene)-5,6-dihydro-7H-cyclopenta[b]pyridin-7-one (**8**)	S9
^{1}H and ^{13}C NMR spectra of compound **8**	S10-S11
(E)-6-(2-hydroxybenzylidene)-5,6-dihydro-7H-cyclopenta[b]pyridin-7-one (**9**)	S12
^{1}H and ^{13}C NMR spectra of compound **9**	S13-S14
(E)-6-([1,1'-biphenyl]-4-ylmethylene)-5,6-dihydro-7H-cyclopenta[b]pyridin-7-one (**10**)	S15
^{1}H and ^{13}C NMR spectra of compound **10**	S16-S17
(E)-6-(anthracen-9-ylmethylene)-5,6-dihydro-7H-cyclopenta[b]pyridin-7-one (**11**)	S18
^{1}H and ^{13}C NMR spectra of compound **11**	S19-S20
(E)-6-((E)-3-phenylallylidene)-5,6-dihydro-7H-cyclopenta[b]pyridin-7-one (**12**)	S21
^{1}H and ^{13}C NMR spectra of compound **12**	S22-S23
Absorption spectrums UV/Vis of MLG after irradiation light.	S24-S26
Titration procedure	S26
Table S1. Calculated protonation constants K	S26
Figure S12. Titration of MLG **7** with pTSOH	S27
Figure S13. Titration of MLG **8** with pTsOH	S28
Figure S14. Titration of MLG **9** with pTsOH	S29
Figure S15. Titration of MLG **10** with pTsOH	S30
Figure S16. Titration of MLG **11** with pTsOH	S31
Figure S17. Titration of MLG **12** with pTsOH	S32
Figure S18–S21. NMR nOe-dpfgse spectra of MLG ***E*-11**	S33–S36

Experimental determination of MLG operation	S37
Figure S22. Image of photoirradiation experiment.	S37
Figure S23. ^{1}H NMR spectrums of compounds ***E*-7**, ***Z*-7**, and ***E*-7H⁺**.	S38
Figure S24. ^{1}H NMR spectrums of compounds ***E*-8**, ***Z*-8**, and ***E*-8H⁺**.	S39
Figure S25. ^{1}H NMR spectrums of compounds ***E*-9**, ***Z*-9**, and ***E*-9H⁺**.	S40
Figure S26. ^{1}H NMR spectrums of compounds ***E*-10**, ***Z*-10**, and ***E*-10H⁺**.	S41
Figure S27. ^{1}H NMR spectrums of compounds ***E*-11**, ***Z*-11**, and ***E*-11H⁺**.	S42
MLG operation process	S43-S48
Figure S28. Scheme of MLG **7** operation process	S43
Figure S29. Scheme of MLG **8** operation process.	S44
Figure S30. Scheme of MLG **9** operation process.	S45
Figure S31. Scheme of MLG **10** operation process.	S46
Figure S32. Scheme of MLG **11** operation process.	S47-S48
Chart S1. Proposed molecule of ***E*-12** decomposition by 400 nm light.	S48
Figure S33. Scheme of MLG **12** operation process.	S49
Description of Operation Process for MLG **8**, **9**, and **10**.	S50-S55
Scheme S1. Operation cycle of MLG **8.**	S50
Scheme S2. Operation cycle of MLG **9**.	S52
Scheme S3. Operation cycle of MLG **10**.	S54
Table S2. Summary of results for each process performed to verify MLGs **7** and **11**.	S56
Figure S34. Scheme of MLG **7** and **11** operating in the same NMR tube.	S57
Table S3. Summary of method of operation for the mixture of MLGs **7** and **11**, outputs and corresponding labels	S58
Scheme S4. Diagrams of the logical operation solved with the mixture of MLGs **7** and **11** with the theoretical labels	S59-S60
Table S4. Summary of results of each process performed to verify for the mixture of MLGs **7**, **10** and **11.**	S61
Figure S35. Operating process for MLG **7, 10** and **11**	S62
Table S5. Digitalization responses of the process and the logical operation solved for the mixture of MLGs **7**, **10** and **11**	S63
Scheme S5. Diagrams for the logical operation solved with the mixture of MLGs **7, 10** and **11** with the theoretical labels	S64-S68
Theory calculation of MLG ***E*-7**	S68-70
Figure S36. Structure of MLG E-7 refined with DFT.	S69
Table S5. Comparation data of DFT methods for ***E*-7** simulate UV/Vis absorption spectra	S69
Figure S37. Theory UV/Vis absorption spectra of ***E*-7.**	S70
Figure S38. Orbitals of transitions states of ***E*-7**	S71

General information

All reagents were purchased from Sigma-Aldrich and used without previous purification. The NMR spectra were recorder using a JEOL ECA-500 spectrometer with a magnetic field of 11.75 T (1H, 500.160 MHz; 13C, 125.765 MHz). The unified scale was used with TMS as reference diluted (volume fraction $\varphi < 1\%$) in chloroform for ^{1}H resonance ((CH3)4Si ^{1}H, ^{13}C = 0). The UV/Vis absorption spectra were recorder with a Perkin Elmer Lambda 2S UV-VIS spectrophotometer. Low resolution mass spectra were acquired with HPLC/coupled mass Agilent Technologies (ESI).

Synthesis

Methodology 1

In a round-bottom flask, 1.12 mmol of potassium hydroxide were dissolved in 12 mL of methanol under stirring and a solution of ketone **6** (0.37 mmol in 3 mL of methanol) was added dropwise followed by dropwise addition of a solution of 2-pyrrole carboxaldehyde (0.48 mmol in 3mL of methanol). The reaction mixture was maintained under vigorous stirring for 3 days at room temperature. After the reaction time, the organic phase was extracted with methylene chloride/200 mL of brine solution and concentrated under vacuum. The product was purified by chromatographic column using mixtures of Hexane:Ethyl Acetate of increasing polarity as eluent.

Methodology 2

2-Pyrrole carboxaldehyde (0.48 mmol), ketone **6** (0.37 mmol) and 0.75 mmol of sodium sulphate were placed in a 15mL vial followed by dropwise addition of 0.2 mL of piperidine and the reaction mixture was maintained under vigorous stirring agitation for 1 day at room temperature. After the reaction time, the organic phase was extracted with methylene chloride/1% hydrochloric acid aqueous solution, evaporated under vacuum and the product was purified by column chromatography using Hexane:Ethyl acetate mixtures of increasing polarity as eluent.

Methodology 3

To a 10 mL vial, 0.37 mmol of 2-pyrrole carboxaldehyde, 0.37 mmol of Ketone **6** and 0.4 mL of ethanol were added, the reaction mixture was left under stirring until a homogeneous solution was formed. Subsequently, the reaction mixture was placed in an ice bath and a 5% aqueous solution of potassium hydroxide was added dropwise until formation of a precipitate. Then 5 mL of water were added and the reaction mixture was allowed to freeze. The frozen solution was allowed to reach room temperature and filtered under vacuum.

Spectra conditions

The NMR spectra were recorded at 21.1°C using a Jeol ECA-500 at B_0=11.75 T (^{1}H at 500.159 MHz and ^{13}C at 125.76 MHz). All spectra were recorded in $CDCl_3$ solution in 5 mm OD tubes. The compounds were assigned using the pfg-COSY, pfg-HMBC and pfg-HSQC pulse sequences.The UV-Vis spectra were determined using a Perkin Elmer Lambda 2S spectrometer; to obtain the coefficient absorptions we did a standard curve (Concentration/Absorption) with 3 concentrations ($3X10^{-5}$M, $5X10^{-5}$M, $7X10^{-5}$M) and then

we could calculate the absorption epsilon with slope. The Mass spectra were recorded with ESI-API.

Operation cycle determination methodology information

In a 5 mm OD NMR tube, a solution of the corresponding MLG in the concentration indicated was prepared. The NMR tube was irradiated with an Aldrich® Micro Photochemical Reactor. The NMR tube was placed close to the LEDs using an aluminum tube to maintain a firm vertical position. Blue irradiation with a wavelength of 445 nm and violet irradiation with a wavelength of 400 nm were used. All ^{1}H NMR spectra were recorded at 500 MHz.

Molecular logic gates

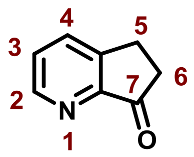

6

5,6-dihydro-7H-cyclopenta[b]pyridin-7-one (6):

^{1}H NMR ($CDCl_3$, 500 MHz): **H2** δ 8.77 (1H, d, *J* = 3.8 Hz), **H4** δ 7.90 (1H, d, *J* = 7.4 Hz), **H3** δ 7.45 (1H, dd, *J* = 4.5, 10.0 Hz), **H6** δ 3.17 (2H, m, *J* = 6.3 Hz), **H5** δ 2.77 (2H, m).

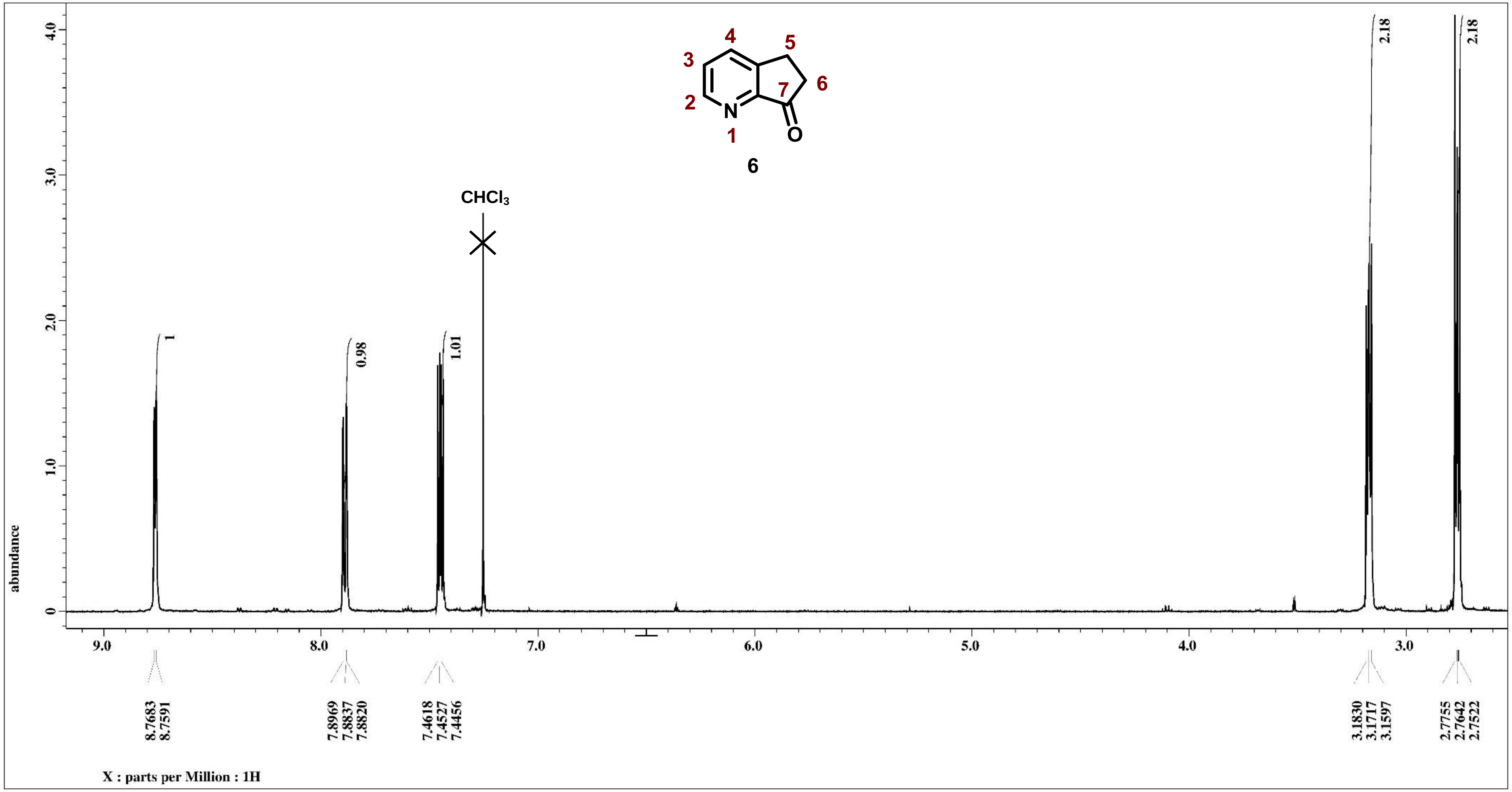

4.0
3.0
2.0
1.0
0
abundance
1
0.98
1.01
2.18
2.18
CHCl3
6
9.0
8.0
7.0
6.0
5.0
4.0
3.0
8.7683
8.7591
7.8969
7.8837
7.8820
7.4618
7.4527
7.4456
3.1830
3.1717
3.1597
2.7755
2.7642
2.7522
X : parts per Million : 1H

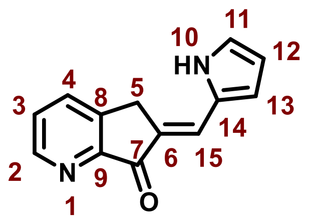

PLM *E*-7

(E)-6-((1H-pyrrol-2-yl)methylene)-5,6-dihydro-7H-cyclopenta[b]pyridin-7-one (7):

^{1}H NMR (DMSO d6, 500 MHz): **H10** δ 11.62 (1H, s), **H2** δ 8.68 (1H, dd, *J* = 1.4. 4.5 Hz), **H4** δ 8.06 (1H, dd, *J* = 1.4, 7.7 Hz), **H3** δ 7.54 (1H, dd, *J* = 4.5, 7.7 Hz), **H15** δ 7.53 (1H, s), **H11** δ 7.17 (1H, hex, *J* = 1.4 Hz), **H13** δ 6.74 (1H, s), **H12** δ 6.32 (1H, m), **H5** δ 3.94 (2H, s).

^{13}C {^{1}H} NMR ($CDCl_3$, 125 MHz): δ **C7** 192.03, **C9** 156.06, **C2** 150.54, **C6** 144.71, **C4** 135.58, **C14** 129.03, **C3** 127.60, **C8** 127.46, **C15** 125.31, **C11** 124.89, **C13** 115.50, **C12** 112.29, **C5** 30.40.

ES-API *m/z*: M^{+} 211.0.

Absorption coefficient in $CHCl_3$: 21000 cm^{-1}

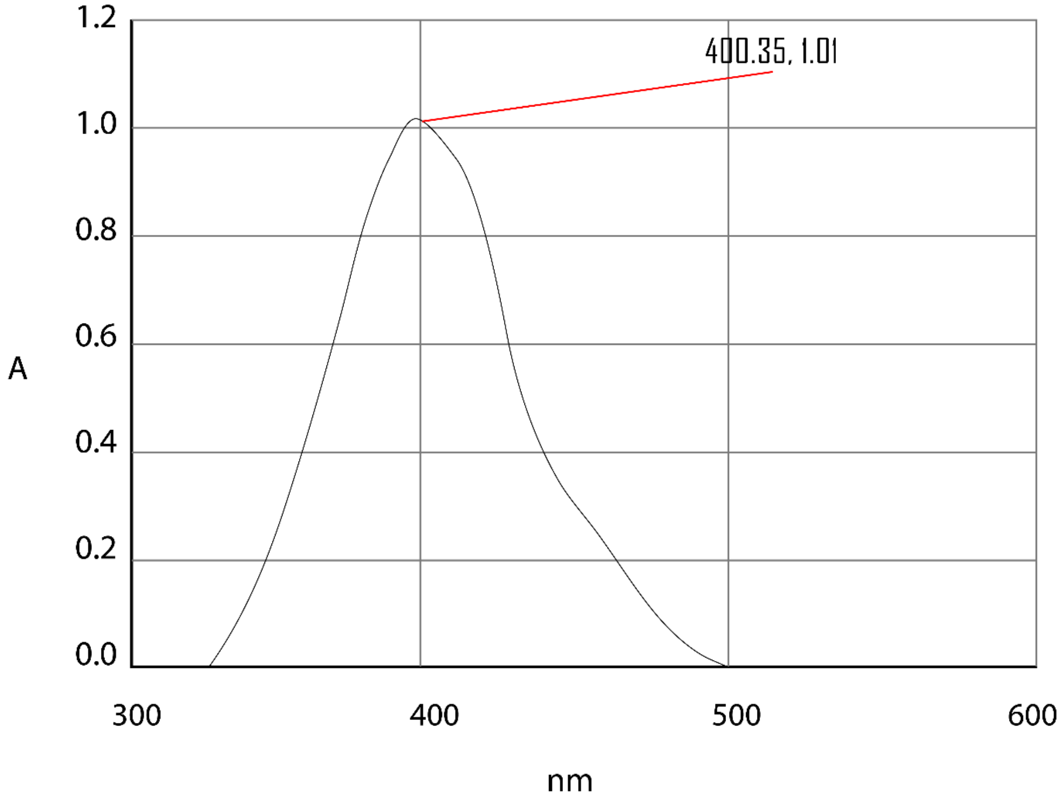

Figure S1. Absorption UV/Vis spectrum of ***E*-7** in $CHCl_3$ (with DMSO drops) at room temperature with concentration of $5X10^{-5}$ M.

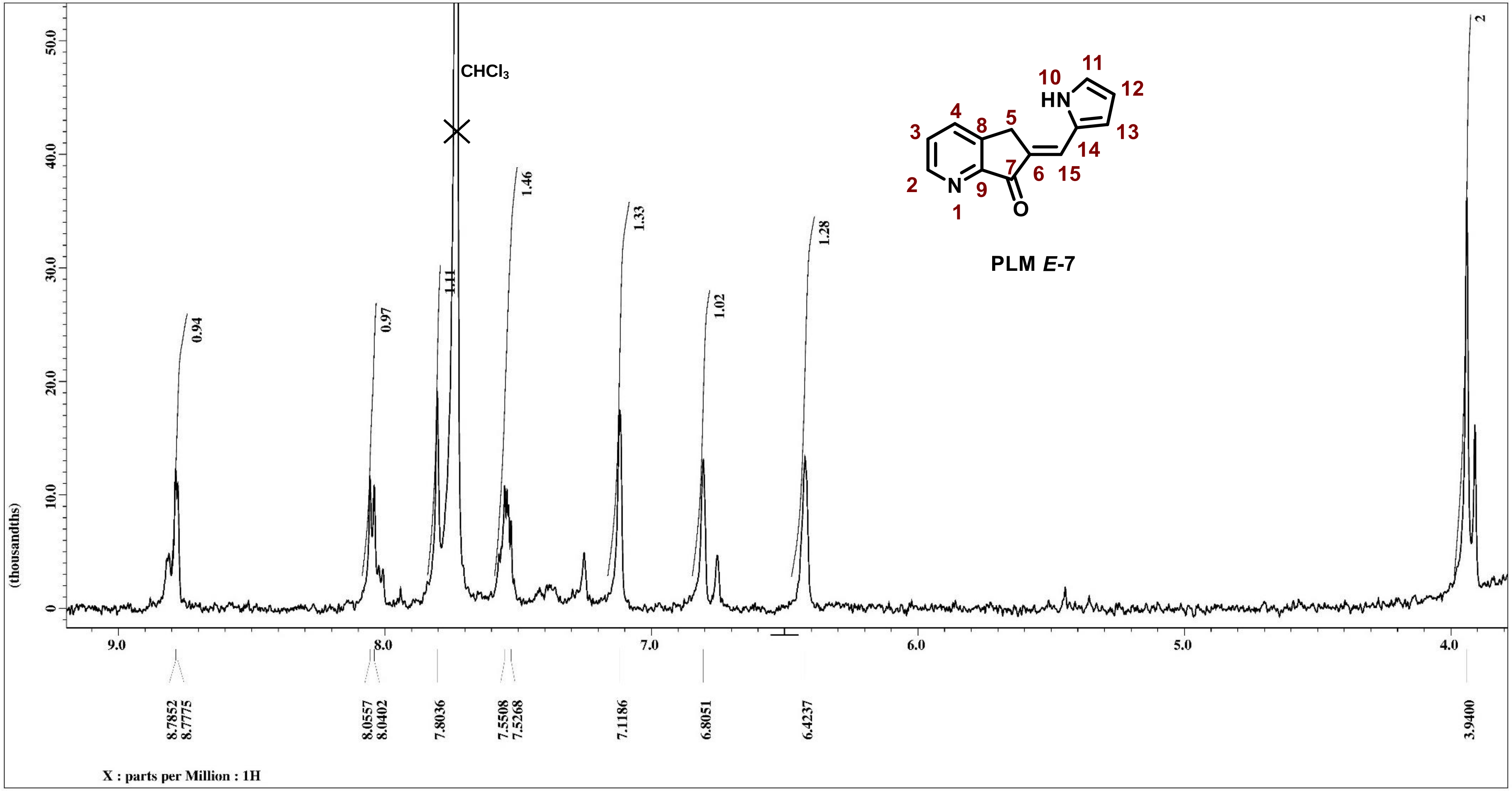
CHCl3
PLM E-7
X : parts per Million : 1H
(thousandths)
0.94
0.97
1.11
1.46
1.33
1.02
1.28
2
8.7852
8.7775
8.0557
8.0402
7.8036
7.5508
7.5268
7.1186
6.8051
6.4237
3.9400

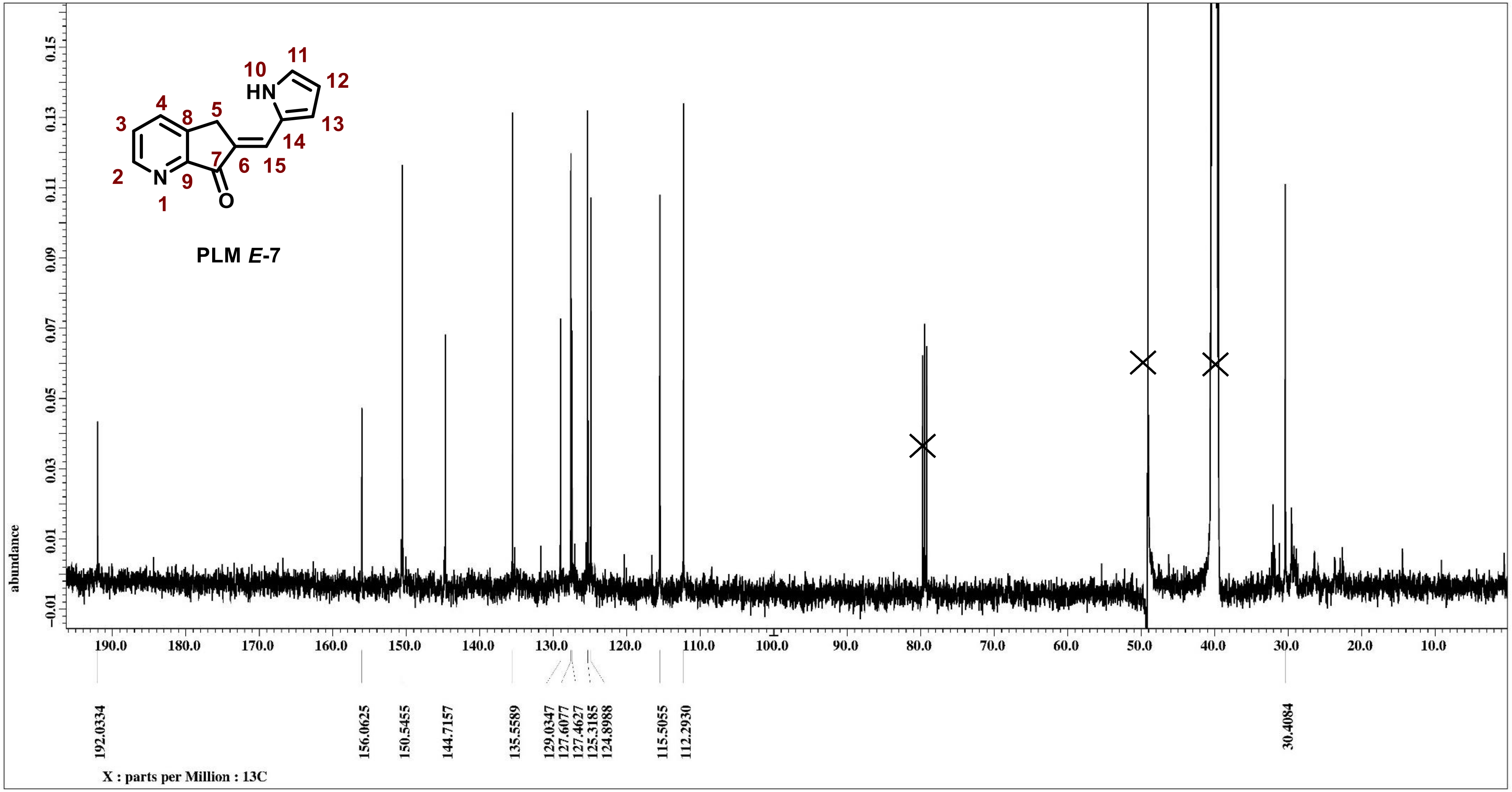

PLM E-7
HN
N
O
abundance
X : parts per Million : 13C
192.0334
156.0625
150.5455
144.7157
135.5589
129.0347
127.6077
127.4627
125.3185
124.8988
115.5055
112.2930
30.4084

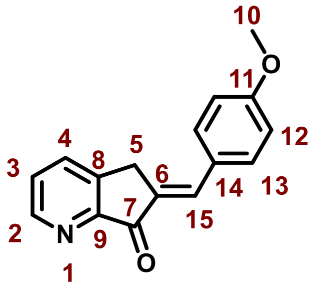

PLM *E*-8

(E)-6-(4-methoxybenzylidene)-5,6-dihydro-7H-cyclopenta[b]pyridin-7-one (8):

^{1}H NMR (DMSO d6, 500 MHz): **H2** δ 8.68 (1H, dd, *J* = 1.4. 4.5 Hz), **H4** δ 8.14 (1H, dd, *J* = 1.4, 7.7 Hz), **H13** δ 7.71 (2H, d, *J* = 8.8 Hz), **H15** δ 7.66 (1H, s), **H3** δ 7.61 (1H, dd, *J* = 4.5, 8.1 Hz), **H12** δ 7.03 (2H, d, *J* = 8.8 Hz), **H5** δ 4.06 (2H, s), **H10** δ 3.84 (3H, s).

^{13}C {^{1}H} NMR ($CDCl_3$, 125 MHz): **C7** δ 193.74, **C11** 162.39, **C9** 154.77, **C2** 150.05, **C6** 145.48, **C15** 136.00, **C4** 135.80, **C13** 133.28, **C14** 130.80, **C3** 127.87, **C8** 127.46, **C12** 114.39, **C10** 54.87, **C5** 29.85.

ES-API *m/z*: M^{+} 252.2.

Absorption coefficient in $CHCl_3$: 28000 cm^{-1}

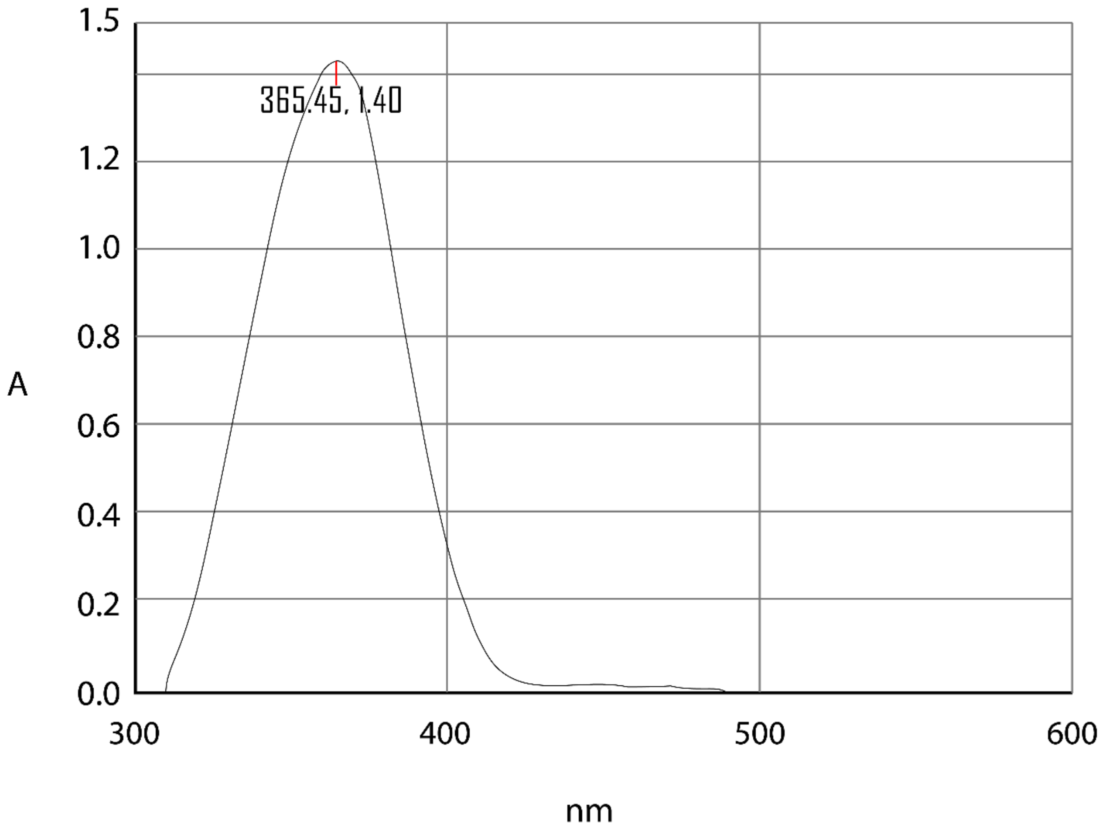

Figure S2. Absorption UV/Vis spectrum of ***E*-8** in $CHCl_3$ at room temperature with concentration of 5X10^{-5} M.

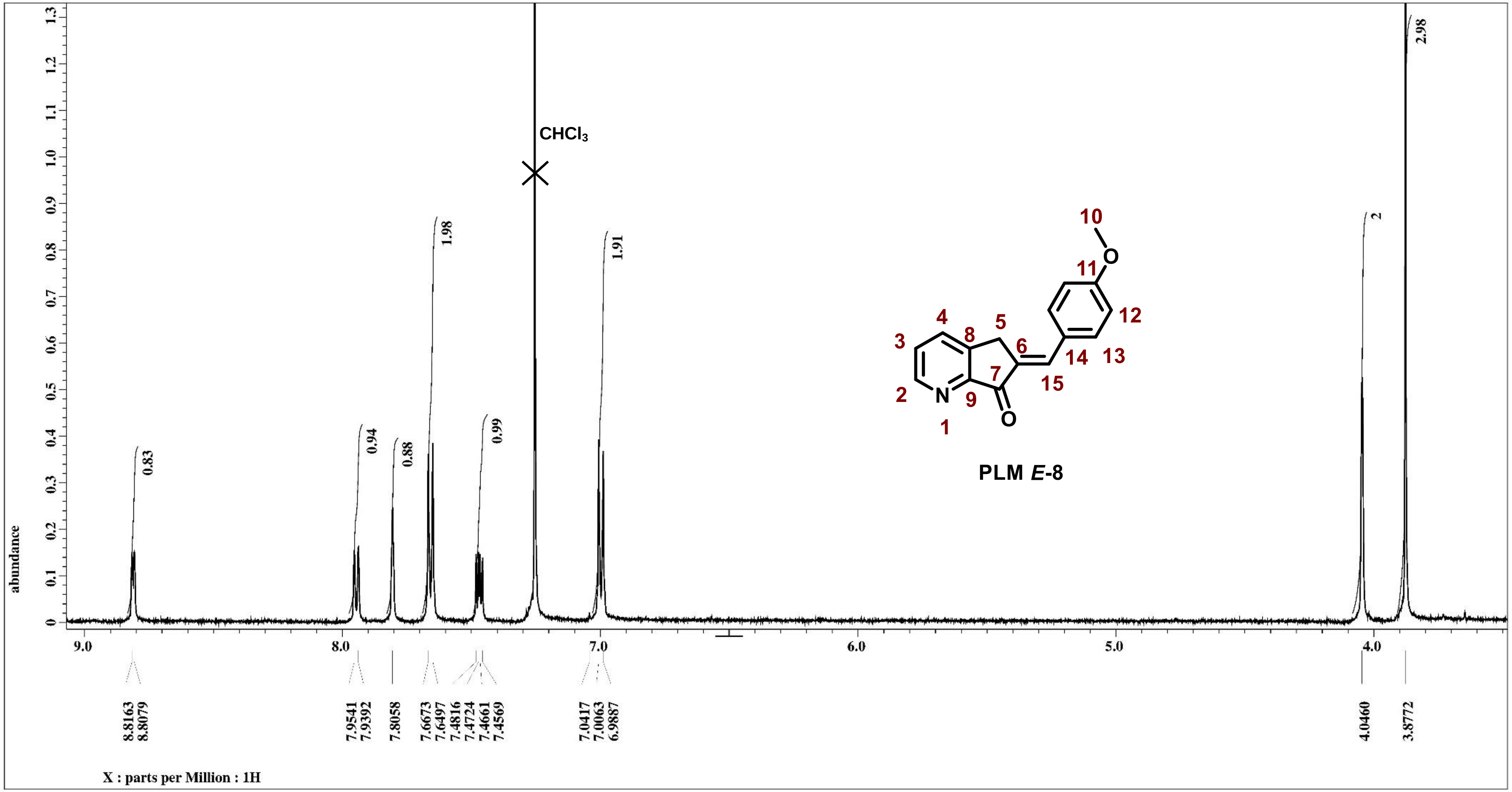
CHCl$_3$
PLM *E*-8
abundance
X : parts per Million : 1H
0.83
0.94
0.88
1.98
0.99
1.91
2
2.98
8.8163
8.8079
7.9541
7.9392
7.8058
7.6673
7.6497
7.4816
7.4724
7.4661
7.4569
7.0417
7.0063
6.9887
4.0460
3.8772

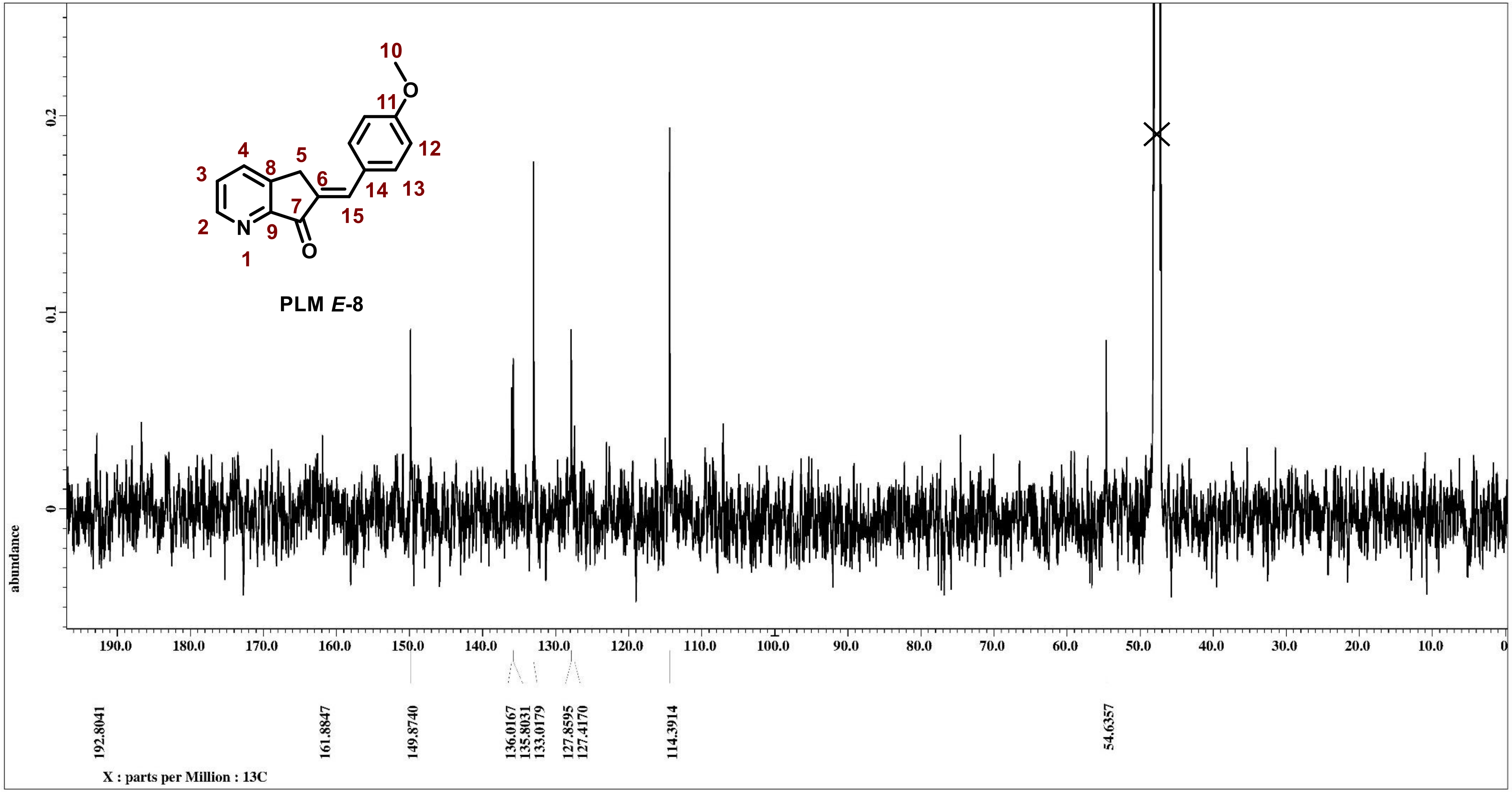
PLM E-8
abundance
0.2
0.1
0
190.0
180.0
170.0
160.0
150.0
140.0
130.0
120.0
110.0
100.0
90.0
80.0
70.0
60.0
50.0
40.0
30.0
20.0
10.0
192.8041
161.8847
149.8740
136.0167
135.8031
133.0179
127.8595
127.4170
114.3914
54.6357
X : parts per Million : 13C

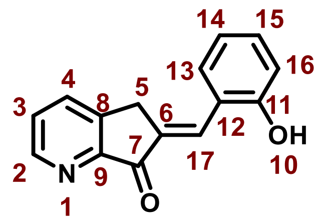

PLM *E*-9

(E)-6-(2-hydroxybenzylidene)-5,6-dihydro-7H-cyclopenta[b]pyridin-7-one (9):

^{1}H NMR (CD_3OD, 500 MHz): **H2** δ 8.65 (1H, dd, *J* = 1.4. 4.5 Hz), **H17** δ 8.52 (1H, s), **H4** δ 8.10 (1H, dd, *J* = 1.4, 7.7 Hz), **H13** δ 7.61 (1H, dd, *J* = 1.4, 8.1 Hz), **H3** δ 7.55 (1H, dd, *J* = 4.5, 7.7 Hz), **H15** δ 7.10 (1H, td, *J* = 2.1, 8.4 Hz), **H16** δ 6.70 (1H, dd, *J* = 1.0, 8.4 Hz), **H14** δ 6.51 (1H, t, *J* = 7.4 Hz), **H5** δ 3.32 (2H, s). [1]

^{3}C {^{1}H} NMR ($CDCl_3$, 125 MHz): δ **C7** 193.17, **C11** 170.13, **C9** 155.49, **C1** 149.43, **C6** 144.95, **C17** 135.84, **C4** 135.51, **C15** 132.68, **C13** 129.78, **C8** 127.52, **C2** 127.15, **C12** 123.63, **C16** 120.69, **C14** 114.29, **C5** 29.93.

ES-API *m/z*: M^+ 238.3.

Absorption coefficient in $CHCl_3$: 10000 cm^{-1}

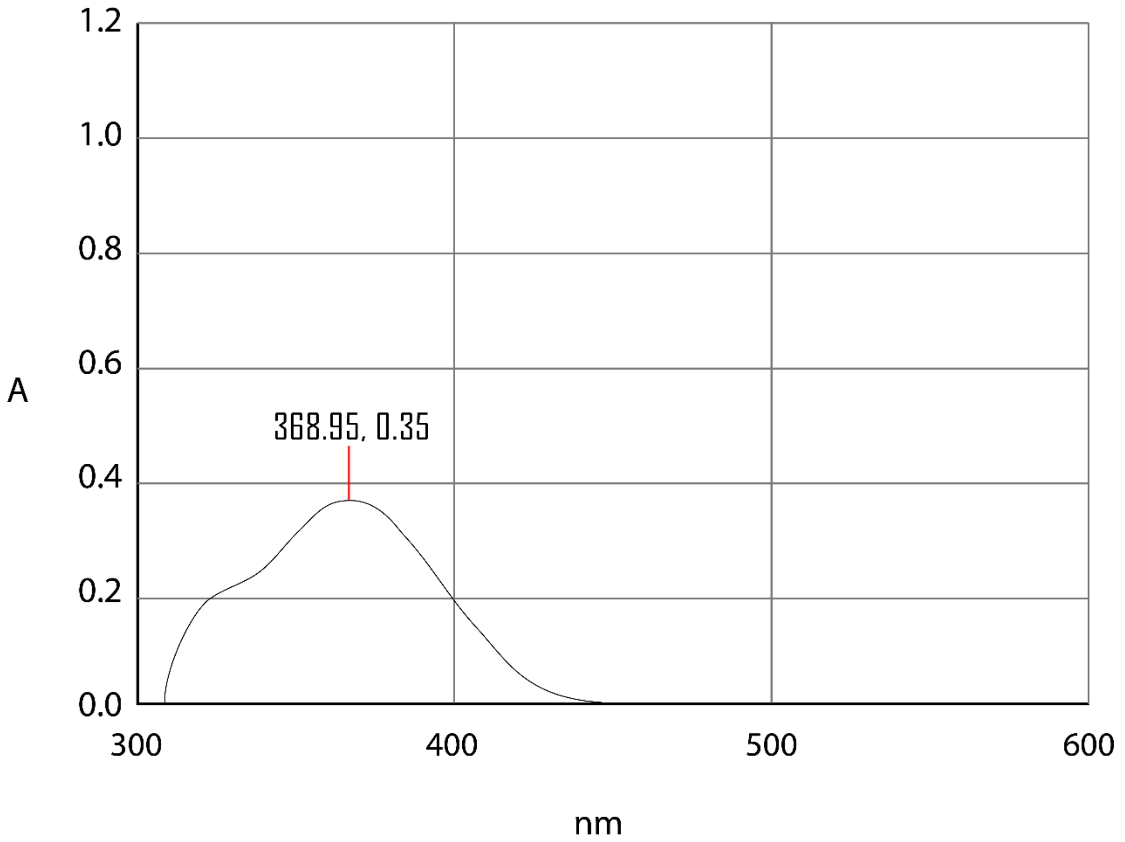

Figure S3. Absorption UV/Vis spectrum of ***E*-9** in $CHCl_3$ (with DMSO drops) at room temperature with concentration of 5X10^{-5} M.

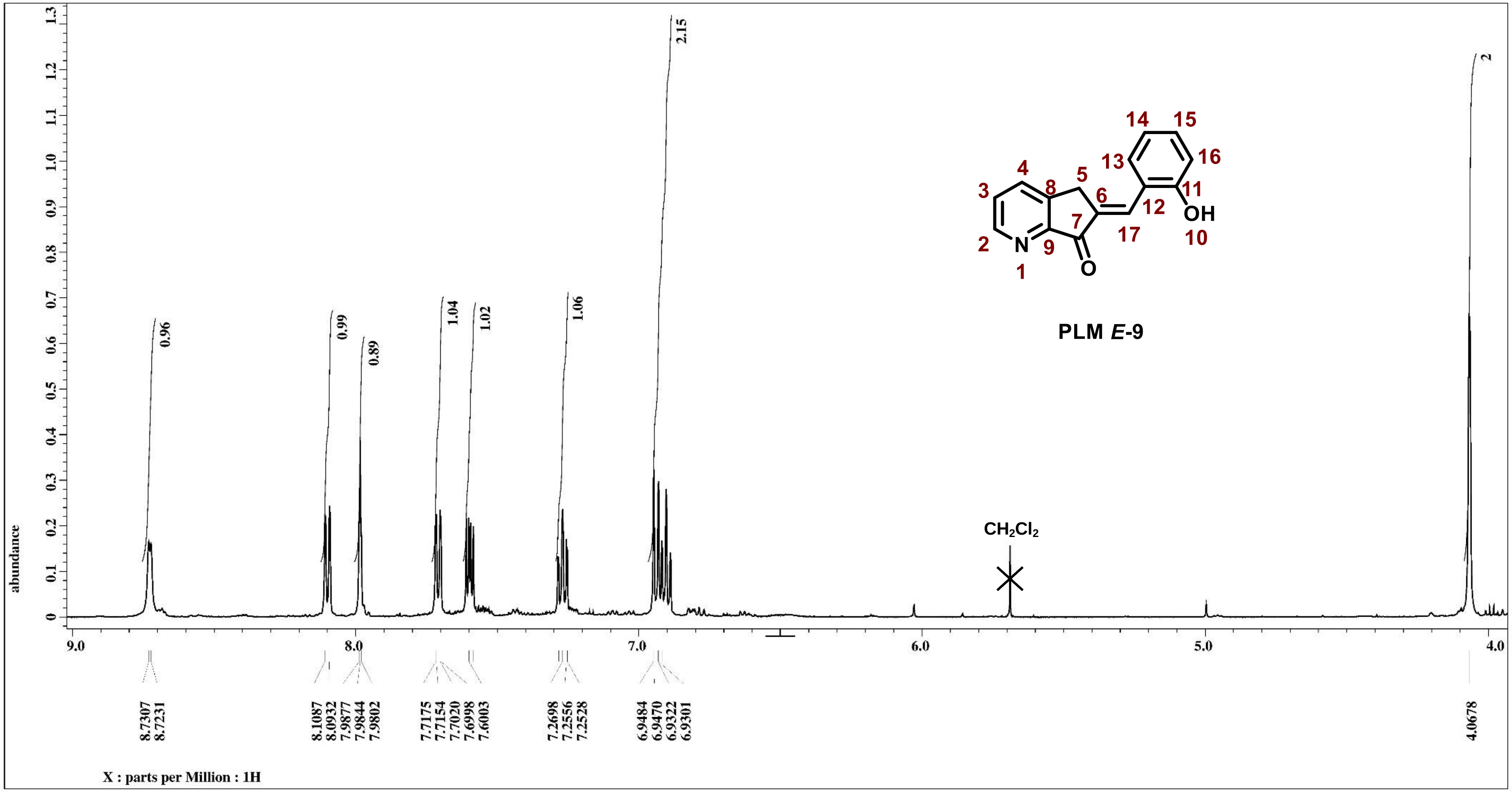

abundance
X : parts per Million : 1H
0.96
0.99
0.89
1.04
1.02
1.06
2.15
2
CH2Cl2
PLM E-9
OH
N
O
8.7307
8.7231
8.1087
8.0932
7.9877
7.9844
7.9802
7.7175
7.7154
7.7020
7.6998
7.6003
7.2698
7.2556
7.2528
6.9484
6.9470
6.9322
6.9301
4.0678

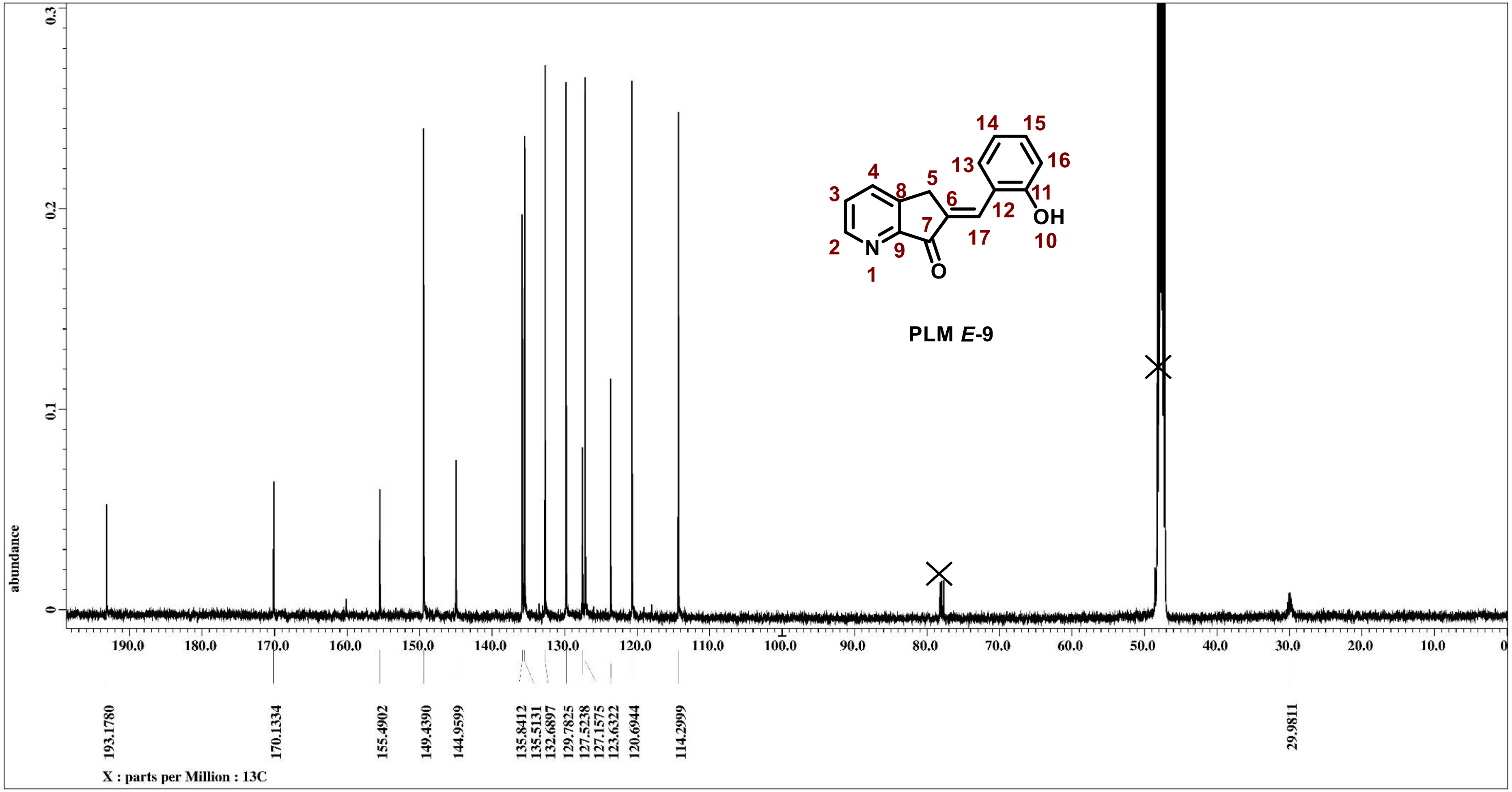
abundance
0.3
0.2
0.1
0
193.1780
170.1334
155.4902
149.4390
144.9599
135.8412
135.5131
132.6897
129.7825
127.5238
127.1575
123.6322
120.6944
114.2999
29.9811
X : parts per Million : 13C
14
15
16
13
4
5
3
8
6
11
12
OH
7
2
N
9
17
10
1
O

PLM *E*-9

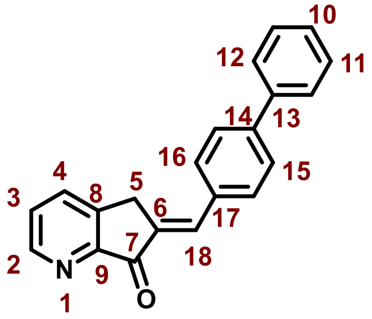

PLM *E*-10

(E)-6-([1,1'-biphenyl]-4-ylmethylene)-5,6-dihydro-7H-cyclopenta[b]pyridin-7-one (10):

^{1}H NMR ($CDCl_3$, 500 MHz): **H2** δ 8.83 (1H, d, *J* = 3.8 Hz), **H4** δ 8.00 (1H, d, *J* = 7.7 Hz), **H18** δ 7.86 (1H, s), **H16** δ 7.76 (2H, d, *J* = 8.1 Hz), **H15** δ 7.70 (2H, d, *J* = 8.1 Hz), **H12** δ 7.63 (2H, d, *J* = 7.7 Hz), **H3** δ 7.51 (1H, dd, *J* = 4.5, 7.7 Hz), **H11** δ 7.47 (2H, t, *J* = 7.7 Hz), **H10** δ 7.40 (1H, d, *J* = 1.2 Hz), **H5** δ 4.12 (2H, s).

^{13}C {^{1}H} NMR ($CDCl_3$, 125 MHz): δ **C7** 192.70, **C9** 155.03, **C2** 150.76, **C6** 144.37, **C13** 143.08, **C14** 140.00, **C18** 136.00, **C4** 135.14, **C17** 133.90, **C8** 132.35, **C16** 131.63, **C11** 129.08, **C10** 128.18, **C15** 127.79, **C3** 127.56, **C12** 127.20, **C5** 30.71.

ES-API *m/z*: M^+ 298.3.

Absorption coefficient in $CHCl_3$: 27000 cm^{-1}

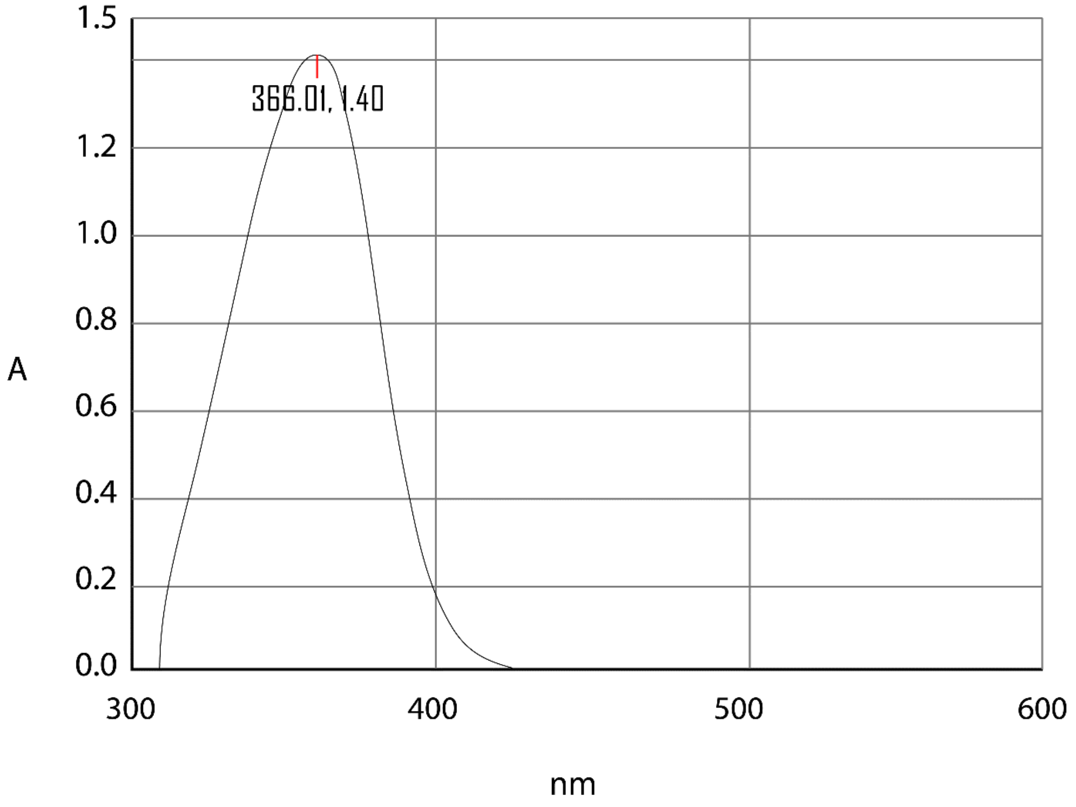

Figure S4. Absorption UV/Vis spectrum of ***E*-10** in $CHCl_3$ at room temperature with concentration of $5X10^{-5}$ M.

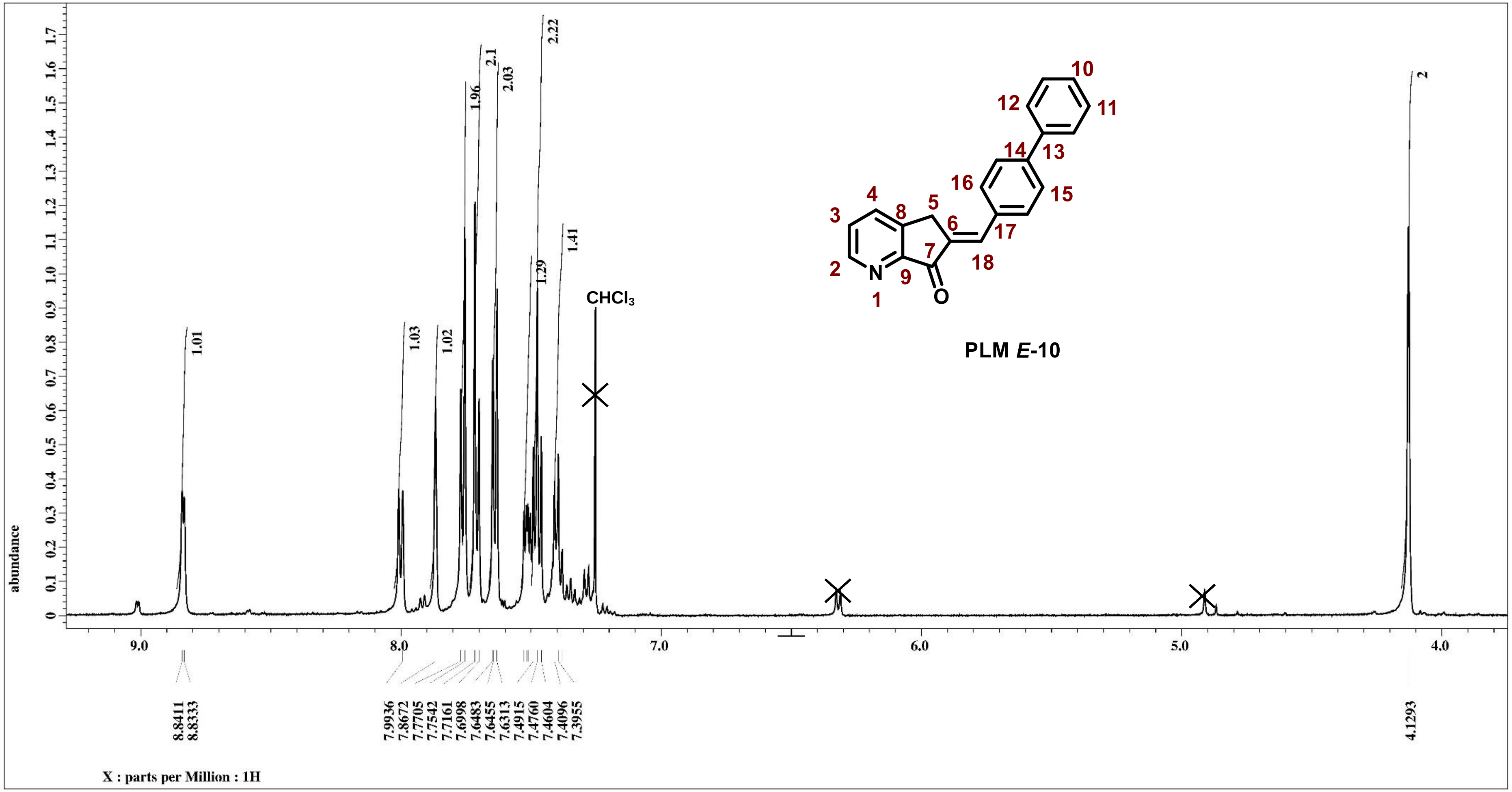
abundance
0
0.1
0.2
0.3
0.4
0.5
0.6
0.7
0.8
0.9
1.0
1.1
1.2
1.3
1.4
1.5
1.6
1.7
1.01
1.03
1.02
1.96
2.1
2.03
1.29
2.22
1.41
CHCl3
2
PLM E-10
N
O
1
2
3
4
5
6
7
8
9
10
11
12
13
14
15
16
17
18
9.0
8.0
7.0
6.0
5.0
4.0
8.8411
8.8333
7.9936
7.8672
7.7705
7.7542
7.7161
7.6998
7.6483
7.6455
7.6313
7.4915
7.4760
7.4604
7.4096
7.3955
4.1293
X : parts per Million : 1H

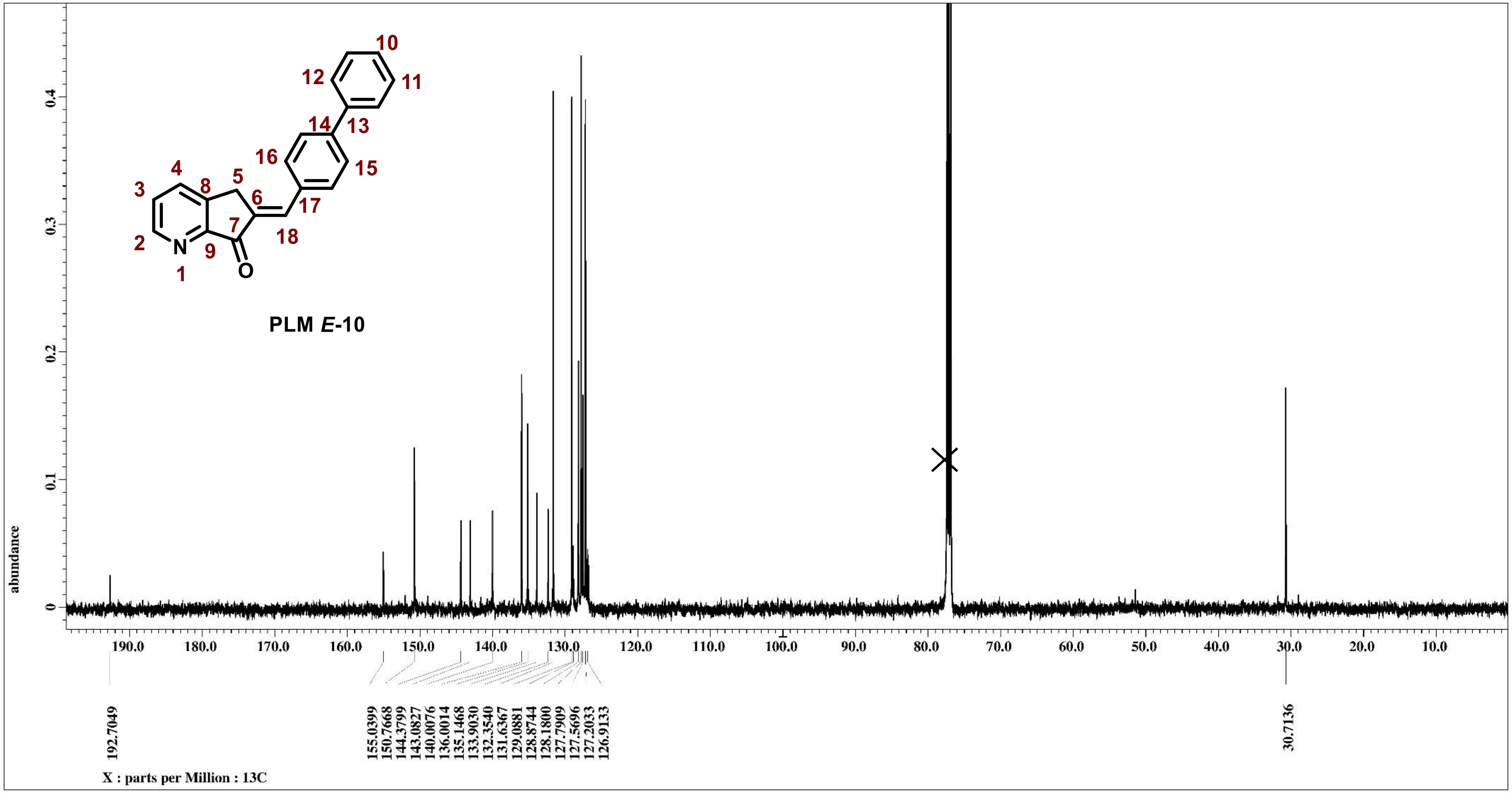
PLM E-10
abundance
X : parts per Million : 13C
192.7049
155.0399
150.7668
144.3799
143.0827
140.0076
136.0014
135.1468
133.9030
132.3540
131.6367
129.0881
128.8744
128.1800
127.7909
127.5696
127.2033
126.9133
30.7136

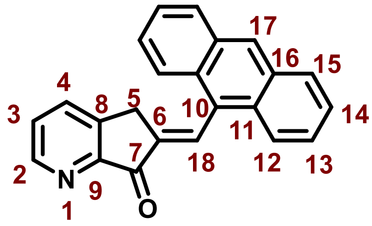

PLM *E*-11

(E)-6-(anthracen-9-ylmethylene)-5,6-dihydro-7H-cyclopenta[b]pyridin-7-one (11): ^{1}H NMR ($CDCl_3$, 500 MHz): **H2** δ 8.78 (1H, d, *J* = 4.2 Hz), **H18** δ 8.69 (1H, s), **H17** δ 8.46 (1H, s), **H12**, **H15** δ 8.01 (4H, m), **H4** δ 7.63 (1H, d, *J* = 7.7 Hz), **H13**, **H14** δ 7.47 (4H, m), **H3** δ 7.36 (1H, dd, *J* = 4.5, 7.7 Hz), **H5** δ 3.35 (2H, s).

^{13}C {^{1}H} NMR ($CDCl_3$, 125 MHz): δ **C7** 191.91, **C9** 155.39, **C2** 150.95, **C8** 144.88, **C6** 139.32, **C18** 134.92, **C4** 134.52, **C17** 131.33, **C10** 129.19, **C12** 129.14, **C11** 129.05, **C3** 128.24, **C16** 127.69, **C13** 126.57, **C14** 125.60, **C15** 125.50, **C5** 29.46.

ES-API *m/z*: M^{+} 322.4.

Absorption coefficient in $CHCl_3$: 1400 cm^{-1}

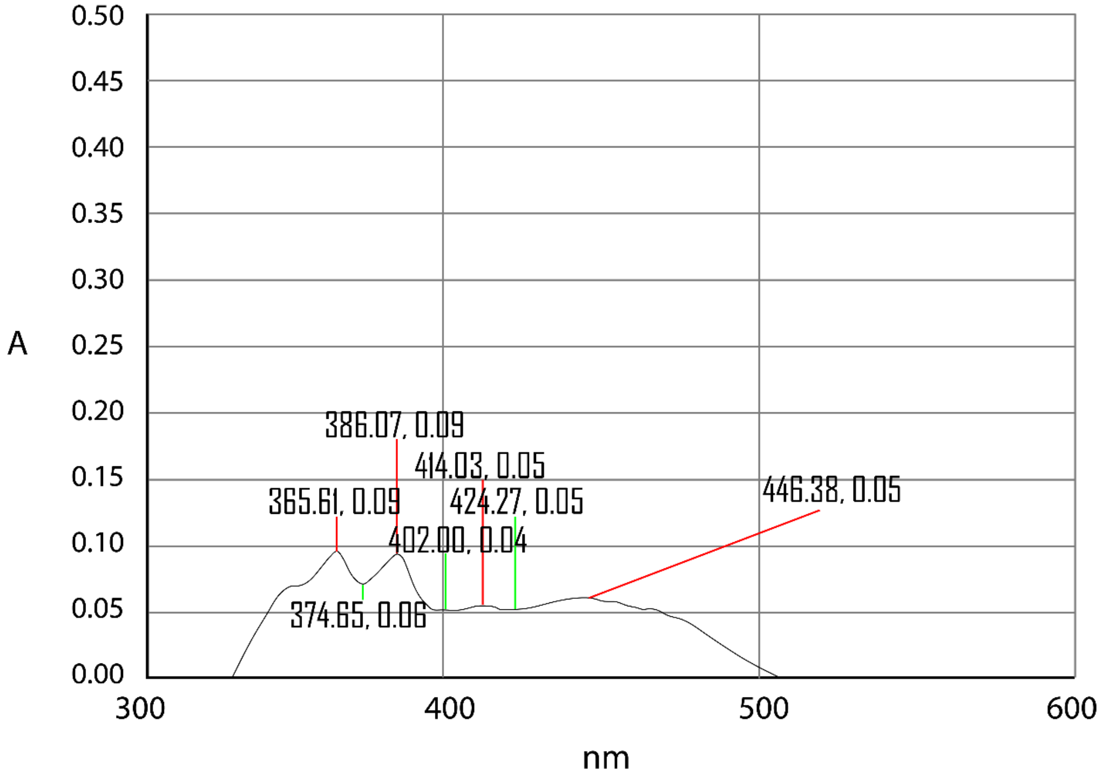

Figure S5. Absorption UV/Vis spectrum of ***E*-11** in $CHCl_3$ at room temperature with concentration of 5X10^{-5} M.

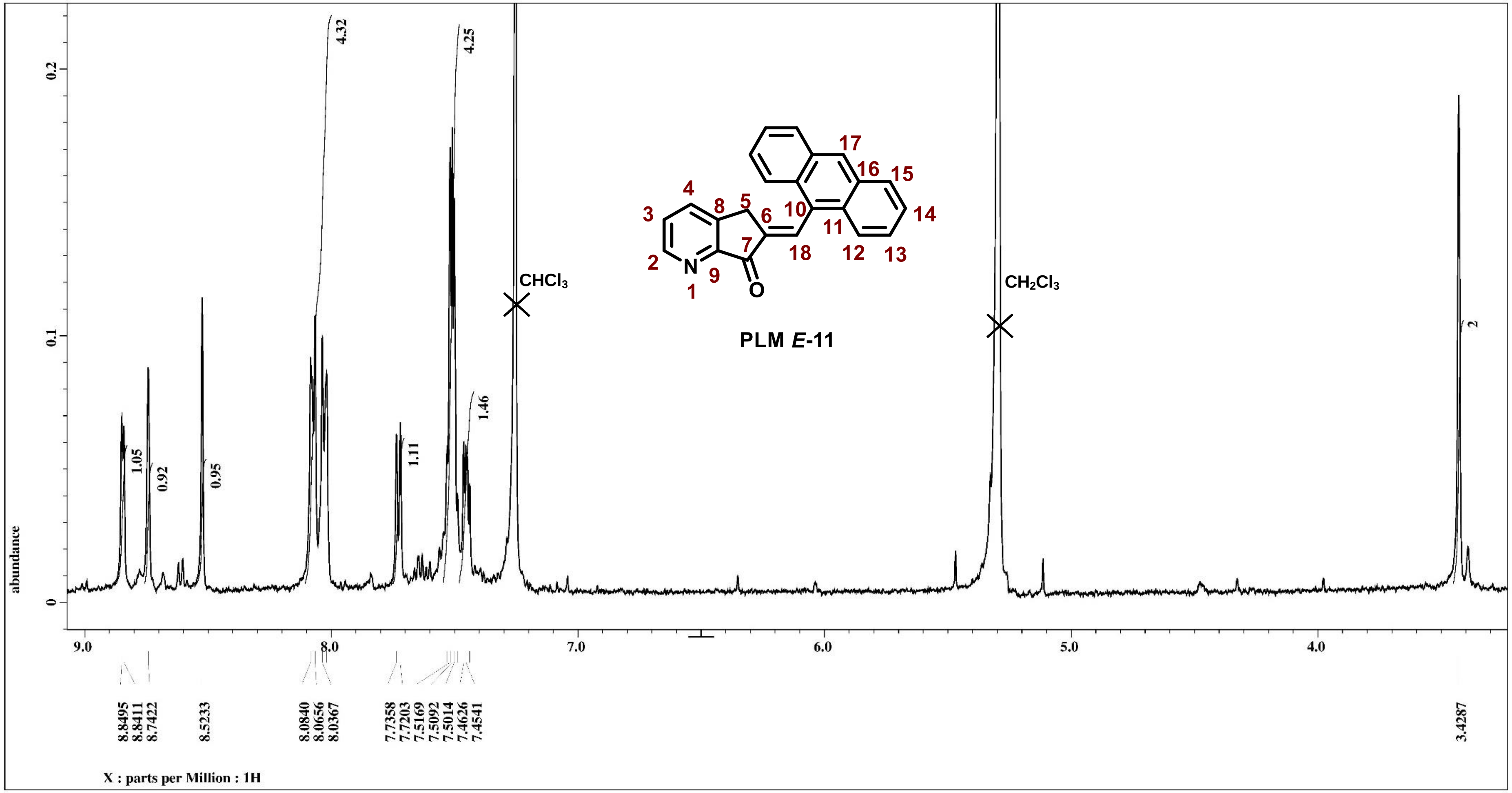

CHCl3
CH2Cl3
PLM E-11
abundance
X : parts per Million : 1H

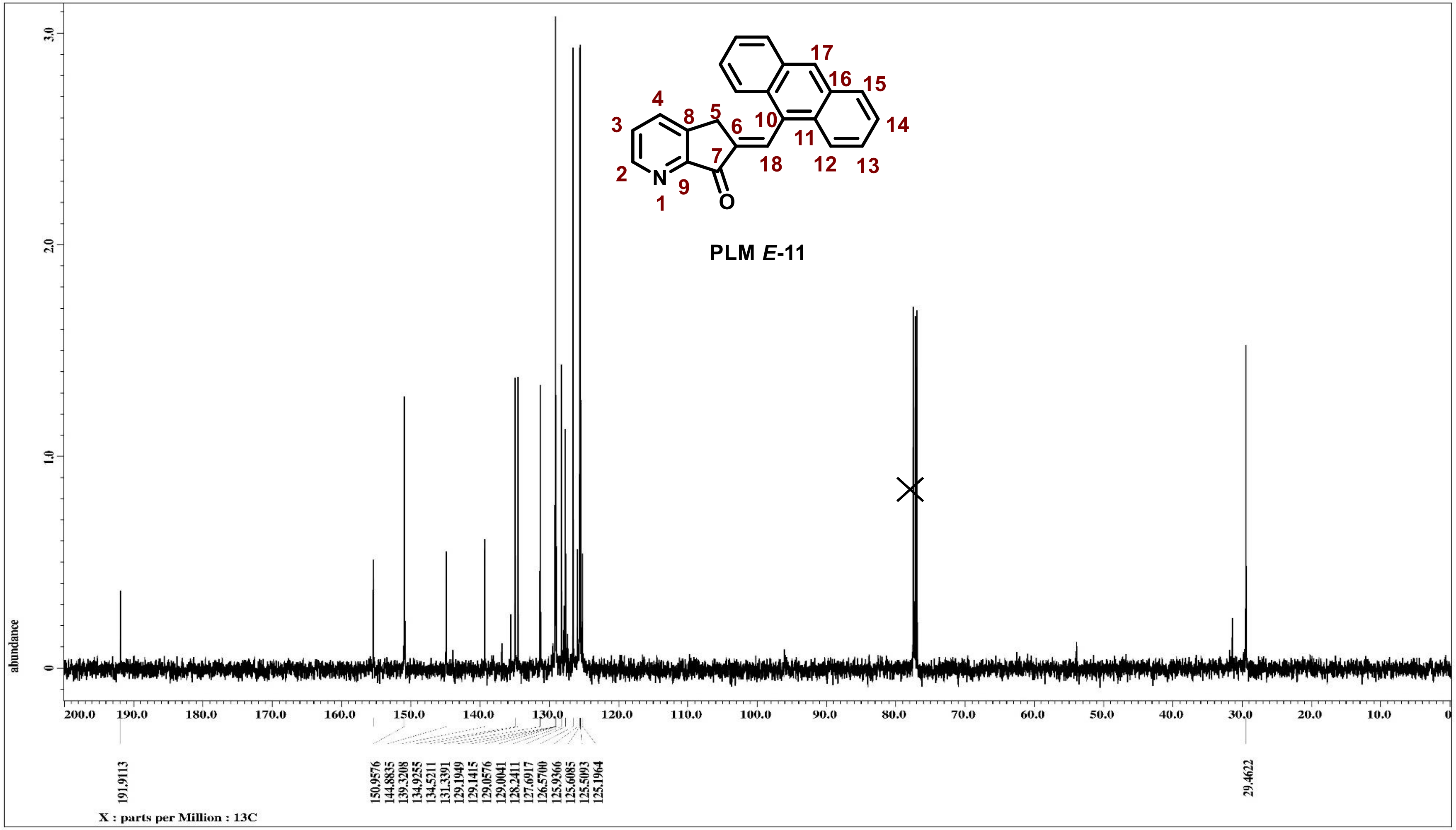

PLM E-11
abundance
X : parts per Million : 13C
191.9113
150.9576
144.8835
139.3208
134.9255
134.5211
131.3391
129.1949
129.1415
129.0576
129.0041
128.2411
127.6917
126.5700
125.9366
125.6085
125.5093
125.1964
29.4622

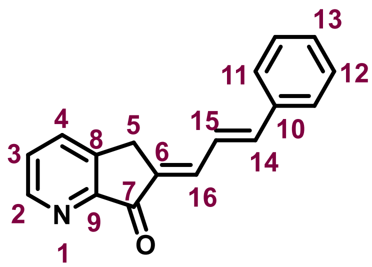

PLM *E*-12

(E)-6-((E)-3-phenylallylidene)-5,6-dihydro-7H-cyclopenta[b]pyridin-7-one (12):

^{1}H NMR ($CDCl_3$, 500 MHz): **H2** δ 8.77 (1H, d, *J* = 4.4 Hz), **H4** δ 7.91 (1H, d, *J* = 7.7 Hz), **H14, H11** δ 7.52 (3H, m), **H3** δ 7.43 (1H, dd, *J* = 4.7, 6.4 Hz), **H12** δ 7.36 (2H, td, *J* = 1.2, 7.8 Hz) **H13** δ 7.33 (2H, td, *J* = 1.2, 7.0 Hz), **H16, H15** δ 7.06 (2H, m), **H5** δ 3.86 (2H, s).

^{13}C {^{1}H} NMR ($CDCl_3$, 125 MHz): δ **C7** 192.40, **C9** 156.32, **C2** 150.70, **C8** 143.72, **C16** 143.36, **C6** 136.14, **C14** 135.64, **C4** 134.91, **C10** 133.73, **C13** 129.69, **C12** 129.07, **C11** 127.58, **C3** 127.31, **C15** 123.70, **C5** 28.47.

ES-API *m/z*: M^+ 248.2.

Absorption coefficient in $CHCl_3$: 17000 cm^{-1}

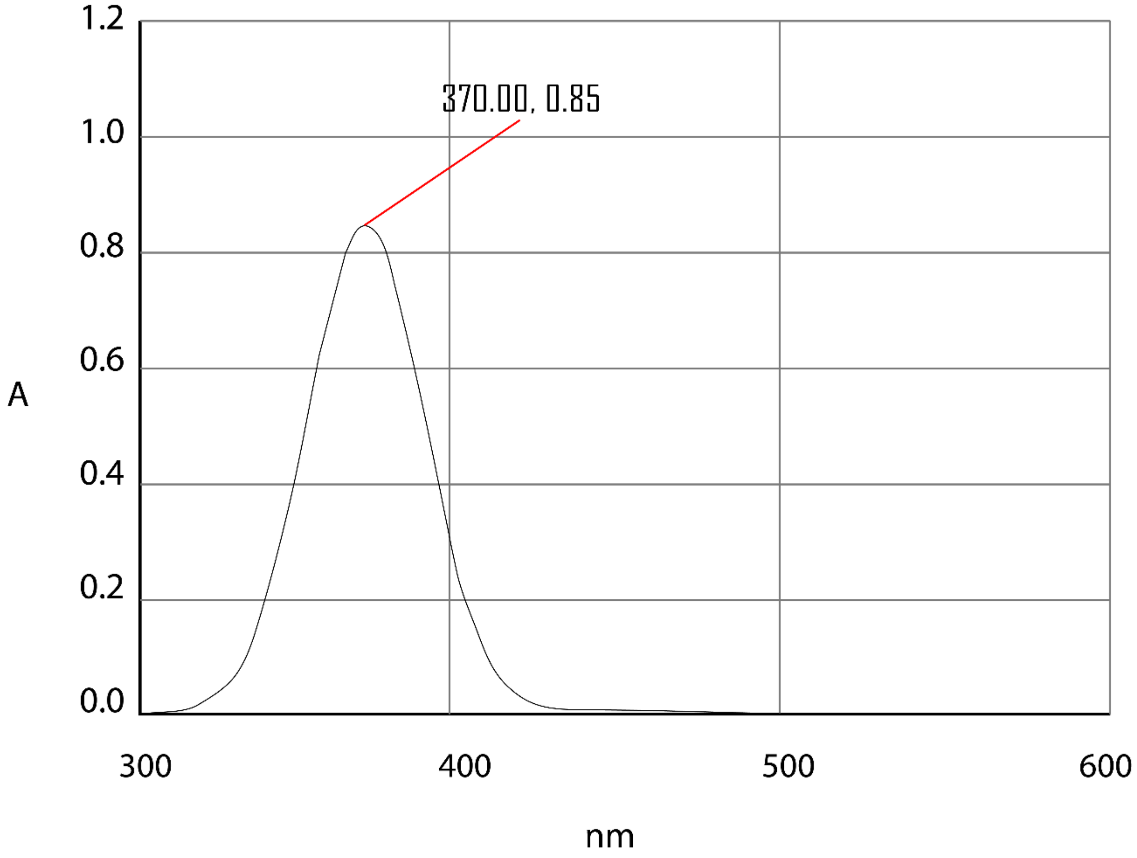

Figure S6. Absorption UV/Vis spectrum of ***E*-12** in $CHCl_3$ at room temperature with concentration of 5X10^{-5} M.

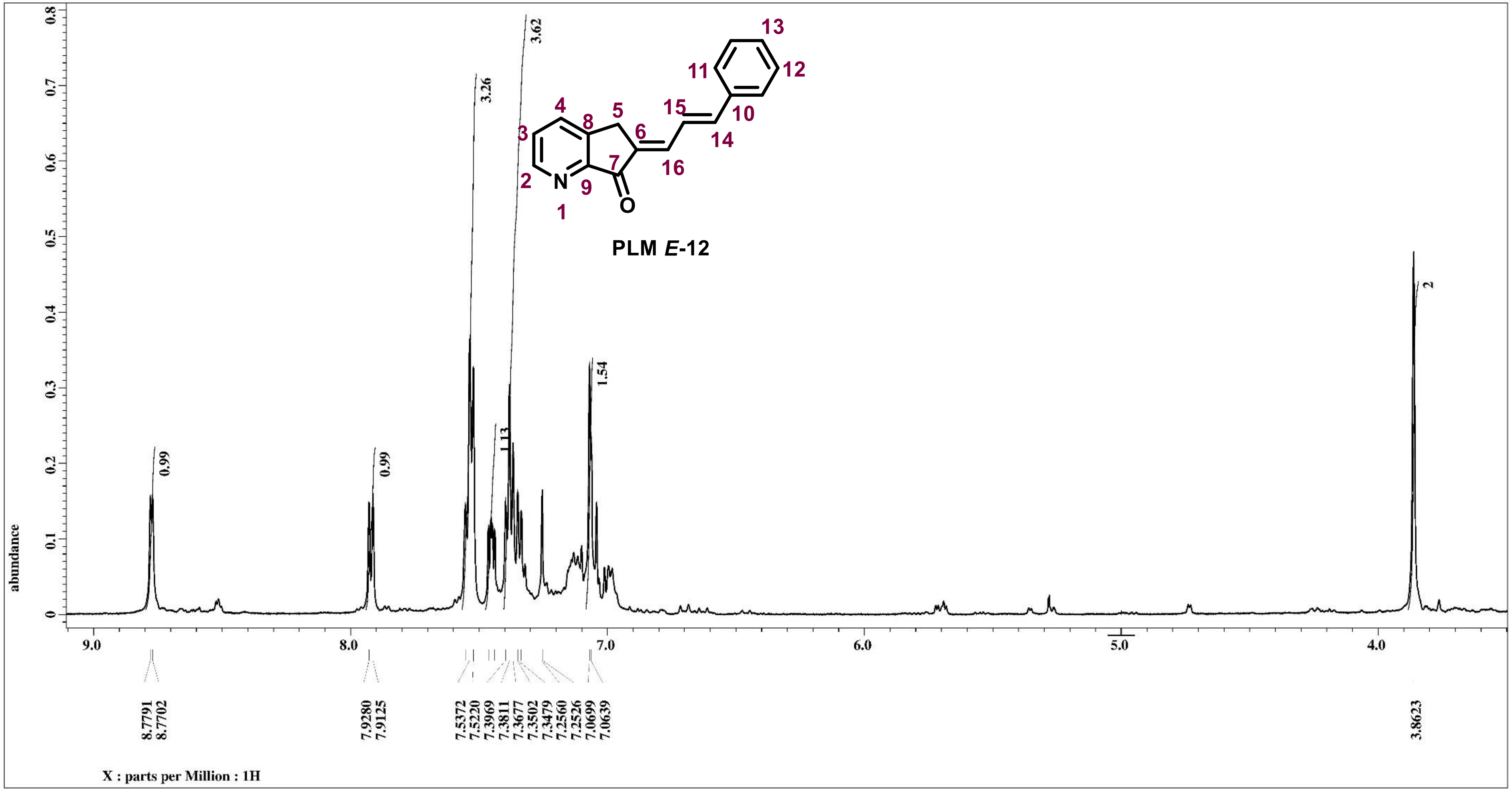
PLM E-12
abundance
X : parts per Million : 1H
0.99
0.99
3.26
3.62
1.13
1.54
2
8.7791
8.7702
7.9280
7.9125
7.5372
7.5220
7.3969
7.3811
7.3677
7.3502
7.3479
7.2560
7.2526
7.0699
7.0639
3.8623

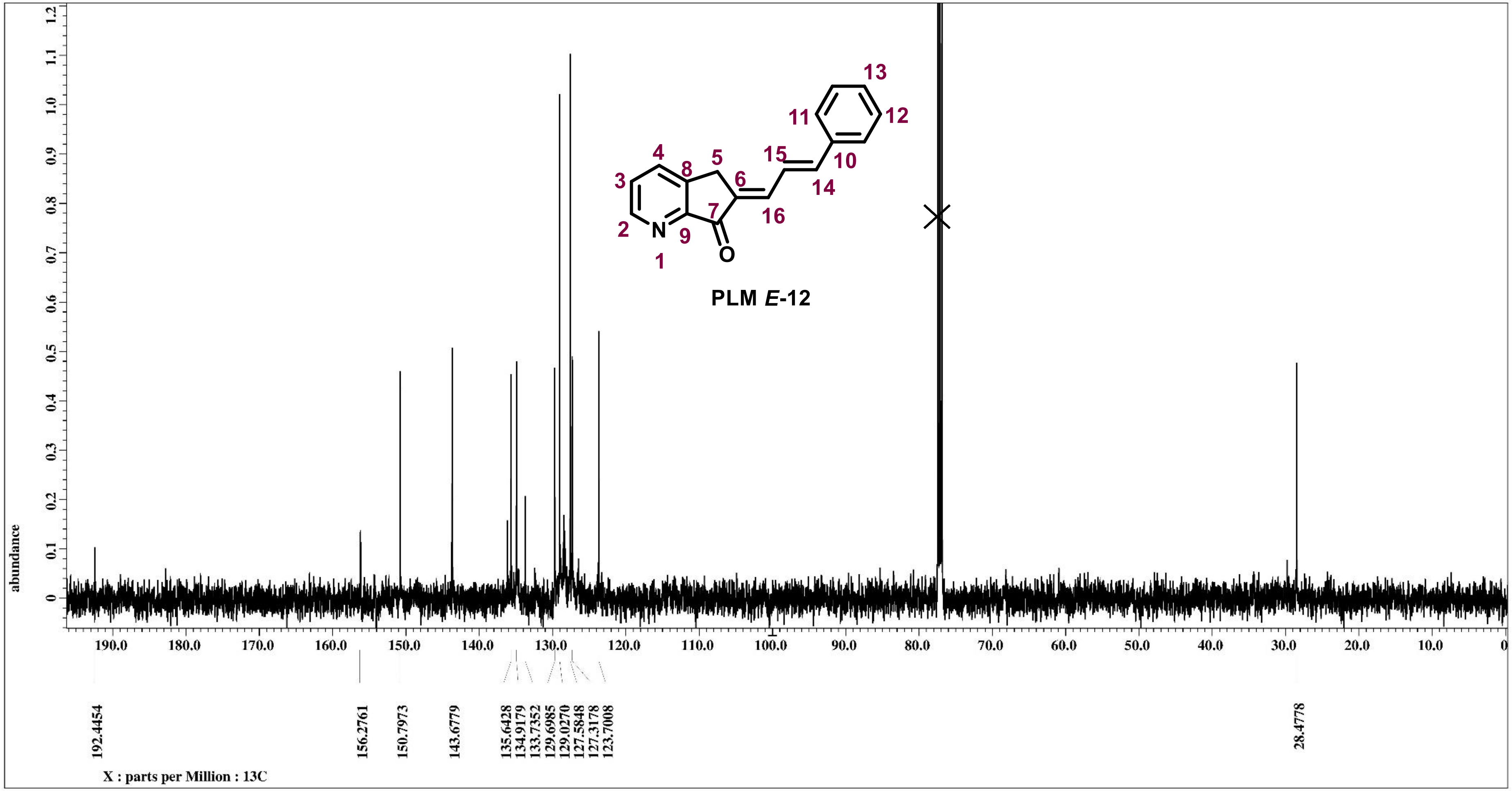

PLM E-12
abundance
X : parts per Million : 13C
192.4454
156.2761
150.7973
143.6779
135.6428
134.9179
133.7352
129.6985
129.0270
127.5848
127.3178
123.7008
28.4778

Irradiation response UV/vis spectrum

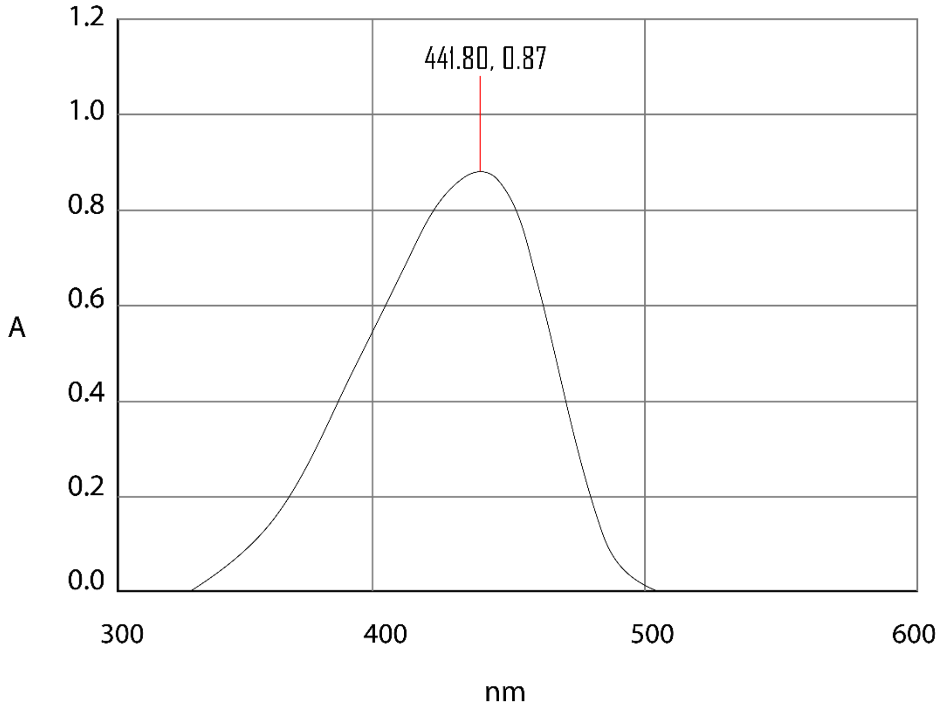

Figure S7. Absorption UV/Vis spectrum of ***E*-7** after irradiation with light of 400 nm for 7 min, in $CHCl_3$ at room temperature with concentration of $5X10^{-5}$ M. (Possible ***Z*-7** absorption spectrum)

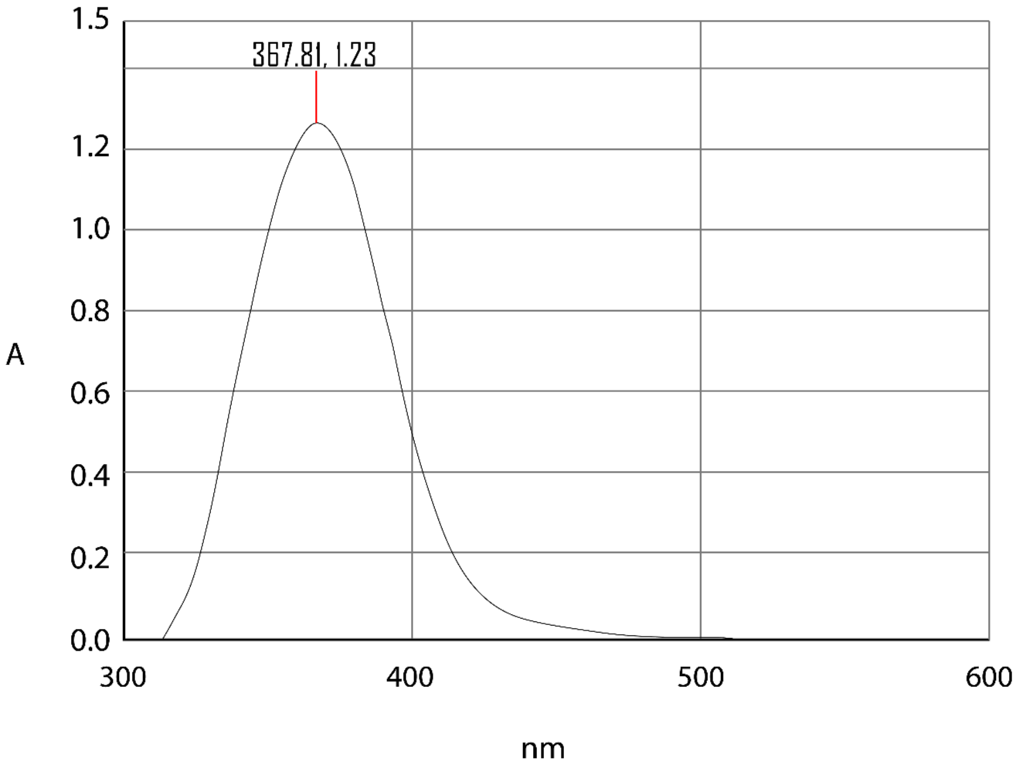

Figure S8. Absorption UV/Vis spectrum of ***E*-8** after irradiation with light of 400 nm for 7 min, in $CHCl_3$ at room temperature with concentration of $5X10^{-5}$ M. (Possible ***Z*-8** absorption spectrum)

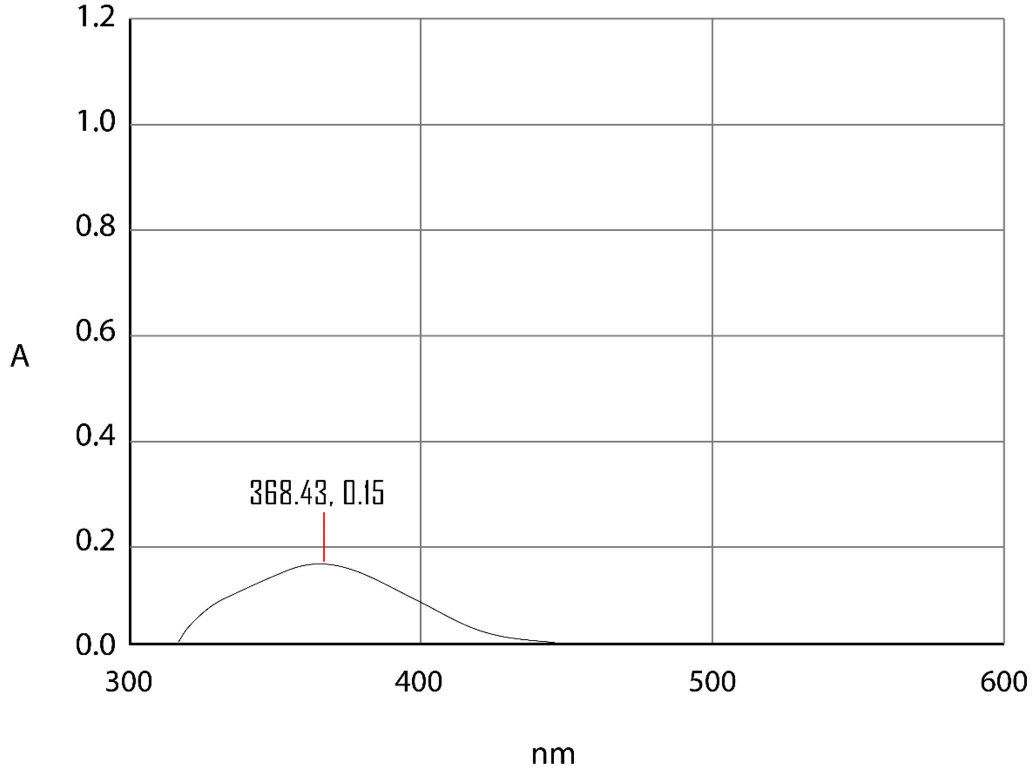

Figure S9. Absorption UV/Vis spectrum of ***E*-9** after irradiation with light of 400 nm for 7 min, in $CHCl_3$ at room temperature with concentration of 5X10^{-5} M. (Possible ***Z*-9** absorption spectrum)

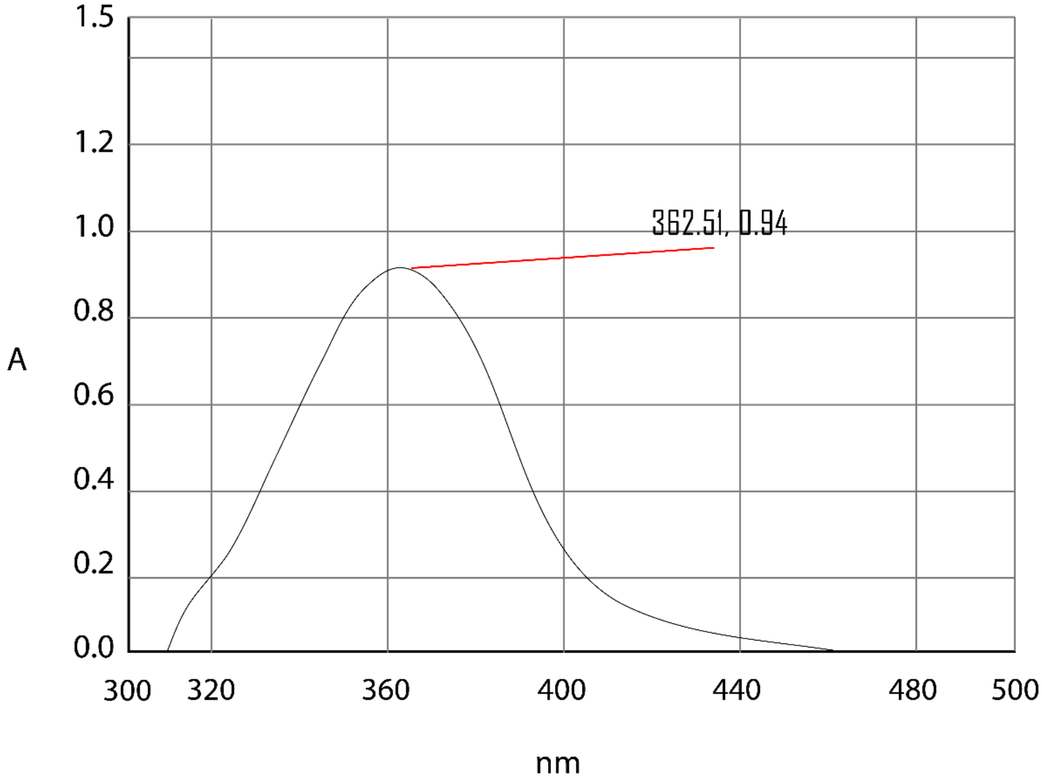

Figure S10. Absorption UV/Vis spectrum of ***E*-10** after irradiation with light of 400 nm for 7 min, in $CHCl_3$ at room temperature with concentration of 5X10^{-5} M. (Possible ***Z*-10** absorption spectrum)

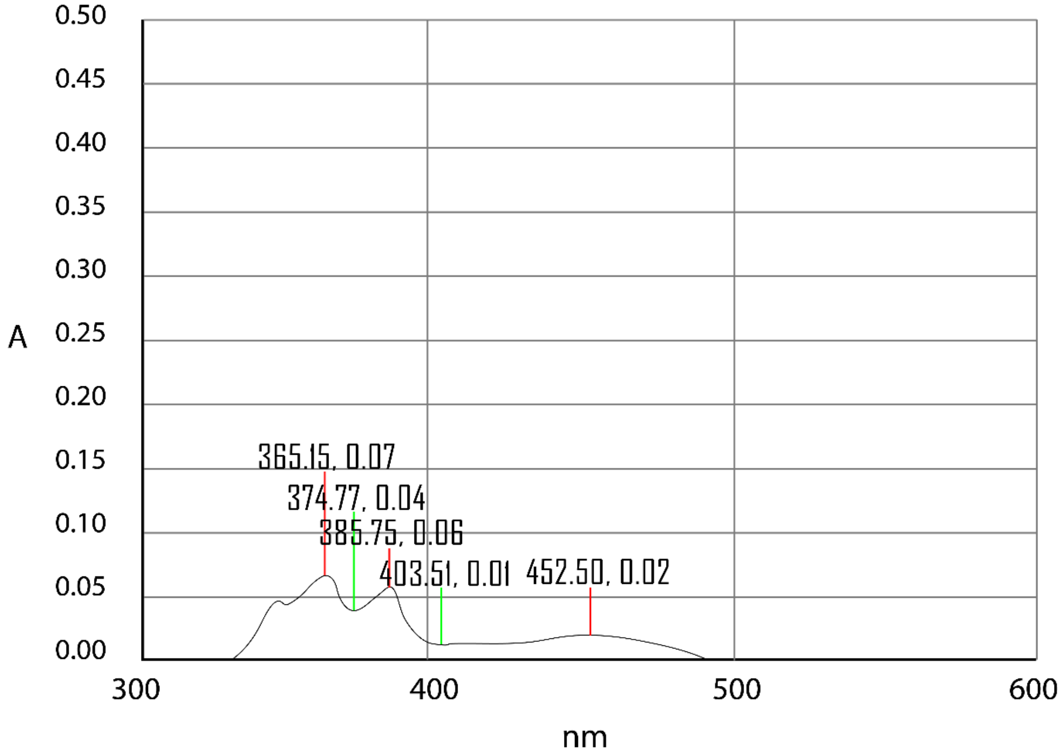

Figure S11. Absorption UV/Vis spectrum of ***E*-11** after irradiation with light of 445 nm for 7 min, in $CHCl_3$ at room temperature with concentration of 3X10^{-5} M. (Possible ***Z*-11** absorption spectrum)

We did not print the spectrum of ***E*-12** after de irradiation because we did not observe any signal in the spectrum. Therefore, the irradiation of ***E*-12** was studied by NMR ^{1}H, to understand why we did not observe changes in the UV/Vis spectrum.

Titration procedure

For the titration protocol, apart of MLG solutions in deuterated chloroform were prepared. These solutions were prepared with a concentration range of 0.003-0.005 M using 0.3mL of $CDCl_3$. MLGs **7** and **9** were dissolved in 0.1 mL of DMSO d_6 and measured using 0.3mL of $CDCl_3$. The solutions of pTsOH were prepared using two different concentrations, 0.05 M and 0.5M in 2.5mL of $CDCl_3$. Addition of pTsOH solution was performed using a micropipette and immediately recorded in a 500MHz NMR spectrophotometer and/or in 200 MHz NMR spectrophotometer.

Table S1. Calculated protonation constants K

MLG	Macomber[a]	Local analysis[b]	Thordarson[c]
**7**	122	182	109
**8**	54	86	20
**9**	71	31	21
**10**	940	914	1328
**11**	1518	1379	1210
**12**	248	178	120

[a]R. S. Macomber, *J. Chem. Educ.*, 1992, **69**, 375–378.

[b]P. Thordarson, *Chem. Soc. Rev.*, 2011, **40**, 1305–1323.

[c]D. B. Hibbert, P. Thordarson, *Chem. Commun.*, 2016, **52**, 12792–12805.

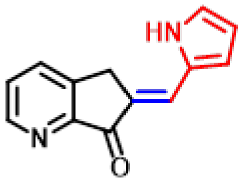

7

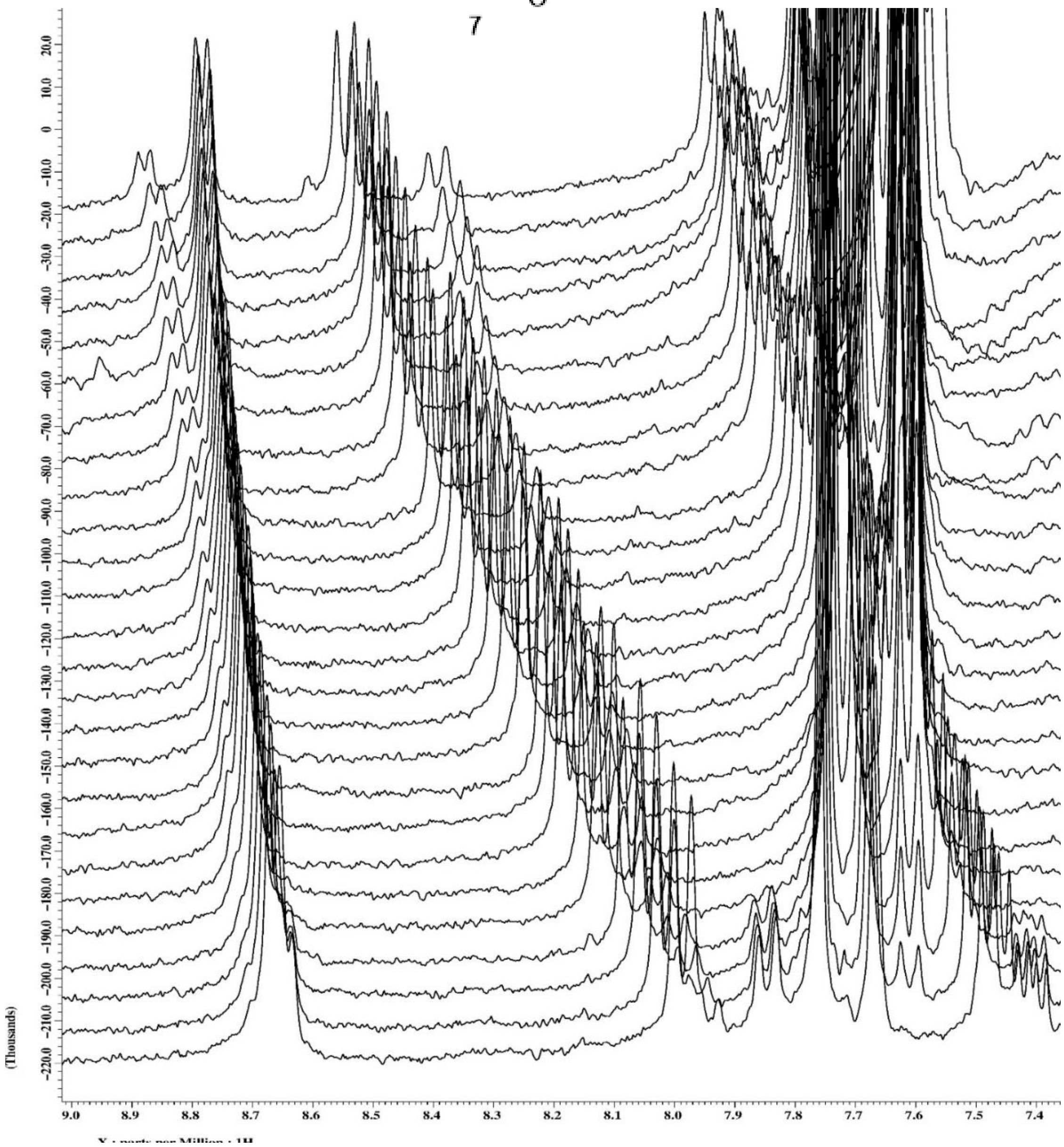

Figure S12. MLG **7** titration with pTsOH, recorded by NMR at 270 MHz in $CDCl_3$ at room temperature.

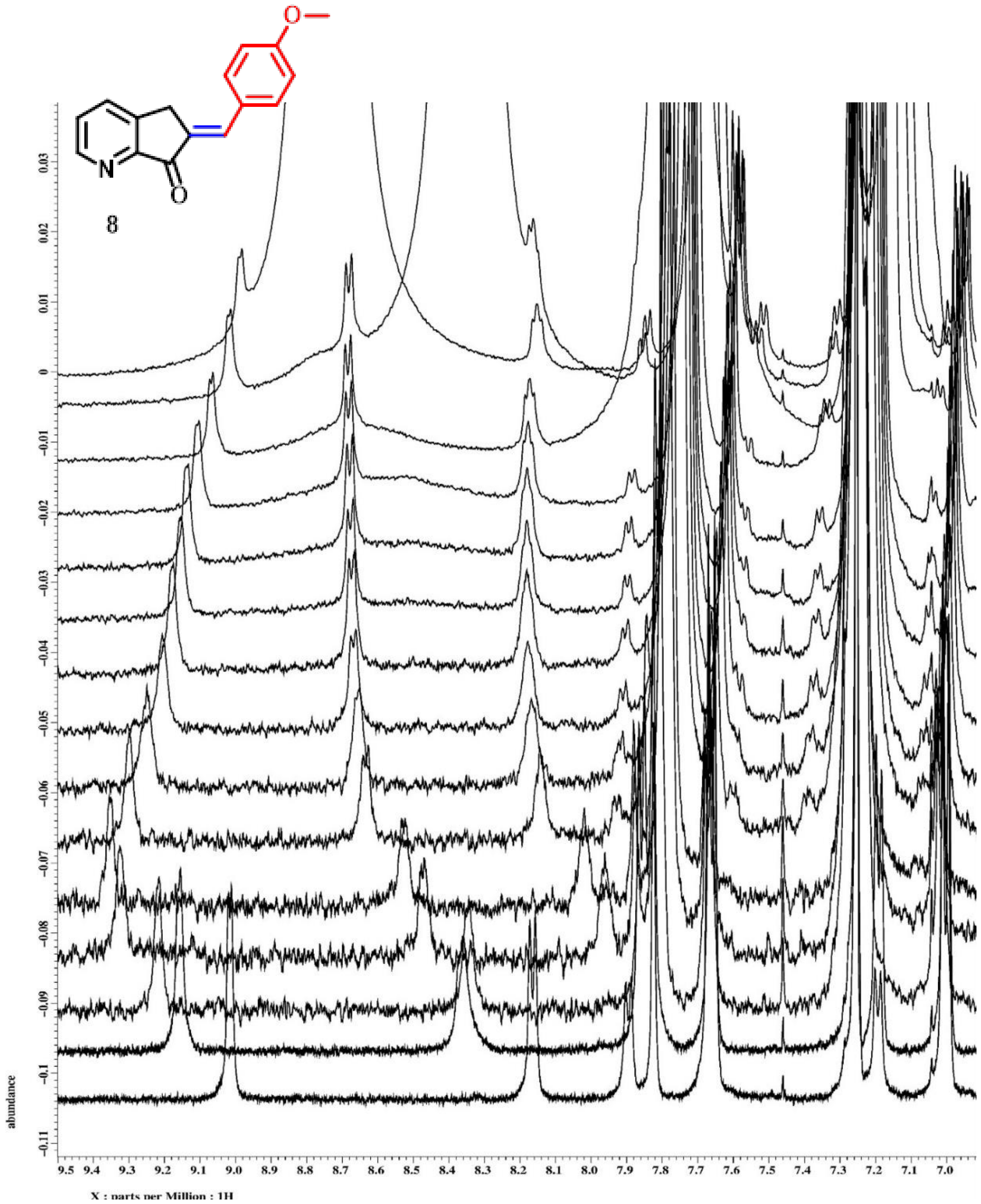

Figure S13. MLG **8** titration with pTsOH, recorded by NMR at 500 MHz in $CDCl_3$ at room temperature.

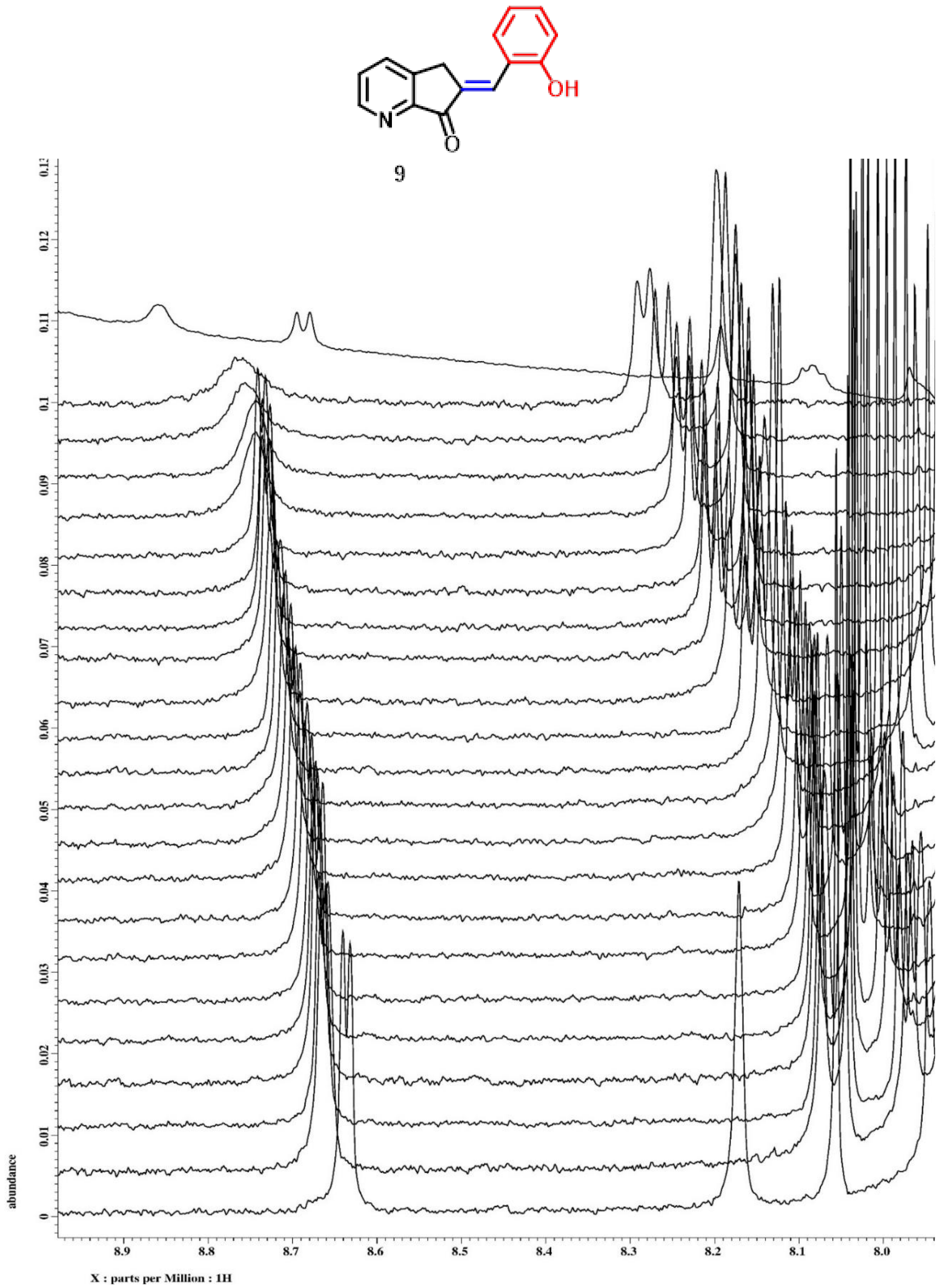

Figure S14. MLG **9** titration with pTsOH, recorded by NMR at 500 MHz in $CDCl_3$ at room temperature.

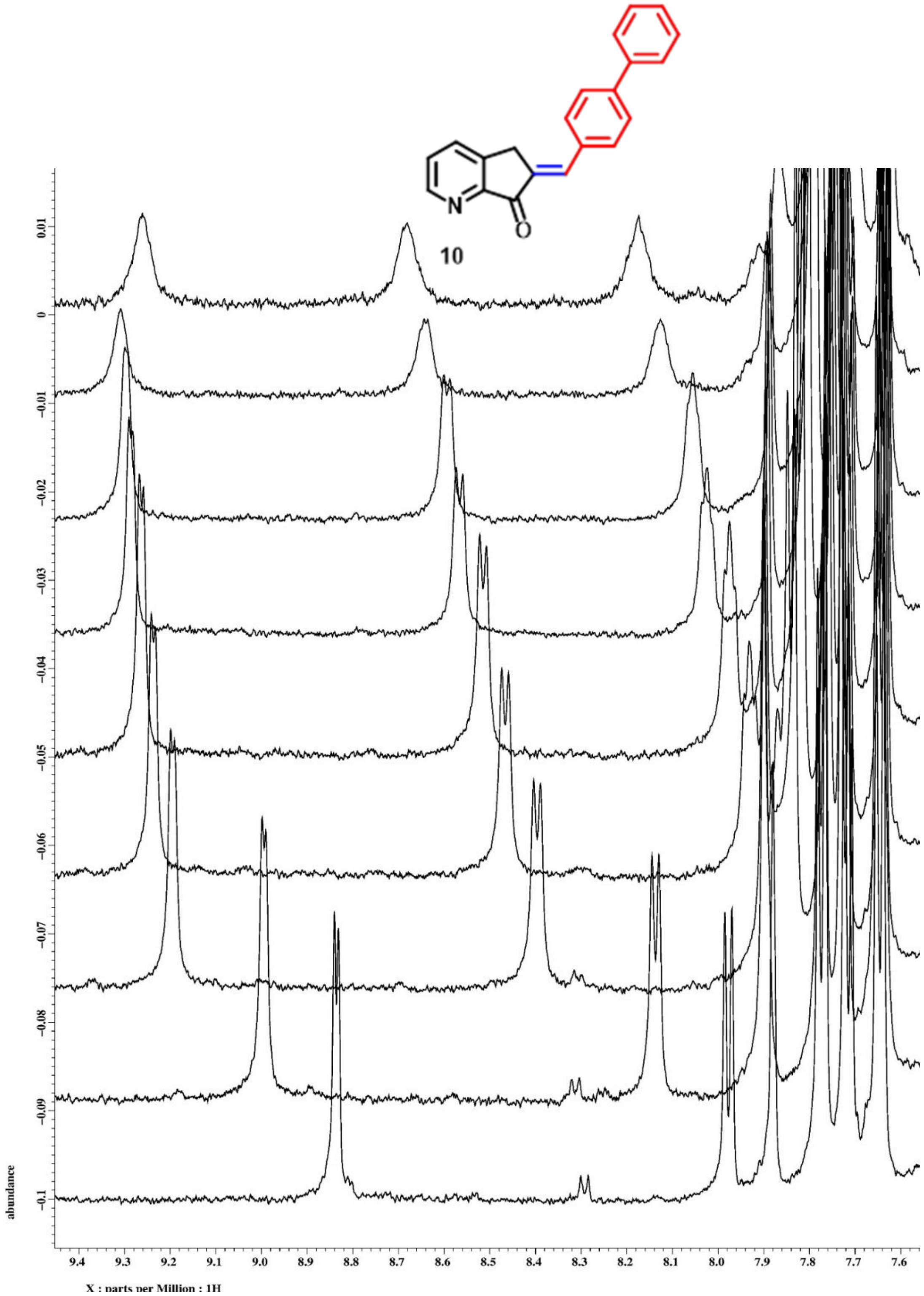

Figure S15. MLG **10** titration with pTsOH, recorded by NMR at 500 MHz in $CDCl_3$ at room temperature.

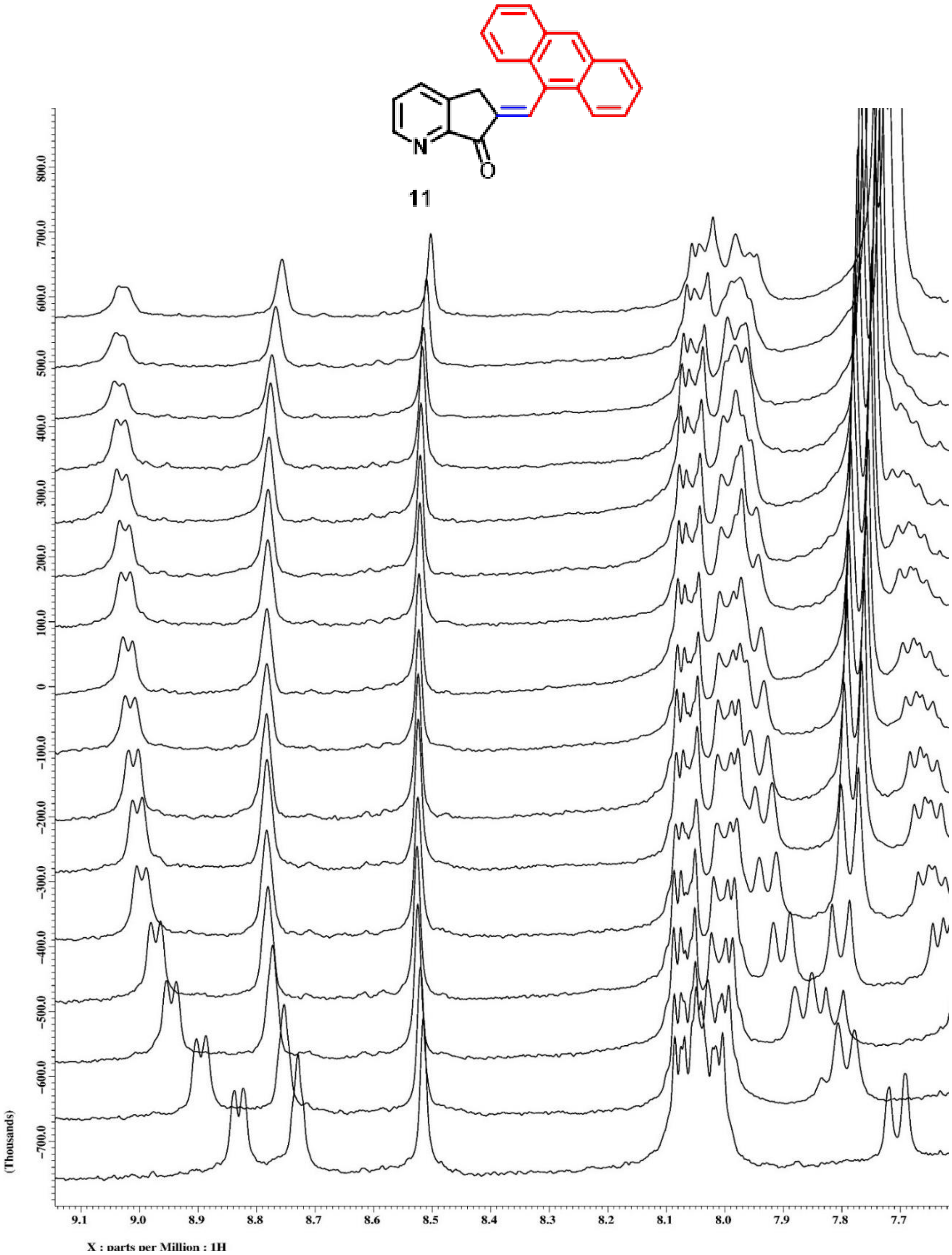

Figure S16. MLG **11** titration with pTsOH, recorded by NMR at 270 MHz in $CDCl_3$ at room temperature.

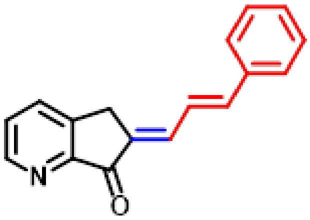

12

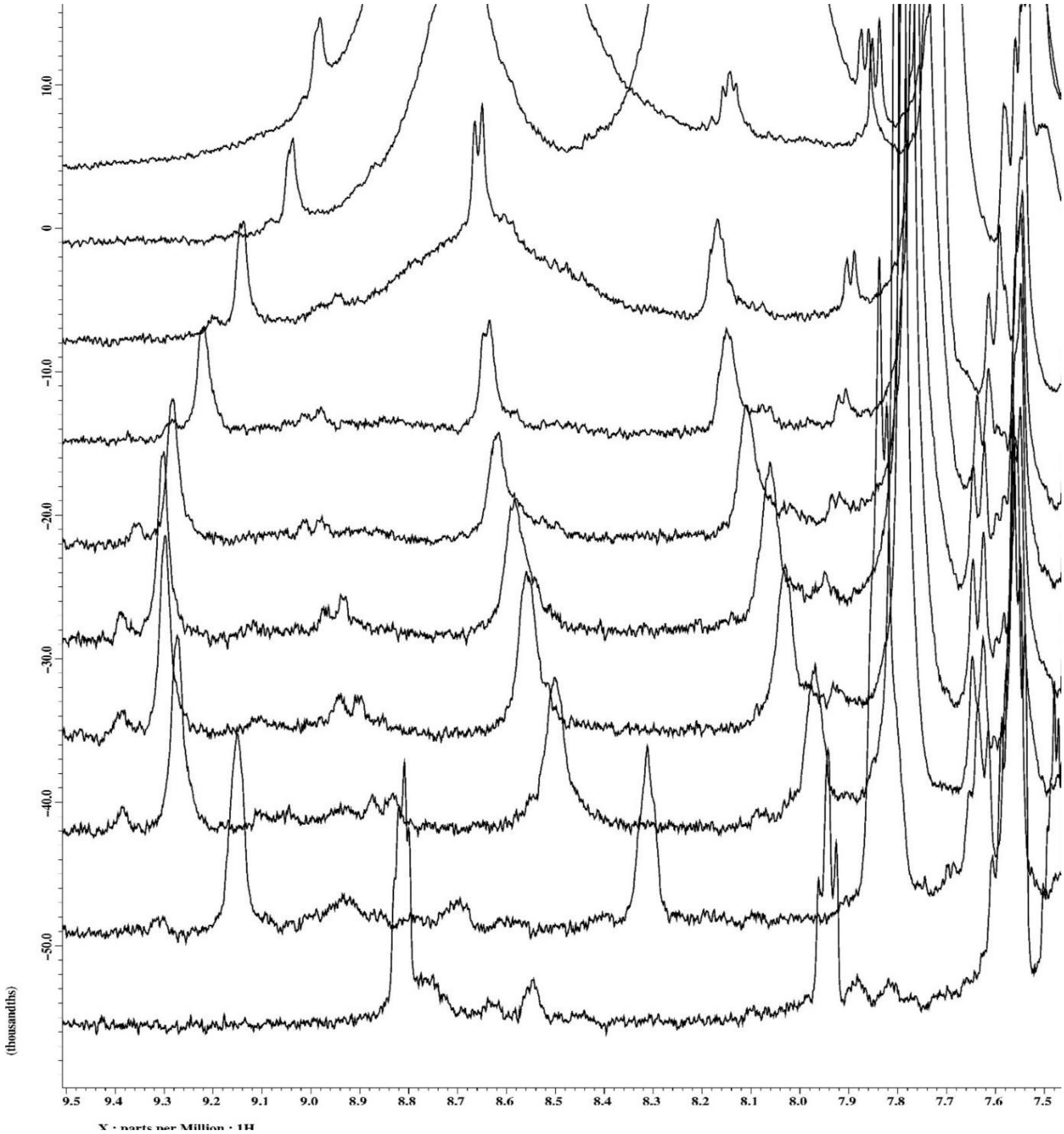

Figure S17. MLG **12** titration with pTsOH, recorded by NMR at 500 MHz in $CDCl_3$ at room temperature.

NMR nOe

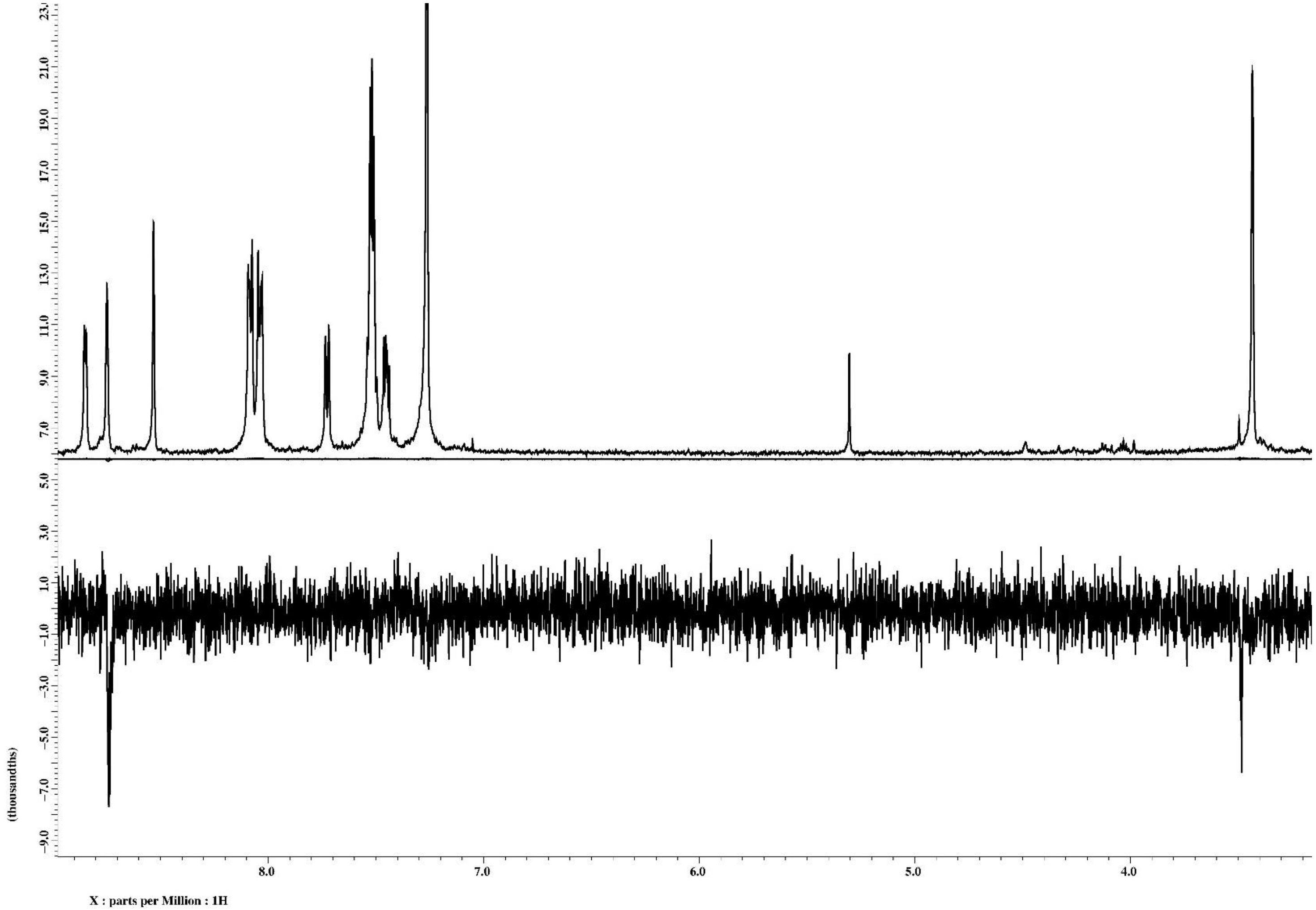

Figure S18. NMR nOe-dpfgse spectra of MLG ***E*-11** with the offset 8.73ppm recorded at 500 MHz in $CDCl_3$ at room temperature.

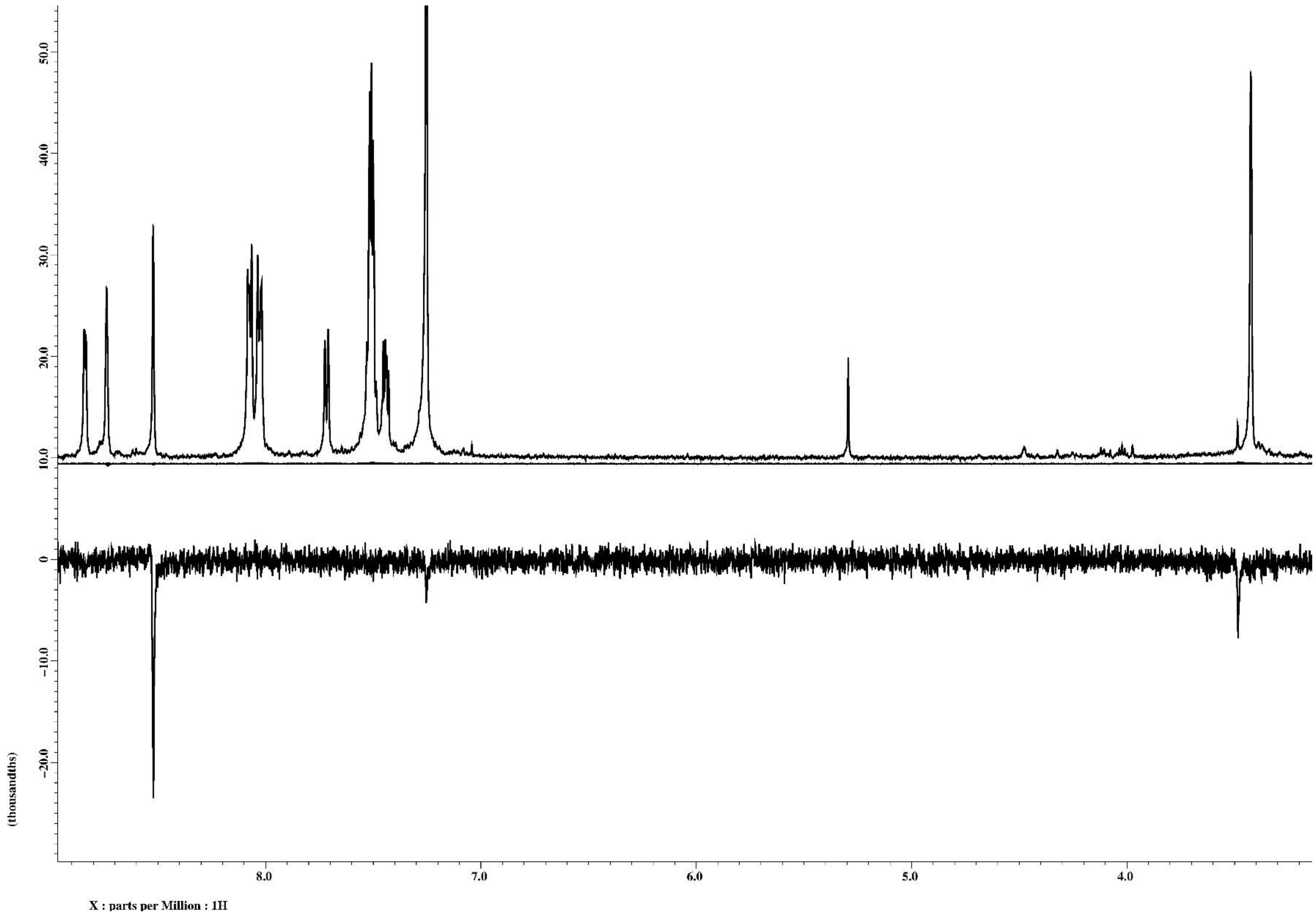

Figure S19. NMR nOe-dpfgse spectra of MLG ***E*-11** with the offset 8.52ppm recorded at 500 MHz in $CDCl_3$ at room temperature.

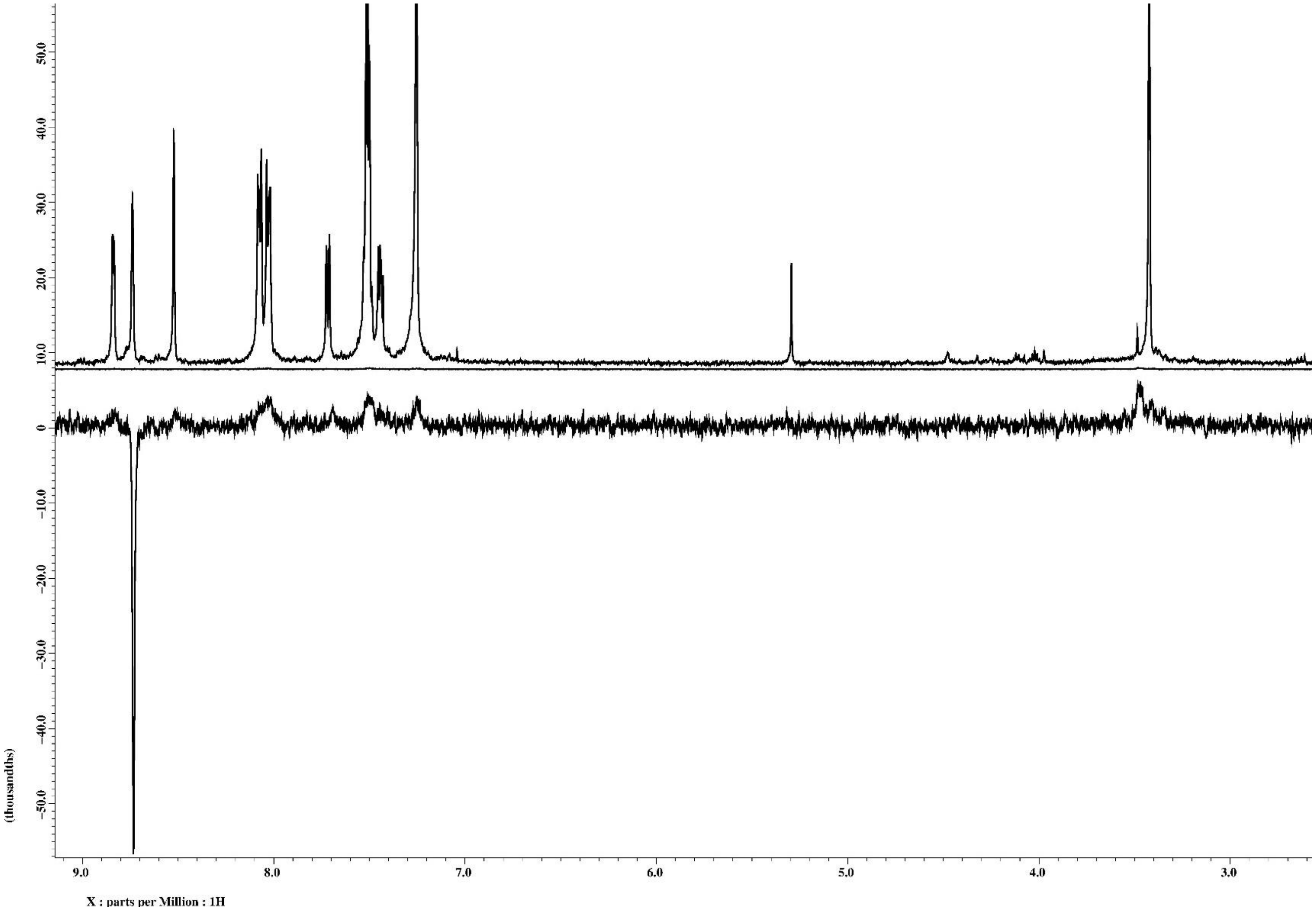

Figure S20. NMR nOe-dpfgse spectra of MLG ***E*-11** with the offset 8.52ppm recorded at 500 MHz in $CDCl_3$ at room temperature.

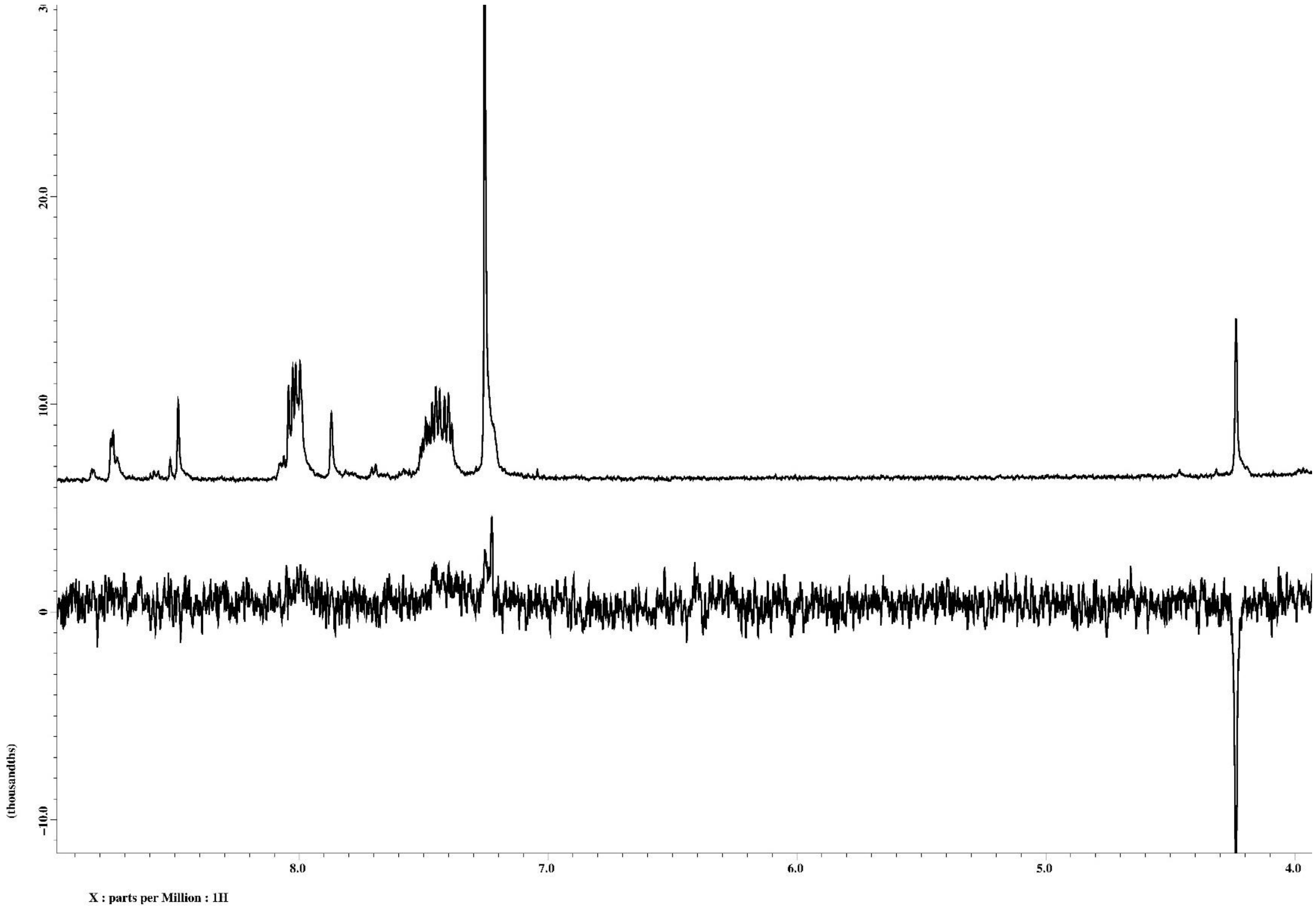

Figure S21. NMR nOe-dpfgse spectra of MLG ***Z*-12** with the offset 4.23ppm recorded at 500 MHz in $CDCl_3$ at room temperature.

Experimental determination of MLG operation

MLG operation process was studied using 0.002M solutions in $CDCl_3$ (for MLG **7** and **9** it was necessary to add 0.05mL of DMSO d6). In several experiments, the solutions were prepared using 0.0015M of each MLG in $CDCl_3$:DMSO d_6 (3:0.2 volume relation). pTsOH was added to the same titration solutions. Irradiation of the solutions in the NMR tube was performed using an Aldrich® Micro Photochemical Reactor; it is important to highlight that the NMR tube had intimate contact with the LED. To support the NMR tube firmly, aluminum cans were used as shown in image S18. Two photoreactors were set in the experiments, one with LEDs of 435-445nm wavelength and other with LEDs of 400-410nm wavelength.

All spectra were recorded with a 500MHz NMR spectrophotometer at room temperature.

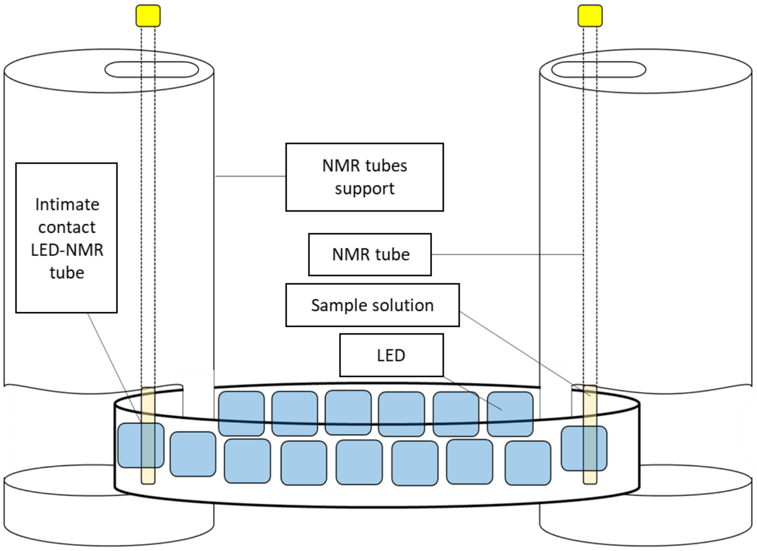

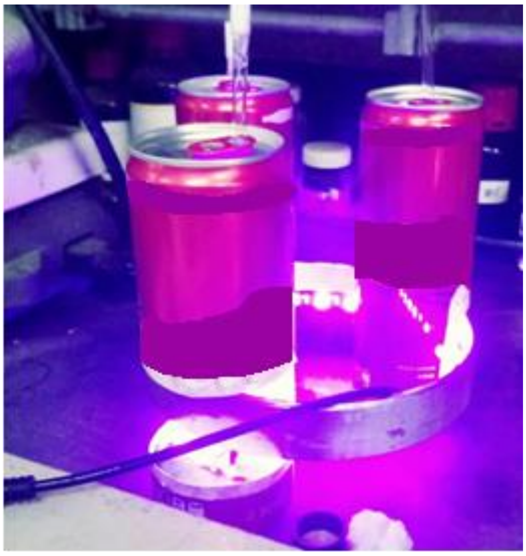

Figure S22. Image of photoirradiation experiment.

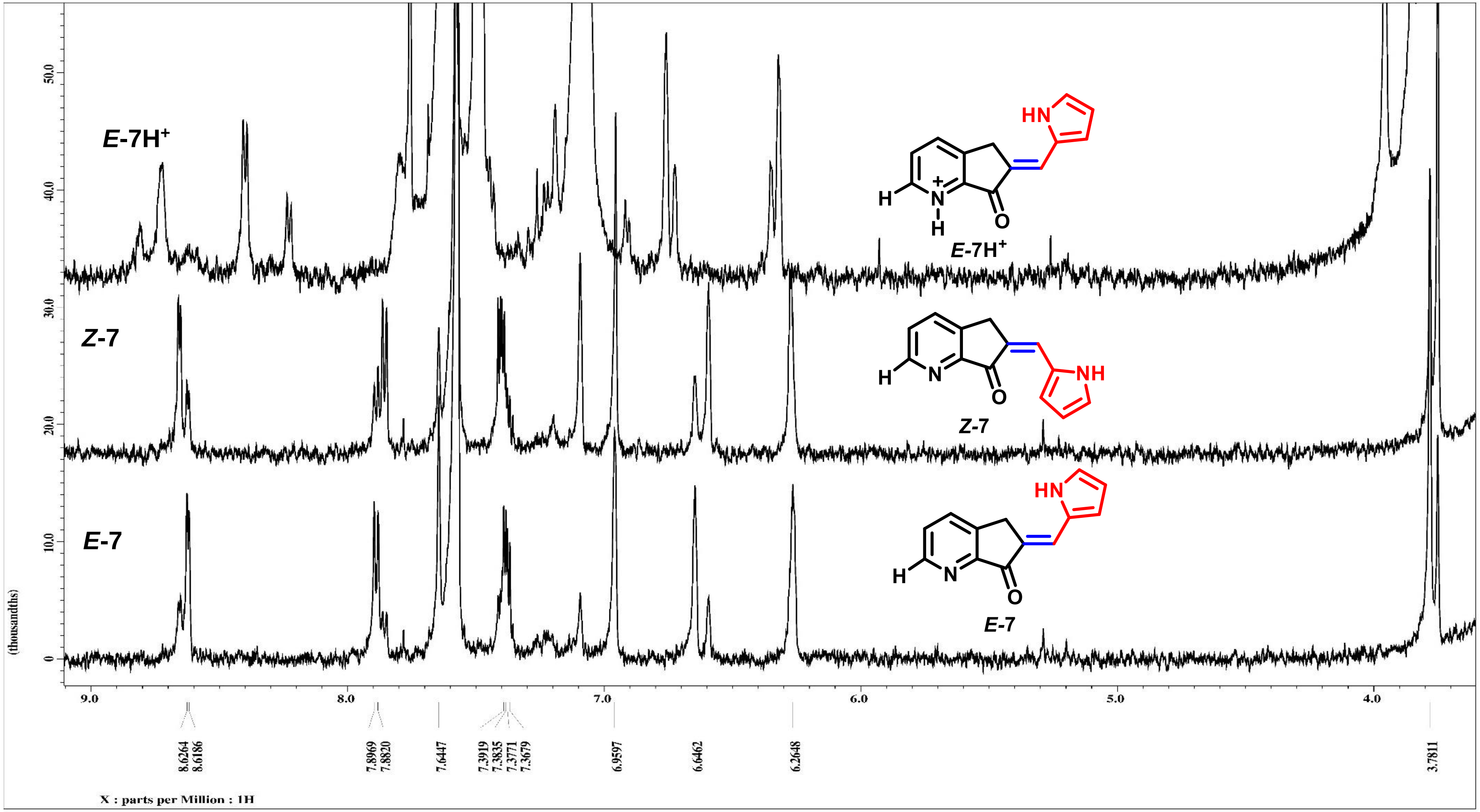

Figure S23. ^{1}H NMR spectrums of compounds ***E*-7**, ***Z*-7**, and ***E*-7H^{+}**.

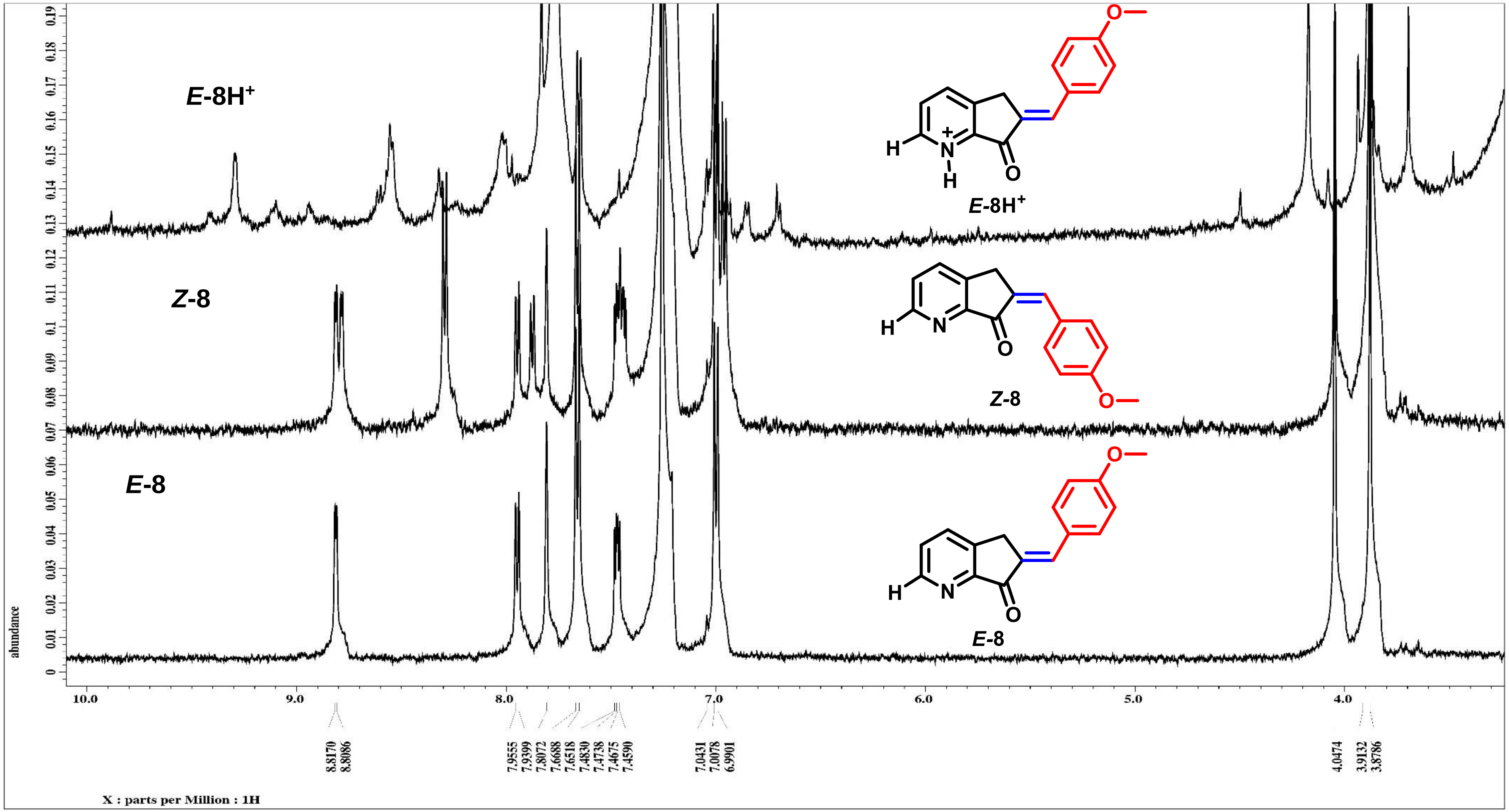

Figure S24. ^{1}H NMR spectrums of compounds ***E*-8**, ***Z*-8**, and ***E*-8H$^+$**.

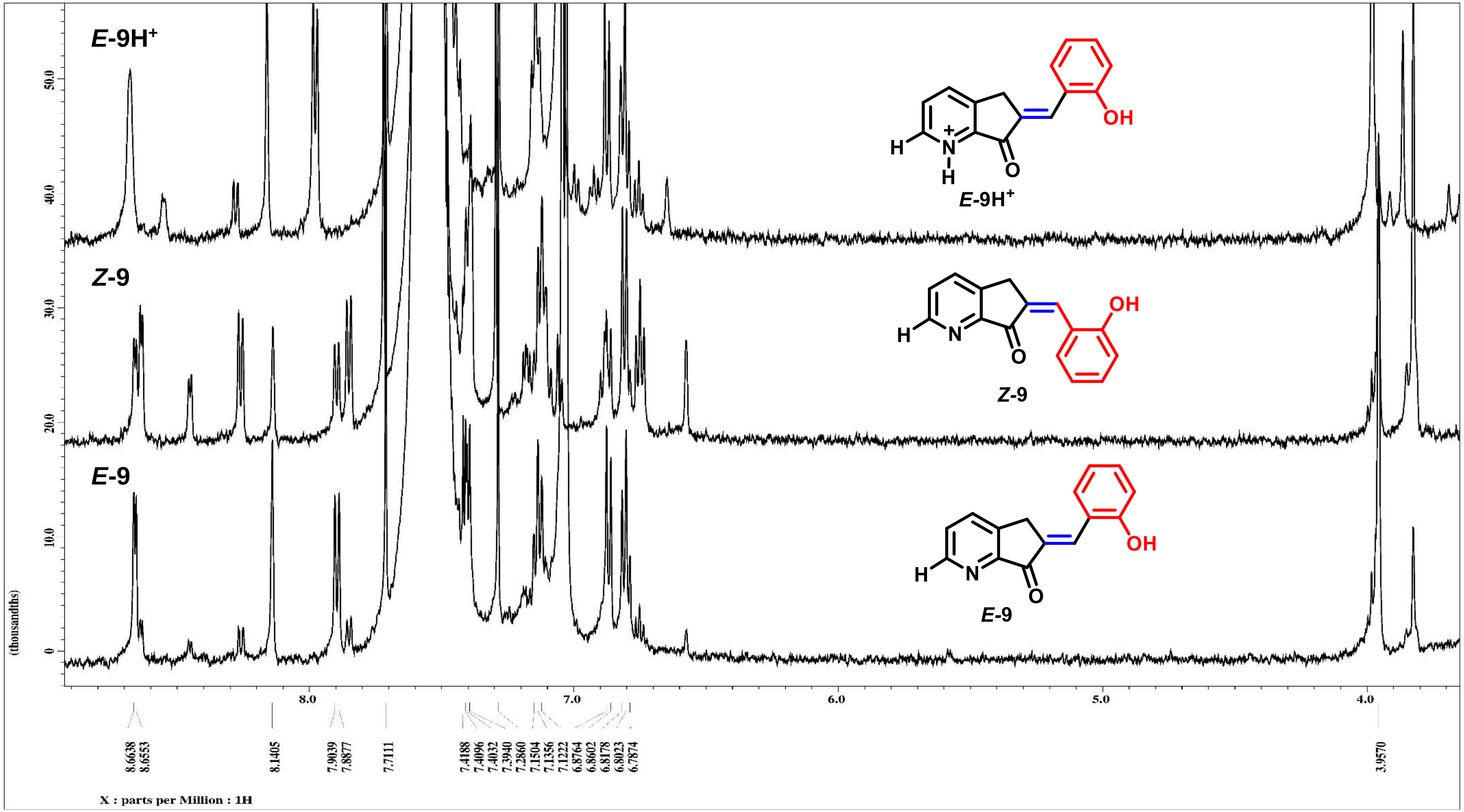

Figure S25. ^{1}H NMR spectrums of compounds ***E*-9**, ***Z*-9**, and ***E*-9H^{+}**.

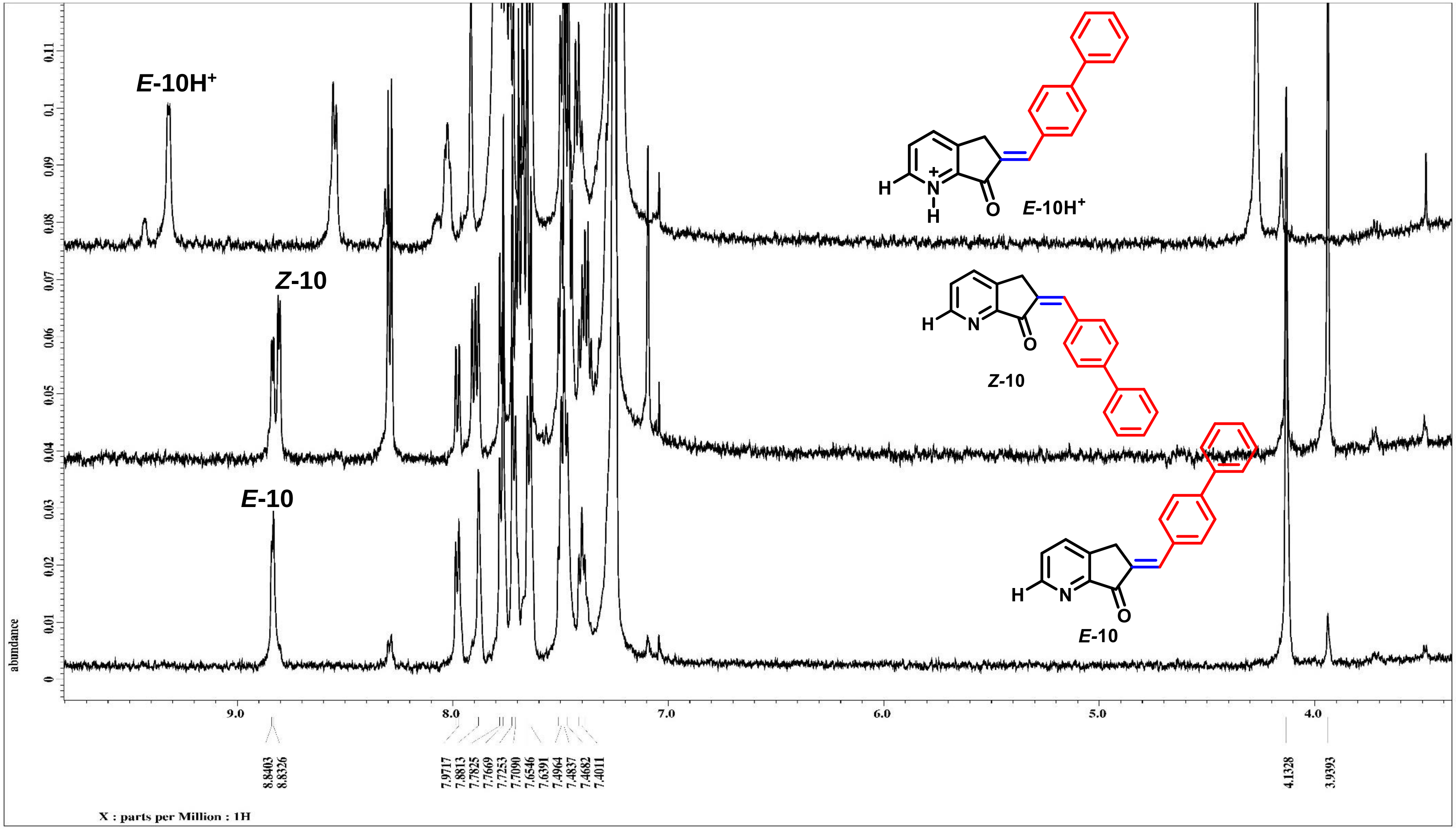

Figure S26. ^{1}H NMR spectrums of compounds ***E*-10**, ***Z*-10**, and ***E*-10H⁺**.

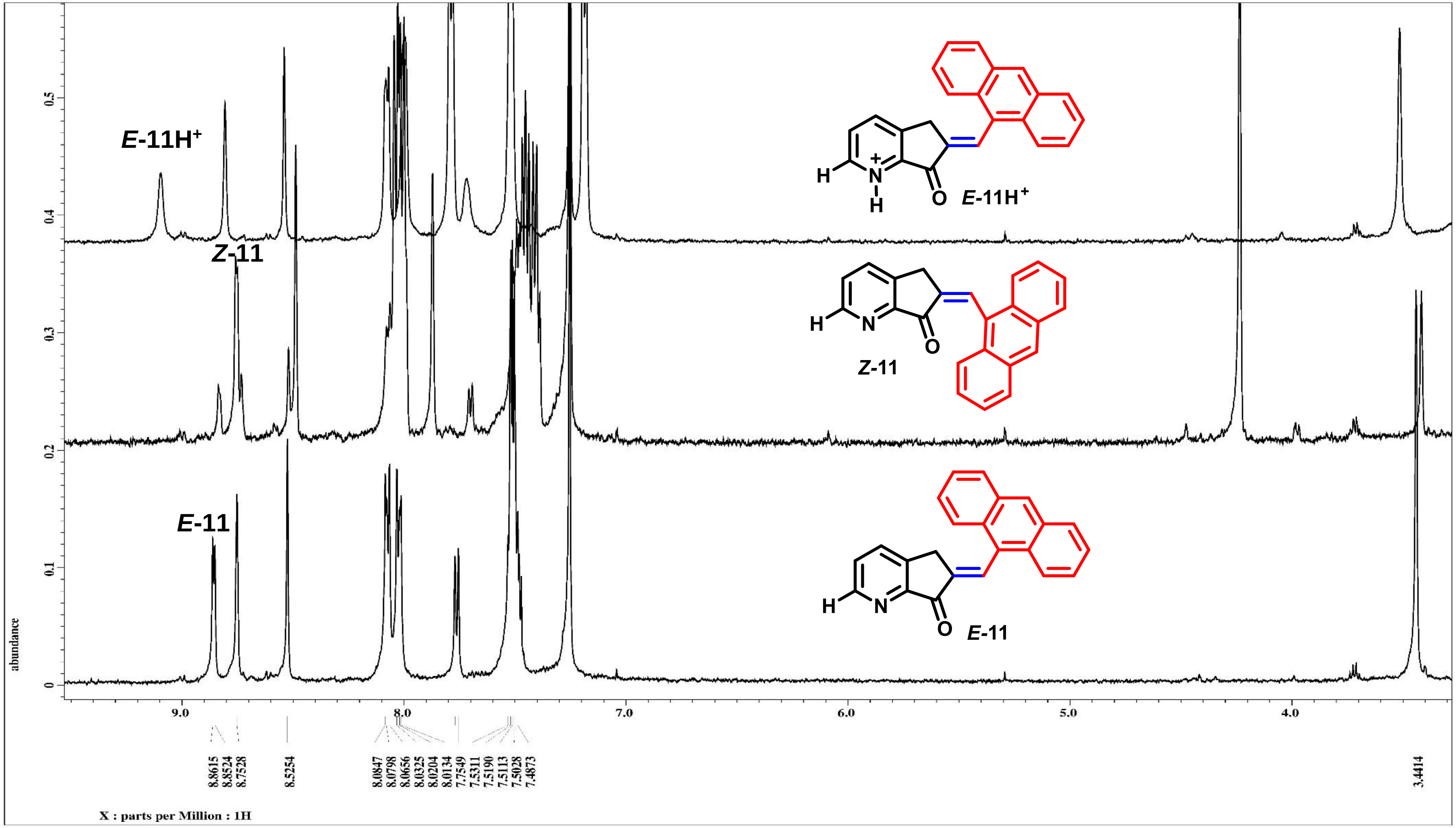

Figure S27. ^{1}H NMR spectrums of compounds ***E*-11**, ***Z*-11**, and ***E*-11H^{+}**.

MLG operation process

	Process	Molar Fraction ***E*-7**	Molar Fraction ***Z*-7**	δH1 ***E*-7**	δH1 ***Z*-7**
1	Initial Mixture	0.72	0.28	8.61	8.65
2	400nm x 10min	0.35	0.65	8.61	8.65
3	12 eq. pTSOH	0.65	0.35	8.7	8.77
4	15 eq. pTSOH	0.75	0.25	8.72	8.81
5	400nm x 10min	0.65	0.35	8.72	8.81
6	33,33 eq. TEA	0.71	0.29	8.62	8.65
7	400nm x 10min	0.39	0.61	8.62	8.65
8	445nm x 10min	0.87	0.13	8.62	8.65

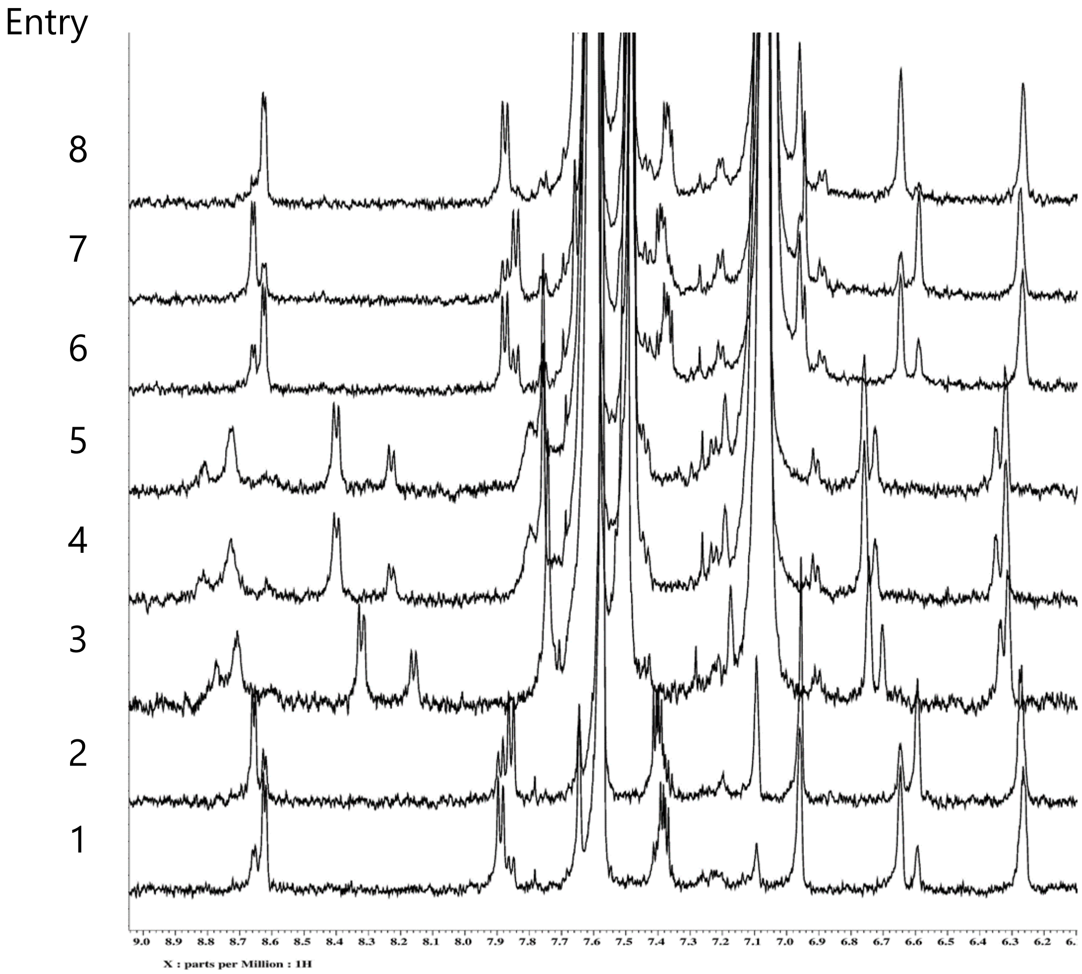

Figure S28. Scheme of MLG **7** operation process.

	Process	Molar Fraction ***E*-8**	Molar Fraction ***Z*-8**	δH1 ***E*-8**	δH1 ***Z*-8**
1	Initial Mixture	1	0	8.8	8.78
2	445nm x 10 min	1	0	8.8	8.78
3	400nm x 10 min	0.48	0.52	8.8	8.78
4	400nm x 10 min	0.48	0.52	8.8	8.78
5	10 min	0.48	0.52	8.8	8.78
6	445nm x 10 min	1	0	8.8	8.78
7	400nm x 10 min	0.48	0.52	8.8	8.78
8	2 Eq. pTsOH	1	0	9.17	9.36
9	400nm x 10 min	0.71	0.29	9.17	9.36
10	3.5 Eq. pTsOH	1	0	9.28	9.4
11	400nm x 10 min	0.81	0.19	9.28	9.4
12	6.16 Eq. TEA	0.81	0.19	8.8	8.78
13	400nm x 10 min	0.49	0.51	8.8	8.78
14	445nm x 10 min	1	0	8.8	8.78

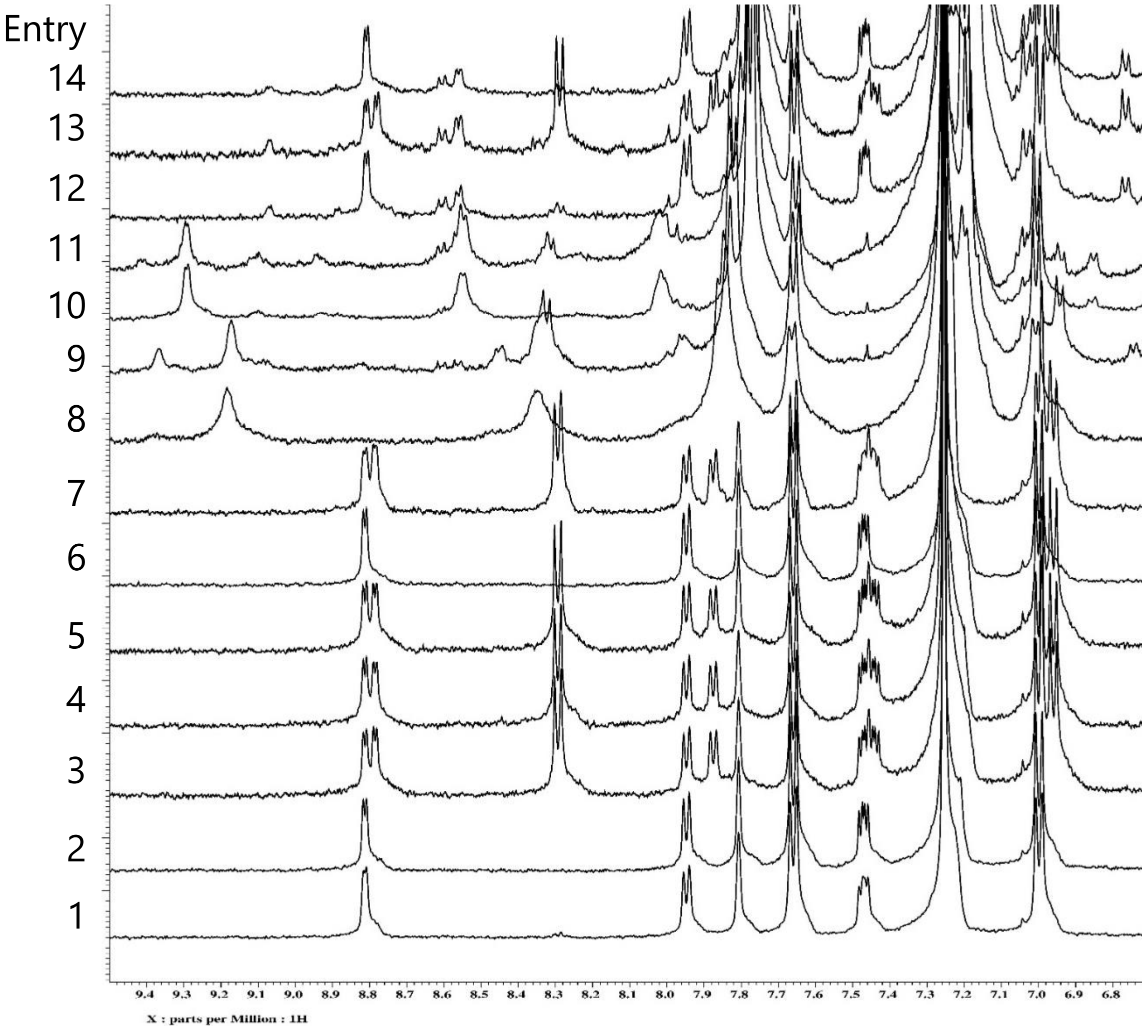

Figure S29. Scheme of MLG **8** operation process.

	Process	Molar Fraction ***E*-9**	Molar Fraction ***Z*-9**	δH1 ***E*-9**	δH1 ***Z*-9**
1	Initial Mixture	0.85	0.15	8.65	8.63
2	400nm x 10 min	0.38	0.62	8.65	8.63
3	400nm x 10 min	0.34	0.64	8.65	8.63
4	tiempo 20 min	0.4	0.6	8.65	8.63
5	2.3 Eq. pTsOH	0.58	0.42	8.66	8.66
6	3.1 Eq. pTsOH	0.63	0.27	8.67	8.67
7	400nm x 10 min	0.6	0.4	8.67	8.67
8	13.3 Eq. TEA	0.78	0.22	8.65	8.63
9	400nmx 10 min	0.37	0.63	8.65	8.63
10	58 Eq. TEA	0.43	0.57	8.65	8.63
11	103 Eq. TEA	0.57	0.43	8.65	8.63
12	400nm x 10 min	0.51	0.49	8.65	8.63
13	445nm x 10 min	0.93	0.07	8.65	8.63
14	400nm x 10 min	0.52	0.48	8.65	8.63

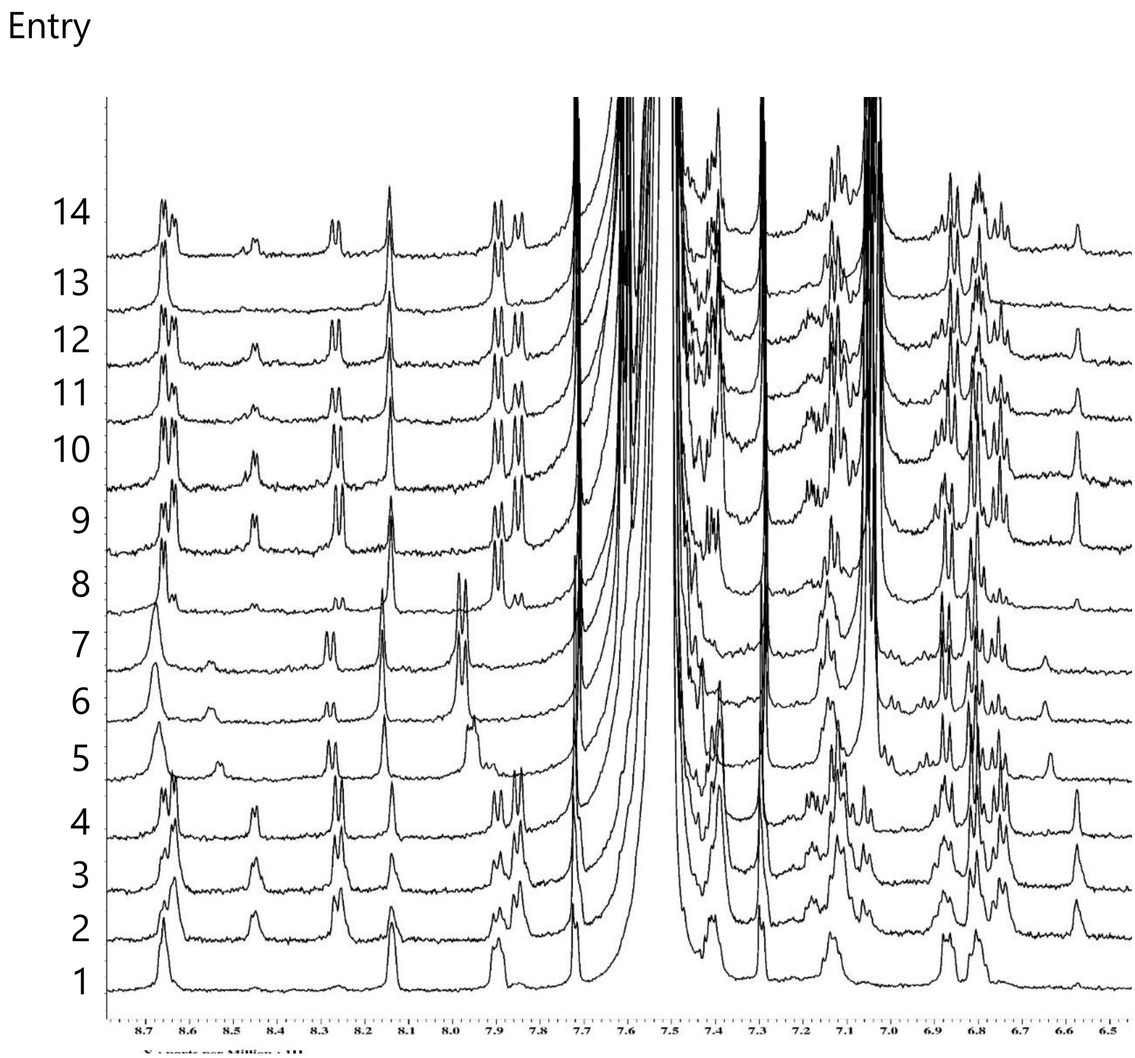

Figure S30. Scheme of MLG **9** operation process.

	Process	Molar Fraction ***E*-10**	Molar Fraction ***Z*-10**	δH1 ***E*-10**	δH1 ***Z*-10**
1	Initial Mixture	0.82	0.18	8.83	8.8
2	400nm x 10 min	0.81	0.19	8.83	8.8
3	400nm x 10 min	0.39	0.61	8.83	8.8
4	1.5 Eq. pTsOH	0.55	0.45	9.19	9.37
5	3 Eq. pTsOH	0.8	0.2	9.31	9.42
6	400nm x 10 min	0.69	0.31	9.31	9.42
7	445nm x 10 min	1	0	9.31	9.42
8	7 Eq. TEA	1	0	8.82	8.7
9	400nm x 10 min	0.43	0.57	8.82	8.8

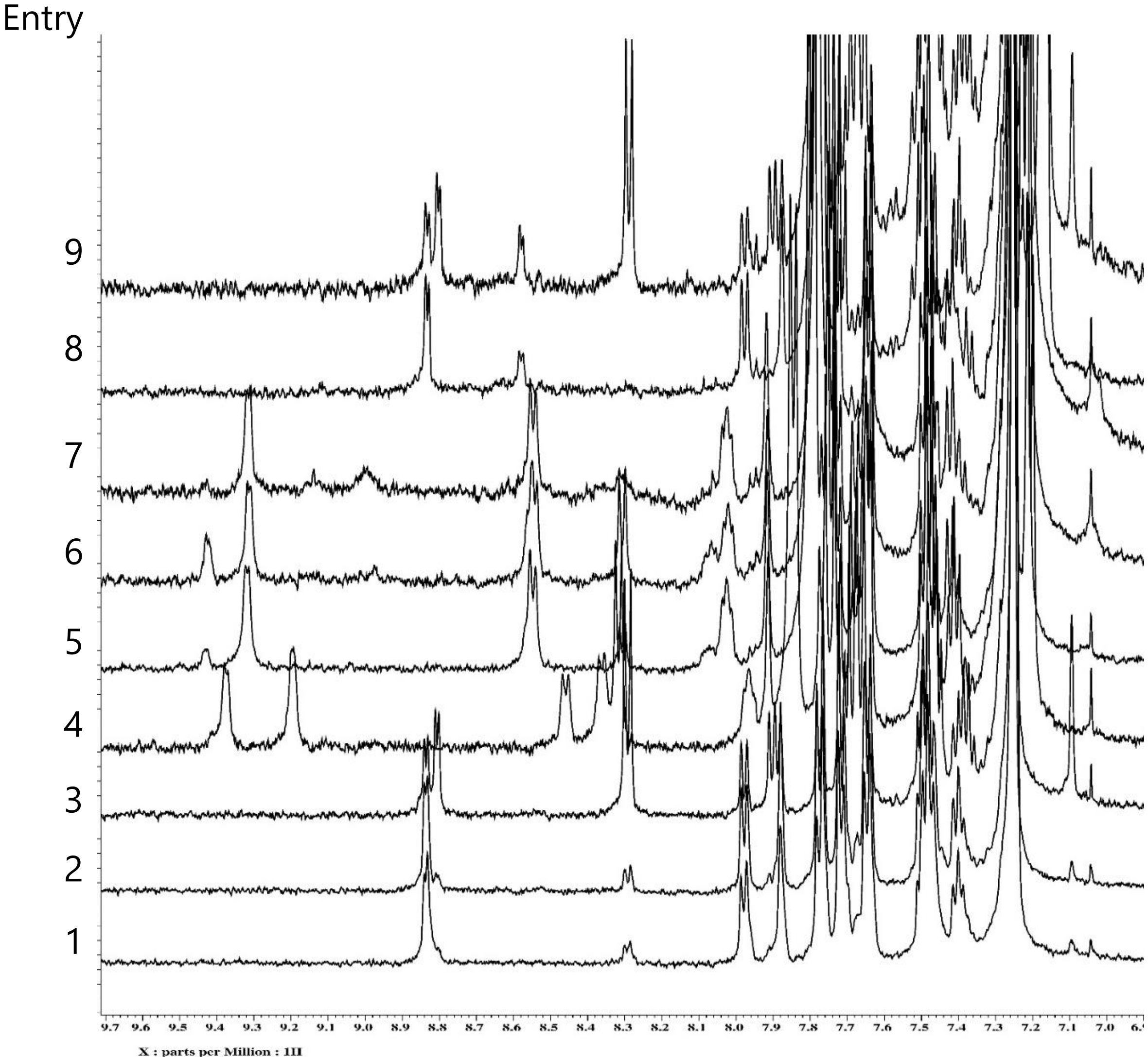

Figure S31. Scheme of MLG **10** operation process.

	Process	Molar Fraction ***E*-11**	Molar Fraction ***Z*-11**	Molar Fraction **iso-11**	δH1 ***E*-11**	δH1 ***Z*-11**
1	Initial Mixture	1	0	0	8.84	8.75
2	445nm X 2min	0.91	0.09	0	8.84	8.75
3	445nm X 5min	0.87	0.13	0	8.84	8.75
4	445nm X 10min	0.86	0.14	0	8.84	8.75
5	400nm x 20min	0.83	0.11	0.05	8.84	8.75
6	1 Eq. TEA	0.9	0.05	0.05	8.82	8.75
7	445nm X 10min	0.24	0.71	0.05	8.82	8.75
8	445nm x 5min	0.24	0.71	0.05	8.82	8.75
9	2 Eq. pTsOH	0.93	0.02	0.05	9.09	9.09
10	2.5 Eq. TEA	0.93	0.02	0.05	8.82	8.75
11	2.5 Eq. pTsOH	0.93	0.02	0.05	8.89	8.89
12	445nm X 10min	0.24	0.71	0.05	8.85	8.85
13	3 Eq. pTsOH	0.35	0.6	0.05	8.89	8.89
14	3.5 Eq. pTsOH	0.52	0.43	0.05	8.93	8.93
15	4 Eq. pTsOH	0.6	0.35	0.05	8.98	8.98
16	4.5 Eq. pTsOH	0.75	0.2	0.05	9.01	9.01
17	5 Eq. pTsOH	0.79	0.16	0.05	9.05	9.05
18	445nm x 10 min	0.56	0.39	0.05	9.02	9.02
19	5.5 Eq. pTSOH	0.85	0.1	0.05	9.1	9.1
20	445nm x 10 min	0.62	0.33	0.05	9.07	9.07
21	12 Eq. TEA	0.77	0.18	0.05	8.82	8.75
22	445nm x 10 min	0.2	0.75	0.05	8.81	8.74

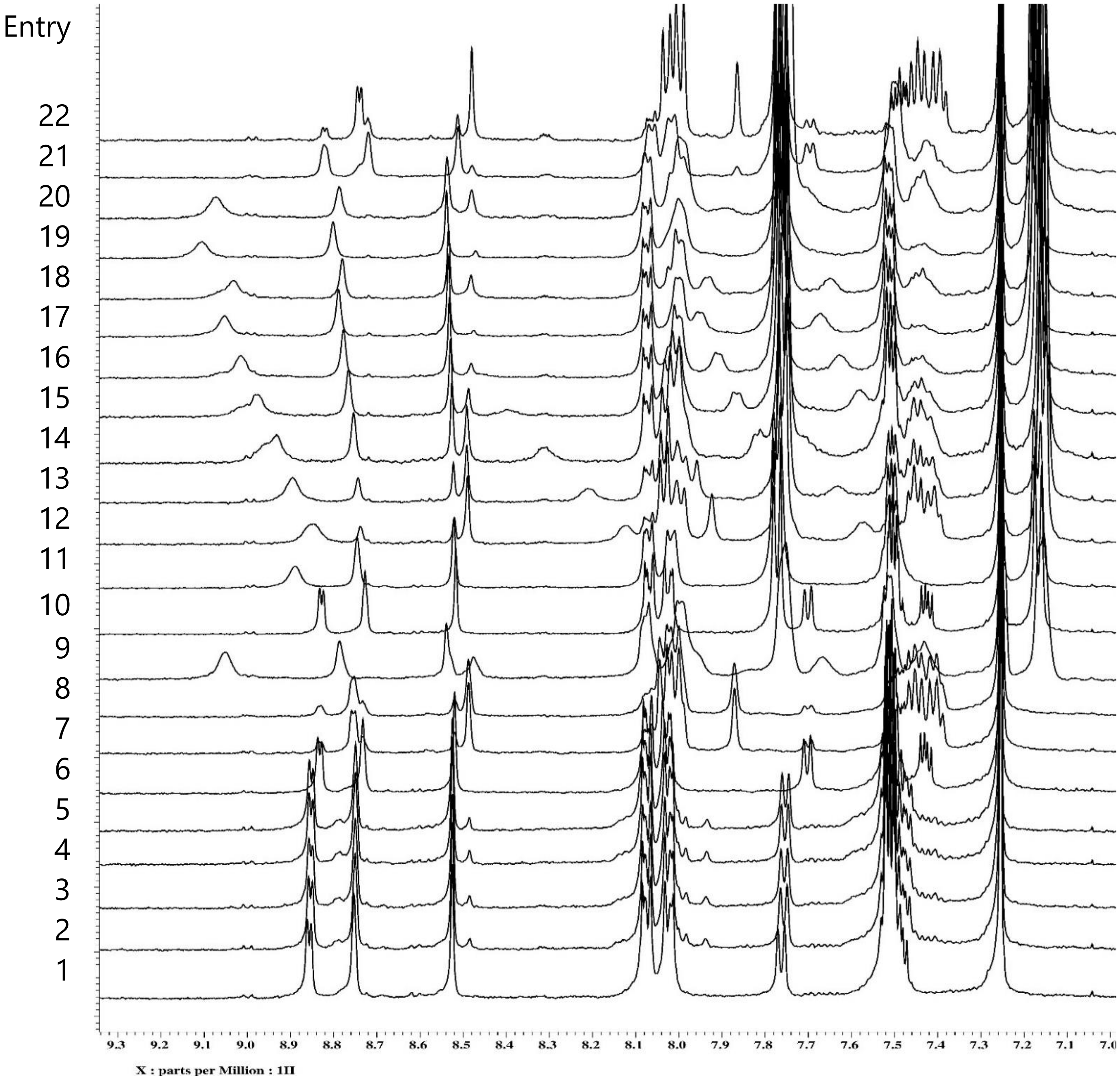

Figure S32. Scheme of MLG **11** operation process.

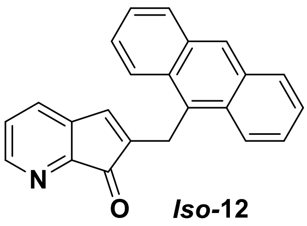

***Iso*-12**

Chart S1. Proposed molecule of ***E*-12** decomposition by 400 nm light.

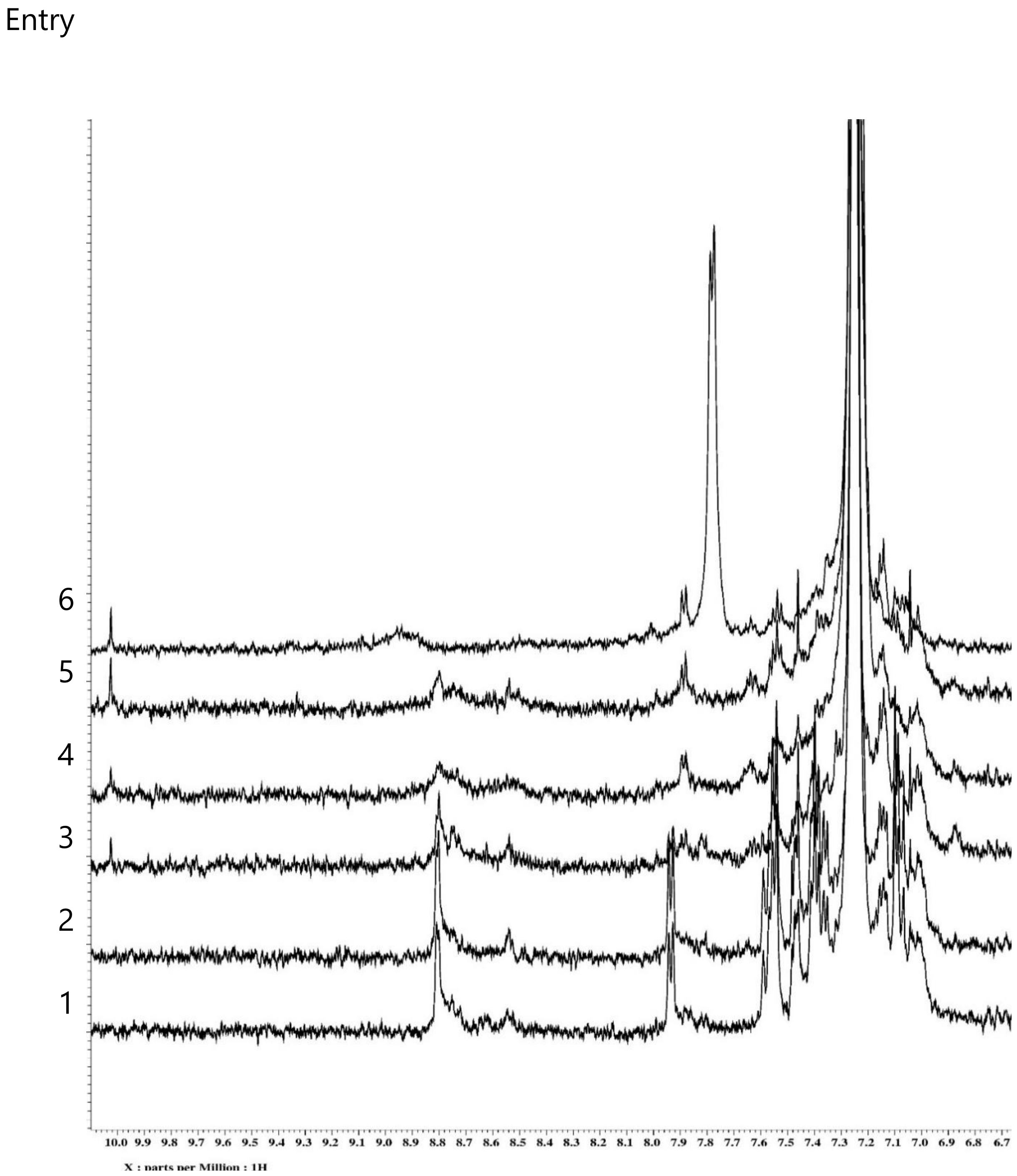

Figure S33. Scheme of MLG **12** operation process. Entry 2 and 5 correspond to the MLG **12** with visible light irradiation.

Description of Operation Process for MLG 8, 9, and 10.

The operation cycle of MLG **8** was tested starting with irradiation at 445 nm for 10 minutes where no response was observed. This was followed by irradiation at 400 nm for 10 minutes observing *E* to *Z* isomerization (entry 3, Scheme S2), followed by a second 10 min irradiation which had no effect on the isomer ratio (entry 4, Scheme S2). Next, the compound was irradiated at 445 nm for 10 minutes that led of *Z* to *E* isomerization where the *E* isomer was the major product (entry 6, Scheme S2) followed by further irradiation of the *Z* isomer at 400 nm 10 minutes (entry 7, Scheme S2). Then, two equivalents of pTsOH were added achieving *Z* to *E* reversal as confirmed by observation of the corresponding shifts in the NMR signals of both isomers (entry 8, Scheme S2). To verify that protonation slows down the isomerization of this MLG, the solution was irradiated at 400 nm light for 10 minutes observing minimal *E* to *Z* isomerization (entry 9, Scheme S2). The processes of entries 8 and 9 were repeated with the same isomerization pattern; the only difference was that when the 3.5 equivalents of pTsOH were added, the isomerization with 400 nm irradiation was minor in comparison with entry 9. To return to the initial isomer pattern, the solution was neutralized with 6.16 equivalents of triethylamine and irradiated with 400 nm light for 10 minutes to obtain the *Z* isomer as major product (entry 13, Scheme S2), with a pattern similar to entry 3. Finally, irradiation was performed at 445 nm for 10 minutes to complete the function of MLG **8**.

	Process	Molar Fraction ***E*-8**	Molar Fraction ***Z*-8**	δH1 ***E*-8**	δH1 ***Z*-8**
1	Initial Mixture	1	0	8.8	8.78
2	445nm x 10 min	1	0	8.8	8.78
3	400nm x 10 min	0.48	0.52	8.8	8.78
4	400nm x 10 min	0.48	0.52	8.8	8.78
5	10 min	0.48	0.52	8.8	8.78
6	445nm x 10 min	1	0	8.8	8.78
7	400nm x 10 min	0.48	0.52	8.8	8.78
8	2 Eq. pTsOH	1	0	9.17	9.36
9	400nm x 10 min	0.71	0.29	9.17	9.36
10	3.5 Eq. pTsOH	1	0	9.28	9.4
11	400nm x 10 min	0.81	0.19	9.28	9.4
12	6.16 Eq. TEA	0.81	0.19	8.8	8.78
13	400nm x 10 min	0.49	0.51	8.8	8.78
14	445nm x 10 min	1	0	8.8	8.78

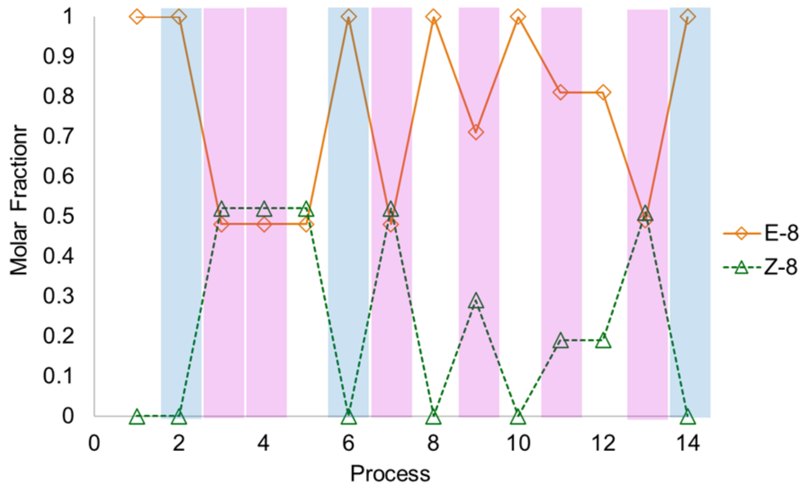

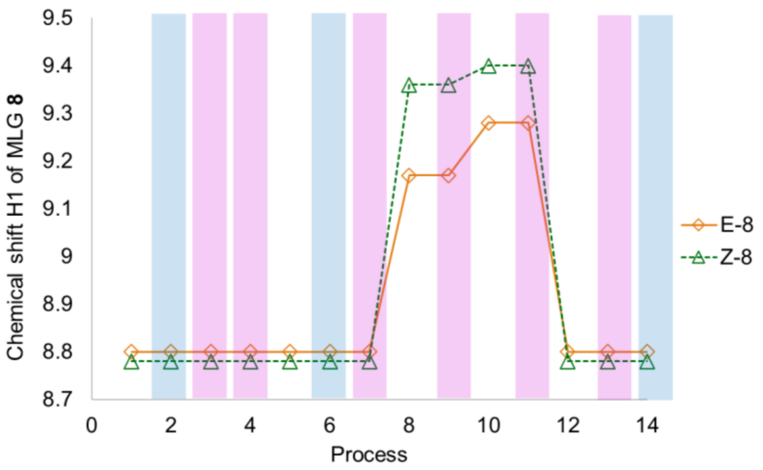

Scheme S1. Operation cycle of MLG **8**. Each process was recorded by NMR at 500 MHz in $CDCl_3$ at room temperature. The concentration of MLG used in these experiments was 2 mM.

The study of MLG **9** started with irradiation of a solution with 400 nm light for 10 minutes to produce E to Z isomerization (entry 2, Scheme S3). To verify that the isomerization was caused by irradiation, the mixture with the Z isomer as main compound was irradiated with 400 nm light for 10 minutes, observing a slight increase of the compound (entry 3, Scheme S3). To the mixture with the Z isomer as the major isomer were added 2.3 equivalents of p TsOH to achieve Z to E isomerization. The first isomerization gave low proportion ofthe expected isomer, however, with additional 3.1 equivalents of pTsOH, an increase in the *E* isomer which was confirmed by a shift of the signals detected by NMR for both isomers (entries 5 and 6 Scheme S3). To verify the protonation effect, the sample was irradiated with 400 nm light for 10 minutes, and the isomerization decreased (entry 7, Scheme S3). With reference to the isomerization capability of MLG **9**, the solution was neutralized with 13.3 equivalents of triethylamine, irradiated with 400 nm light for 10 minutes observing *E* to *Z* isomerization (entry 9, Scheme S3). It should be mentioned that up to 103 equivalents of triethylamine were added, and no effect was detected with the excess of the base. The isomerization was slowed down but without a significant response (entry 11, Scheme S3). After the addition of excess triethylamine, the solution was irradiated at 400 nm for 10 minutes, evidencing a *E* to *Z* isomerization pattern similar to entry 2. The solution was irradiated at 445 nm for 10 minutes, and *Z* to *E* isomerization was observed (entry 13, Scheme S3). Finally, *E* to *Z* transformation was attained with 400 nm light for 10 minutes (entry 14, Scheme S3) to close the operation cycle of MLG **9**.

	Process	Molar Fraction ***E*-9**	Molar Fraction ***Z*-9**	δH1 ***E*-9**	δH1 ***Z*-9**
1	Initial Mixture	0.85	0.15	8.65	8.63
2	400nm x 10 min	0.38	0.62	8.65	8.63
3	400nm x 10 min	0.34	0.64	8.65	8.63
4	tiempo 20 min	0.4	0.6	8.65	8.63
5	2.3 Eq. pTsOH	0.58	0.42	8.66	8.66
6	3.1 Eq. pTsOH	0.63	0.27	8.67	8.67
7	400nm x 10 min	0.6	0.4	8.67	8.67
8	13.3 Eq. TEA	0.78	0.22	8.65	8.63
9	400nmx 10 min	0.37	0.63	8.65	8.63
10	58 Eq. TEA	0.43	0.57	8.65	8.63
11	103 Eq. TEA	0.57	0.43	8.65	8.63
12	400nm x 10 min	0.51	0.49	8.65	8.63
13	445nm x 10 min	0.93	0.07	8.65	8.63
14	400nm x 10 min	0.52	0.48	8.65	8.63

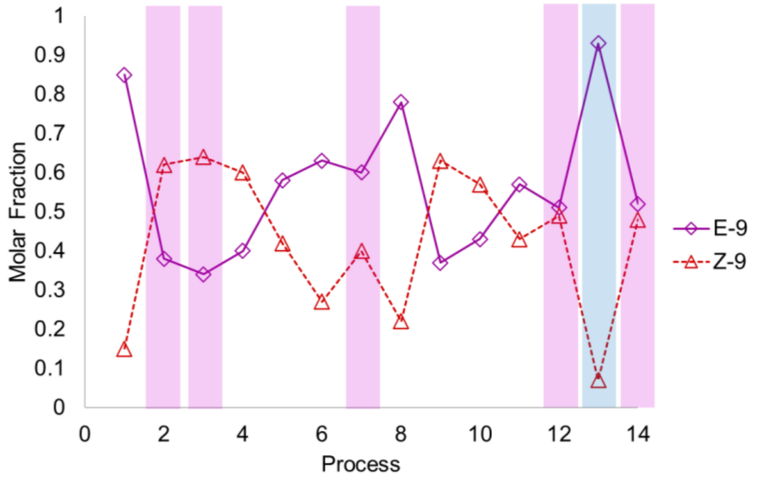

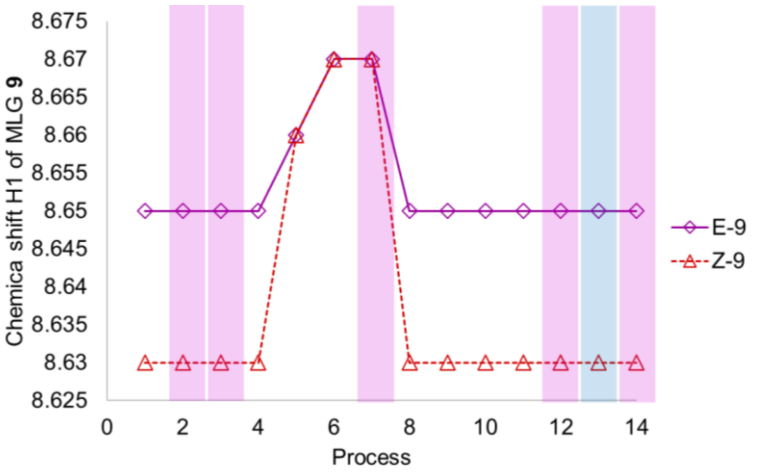

Scheme S2. Operation cycle of MLG **9**. Each process was recorded by NMR at 500 MHz in $CDCl_3$ at room temperature. The concentration of MLG used in these experiments was 2 mM.

The investigation of MLG **10** started with irradiation with 400 nm light for 20 minutes (to analyze and compare the effect of time with the same MLG and others) to achieve *E* to *Z* isomerization (entry 3, Scheme S4), giving the *Z* compound as the main product. This mixture was treated with 3 equivalents of pTsOH until the ^{1}H NMR signals shifted toward higher frequency, confirming *Z* to *E* isomerization (entry 5, Scheme S4). When the solution had the *E* isomer as the major product, the effect of protonation by irradiation with 400 nm light for 10 minutes resulted in no further isomerization of the *Z* isomer (entry 6, Scheme S4). Next, in our study, we tested the compound using 445 nm light and determined the complete transformation to the *E* isomer (entry 7, Scheme S4). Subsequently, the solution was neutralized with 7 equivalents of triethylamine, and it was irradiated again with 400 nm light for 10 minutes, obtaining a high ratio of the *Z* isomer (entry 9, Scheme S4), thus completing the operation cycle.

	Process	Molar Fraction ***E*-10**	Molar Fraction ***Z*-10**	δH1 ***trans*-10**	δH1 ***cis*-10**
1	Initial Mixture	0.82	0.18	8.83	8.8
2	400nm x 10 min	0.81	0.19	8.83	8.8
3	400nm x 10 min	0.39	0.61	8.83	8.8
4	1.5 Eq. pTsOH	0.55	0.45	9.19	9.37
5	3 Eq. pTsOH	0.8	0.2	9.31	9.42
6	400nm x 10 min	0.69	0.31	9.31	9.42
7	445nm x 10 min	1	0	9.31	9.42
8	7 Eq. TEA	1	0	8.82	8.7
9	400nm x 10 min	0.43	0.57	8.82	8.8

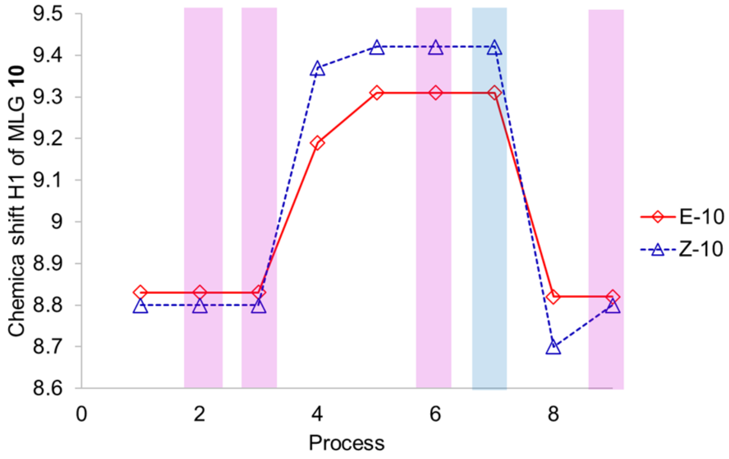

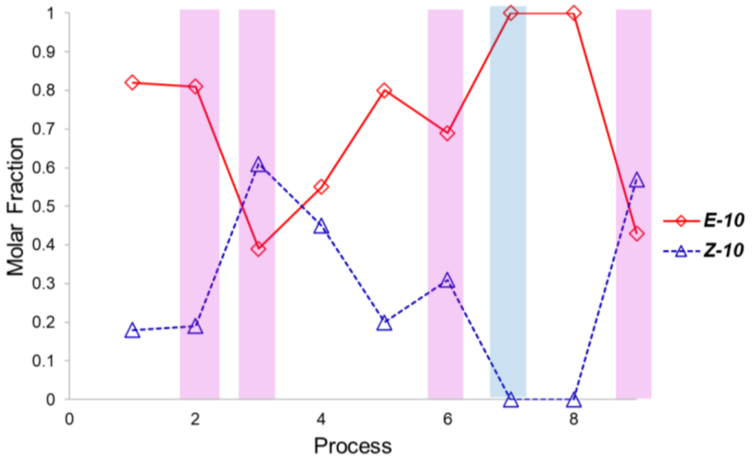

Scheme S3. Operation cycle of MLG **10**. Each process was recorded by NMR at 500 MHz in $CDCl_3$ at room temperature. The concentration of MLG used in these experiments was 2 mM.

Table S2. Summary of results for each process performed to verify MLGs **7** and **11.**

	Process	***E*-7**	Z-**7**	δH ***E*-7**	δH ***Z*-7**	***E*-11**	***Z*-11**	δH ***E*-11**	δH ***Z*-11**
1	Initial Mixture	0.8	0.2	8.61	8.65	0.8	0.2	8.68	8.6
2	445nm X 10min	0.88	0.12	8.61	8.65	0.2	0.8	8.68	8.6
3	445nm X 10min	0.88	0.12	8.61	8.65	0.2	0.8	8.68	8.6
4	400nm x 7min	0.34	0.66	8.61	8.65	0.3	0.7	8.68	8.6
5	400nm x 7min	0.3	0.7	8.61	8.65	0.3	0.7	8.68	8.6
6	1 Eq. pTsOH	0.33	0.67	8.63	8.69	0.35	0.65	8.69	8.61
7	2 Eq. pTsOH	0.43	0.57	8.65	8.73	0.38	0.62	8.69	8.62
8	4 Eq. pTsOH	0.43	0.57	8.68	8.81	0.46	0.54	8.7	8.63
9	20 Eq. TEA	0.46	0.54	8.61	8.65	0.43	0.57	8.68	8.6
10	445nm x 10min	0.99	0.01	8.61	8.65	0.19	0.81	8.68	8.6
11	400nm x 7min	0.27	0.73	8.61	8.65	0.25	0.75	8.68	8.6
12	23 Eq. pTsOH	0.47	0.53	8.68	8.93	0.4	0.6	8.74	8.63

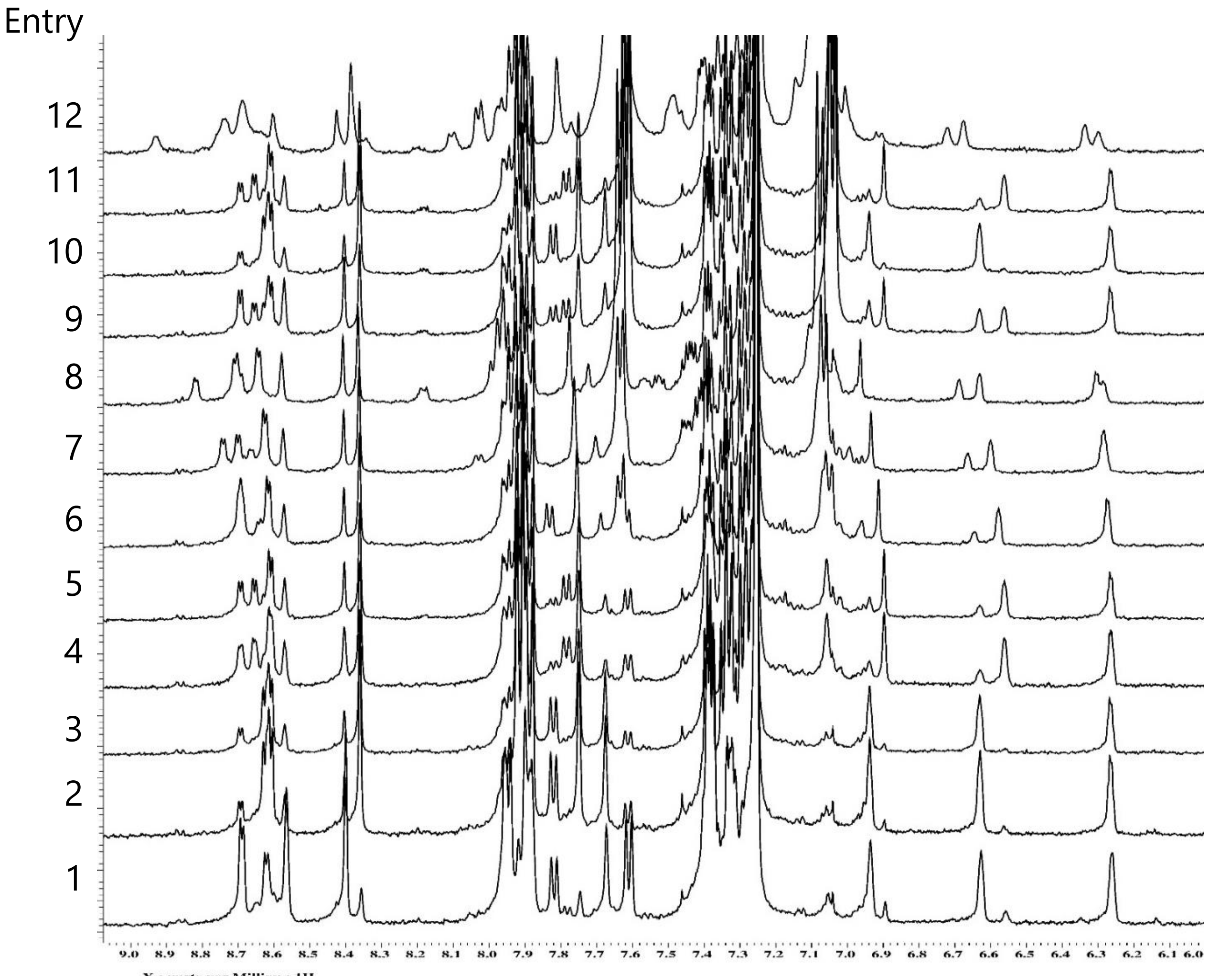

Figure S34. Scheme of MLG **7** and **11** operating in the same NMR tube.

Table S3. Summary of method of operation for the mixture of MLGs **7** and **11**, outputs and corresponding labels

	Process	Output				Label
		I	II	III	IV	
1	Initial mixture	0	0	0	0	a
2	445nm X 10min	0	0	0	1	b
3	445nm X 10min	0	0	0	1	b
4	400nm x 7min	0	1	0	1	f
5	400nm x 7min	0	1	0	1	F
6	1 Eq. pTsOH	1	1	1	1	f-d
7	2 Eq. pTsOH	1	0	1	1	f-d
8	4 Eq. pTsOH	1	0	1	0	d
9	20 Eq. TEA	0	0	0	0	k
10	445nm x 10min	0	0	0	1	m
11	400nm x 7min	0	1	0	1	p
12	23 Eq. pTsOH	1	0	1	0	c

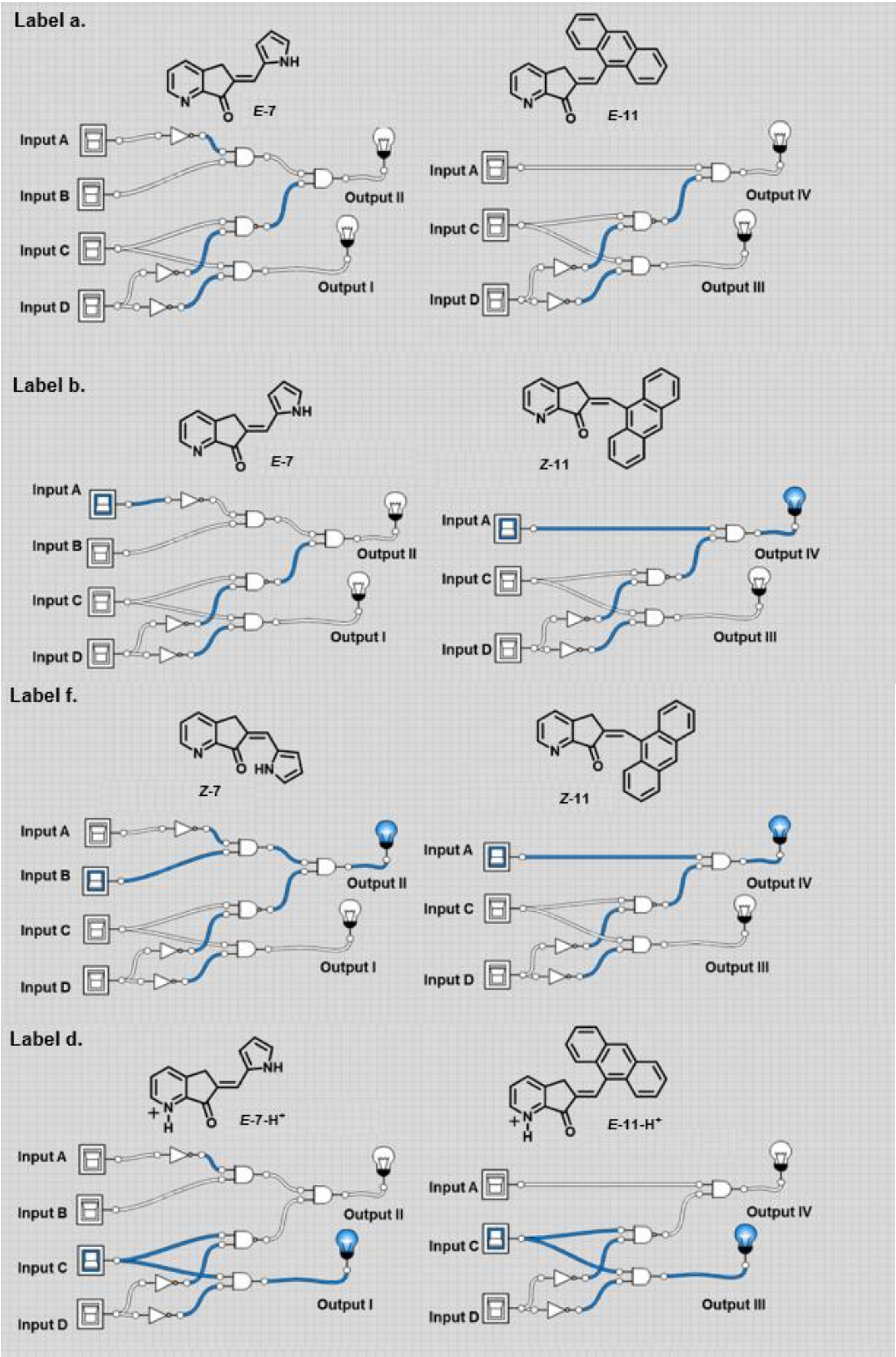
Label a.
E-7
E-11
Input A
Input B
Input C
Input D
Output II
Output I
Input A
Input C
Input D
Output IV
Output III
Label b.
E-7
Z-11
Input A
Input B
Input C
Input D
Output II
Output I
Input A
Input C
Input D
Output IV
Output III
Label f.
Z-7
Z-11
Input A
Input B
Input C
Input D
Output II
Output I
Input A
Input C
Input D
Output IV
Output III
Label d.
E-7-H+
E-11-H+
Input A
Input B
Input C
Input D
Output II
Output I
Input A
Input C
Input D
Output IV
Output III

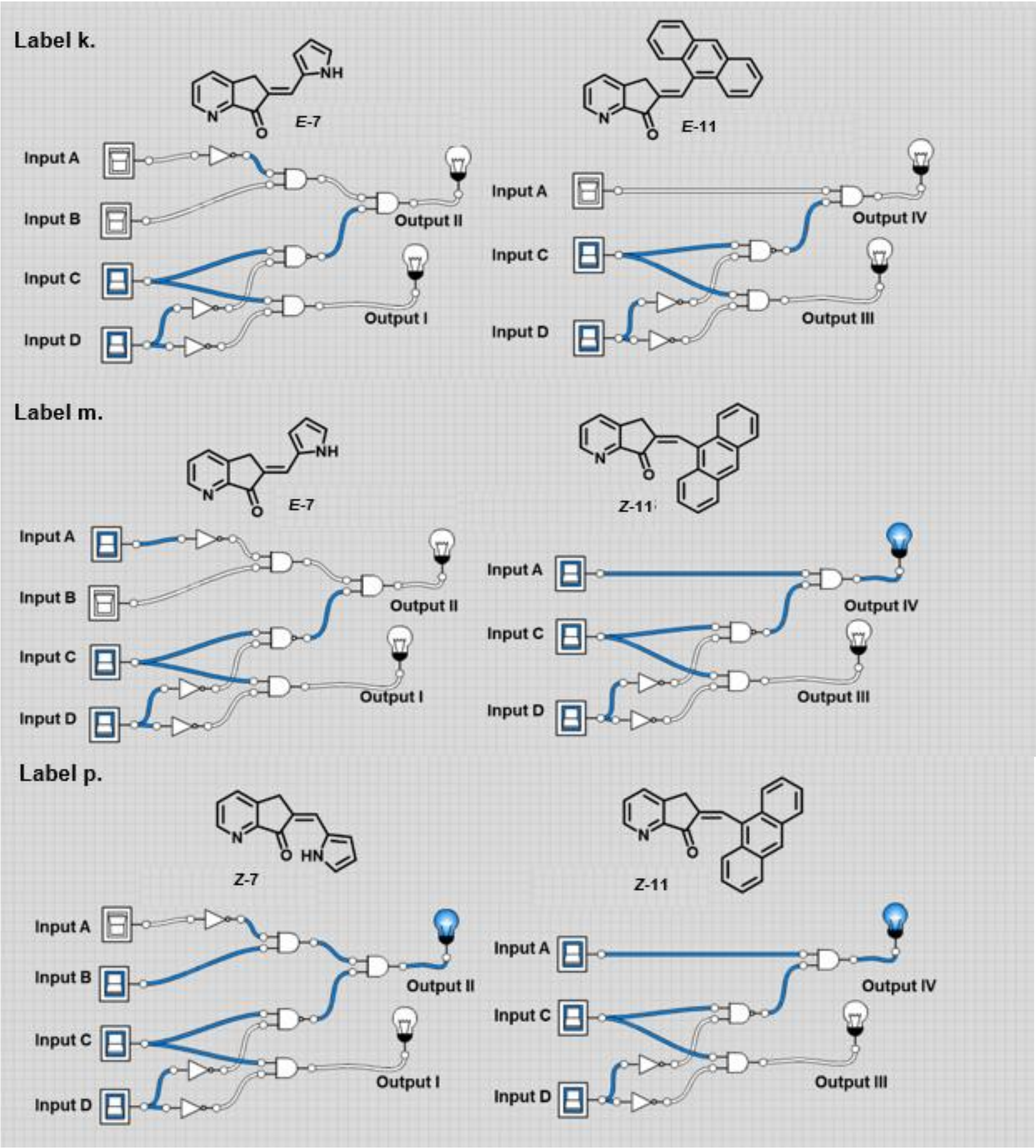

Scheme S4. Diagrams show how the logical operation is solved with MLGs **7** and **11** of each process carried out in the experiments with the theoretical labels. The concentrations of the two MLGs were 1.5 mM in $CDCl_3$:DMSO-d_6 (3:0.2 volume ratio), and NMR recorded each process at 500 MHz at room temperature.

Table S4. Summary of results of each process performed to verify MLGs **7**, **10** and **11.**

Process		***E*- 7**	***Z*- 7**	δH ***E*-7**	δH ***Z*- 7**	***E*- 10**	***Z*- 10**	δH ***E*-10**	δH ***Z*-10**	***E*- 11**	***Z*-11**	δH ***E*-11**	δH ***Z*-11**
1	Initial Mixture	0.8	0.2	8.61	8.65	0.95	0.05	8.68	8.65	0.66	0.33	8.68	8.6
2	400nm x 5min	0.46	0.54	8.61	8.65	0.63	0.37	8.68	8.65	0.52	0.48	8.68	8.6
3	400nm x 10min	0.39	0.61	8.61	8.65	0.47	0.53	8.68	8.65	0.54	0.44	8.68	8.6
4	445nm x 5min	0.86	0.14	8.61	8.65	0.66	0.34	8.68	8.65	0.28	0.72	8.68	8.6
5	445nm x 10min	0.84	0.16	8.61	8.65	0.82	0.18	8.68	8.65	0.32	0.68	8.68	8.6
6	400nm x 15min	0.34	0.66	8.61	8.65	0.47	0.53	8.68	8.65	0.54	0.46	8.68	8.6
7	0.7 Eq. pTsOH	0.34	0.66	8.62	8.65	0.47	0.53	8.68	8.65	0.54	0.46	8.68	8.6
8	1.6 Eq. pTsOH	0.38	0.62	8.64	8.66	0.49	0.51	8.69	8.66	0.6	0.4	8.69	8.6
9	3.3 Eq. pTsOH	0.46	0.54	8.66	8.67	0.52	0.48	8.69	8.67	0.6	0.4	8.69	8.61
10	4 Eq. pTsOH	0.58	0.42	8.67	8.67	0.64	0.36	8.69	8.67	0.62	0.37	8.69	8.61
11	7 Eq. pTsOH	0.71	0.29	8.7	8.7	0.73	0.27	8.7	8.7	0.64	0.36	8.7	8.63
12	400nm x 10min	0.71	0.29	8.7	8.7	0.6	0.4	8.7	8.7	0.64	0.36	8.7	8.62
13	10.5 Eq. pTsOH	0.73	0.27	8.71	8.71	0.68	0.32	8.71	8.71	0.64	0.36	8.71	8.64
14	25 Eq. TEA	0.73	0.27	8.61	8.65	0.68	0.32	8.68	8.65	0.64	0.36	8.68	8.6
15	400nm x 10min	0.4	0.6	8.61	8.65	0.46	0.54	8.68	8.65	0.6	0.4	8.68	8.6

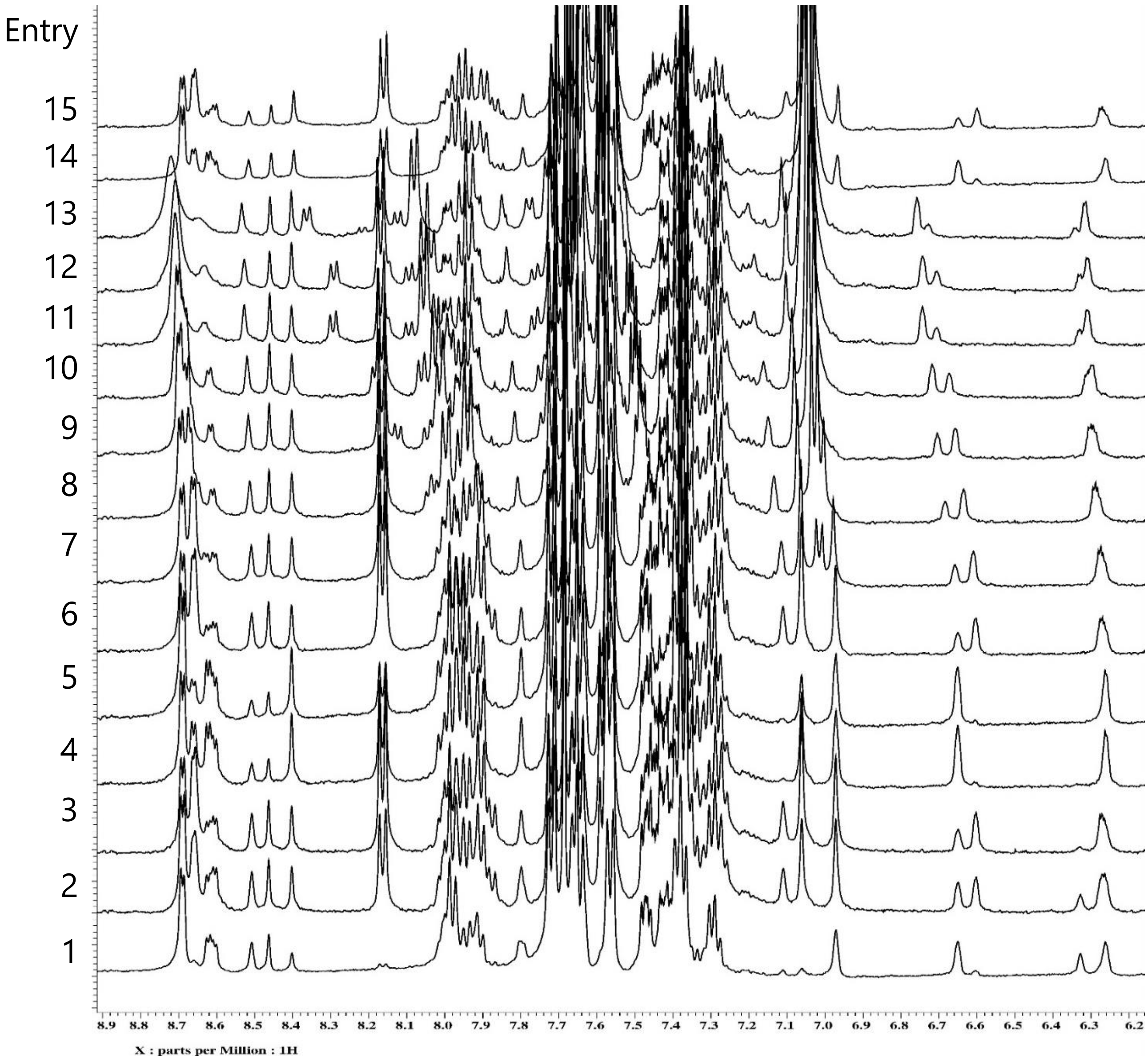

Figure S35. Scheme of operating process for MLG **7, 10** and **11** placed in the same NMR tube.

Table S5. Summary of method of operation for the mixture of MLGs **7**, **10** and **11**, outputs and corresponding labels

	Process	Output						Label
		I	II	III	IV	V	VI	
1	Initial solution	0	0	0	0	0	0	a
2	400nm x 5min	0	1	0	0	0	0	a-c
3	400nm x 10min	0	1	0	1	0	0	c
4	445nm x 5min	0	0	0	0	0	1	f1
5	445nm x 10min	0	0	0	0	0	1	f1
6	400nm x 15min	0	1	0	1	0	0	f2
7	0.7 Eq. pTsOH	0	1	0	1	0	0	f2-d
8	1.6 Eq. pTsOH	0	1	0	1	0	0	f2-d
9	3.3 Eq. pTsOH	1	1	1	0	0	0	f2-d
10	4 Eq. pTsOH	1	0	1	0	0	0	f2-d
11	7 Eq. pTsOH	1	0	1	0	1	0	d
12	400nm x 10min	1	0	1	0	1	0	i
13	10.5 Eq. pTsOH	1	0	1	0	1	0	d
14	25 Eq. TEA	0	0	0	0	0	0	k
15	400nm x 10min	0	1	0	1	0	0	n

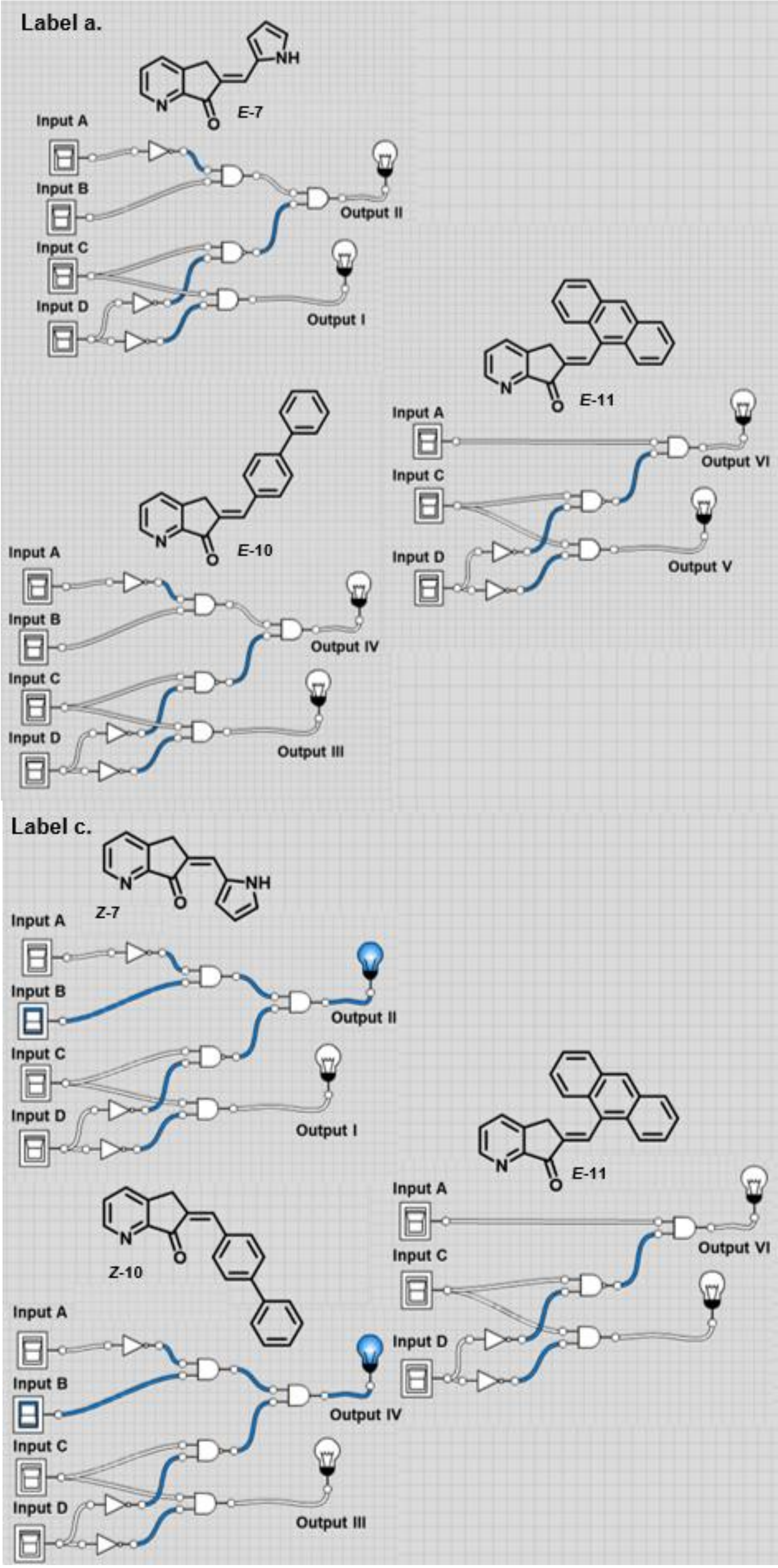
Label a.
E-7
Input A
Input B
Input C
Input D
Output II
Output I
E-11
Input A
Input C
Input D
Output VI
Output V
E-10
Input A
Input B
Input C
Input D
Output IV
Output III
Label c.
Z-7
Input A
Input B
Input C
Input D
Output II
Output I
E-11
Input A
Input C
Input D
Output VI
Z-10
Input A
Input B
Input C
Input D
Output IV
Output III

Label f1.

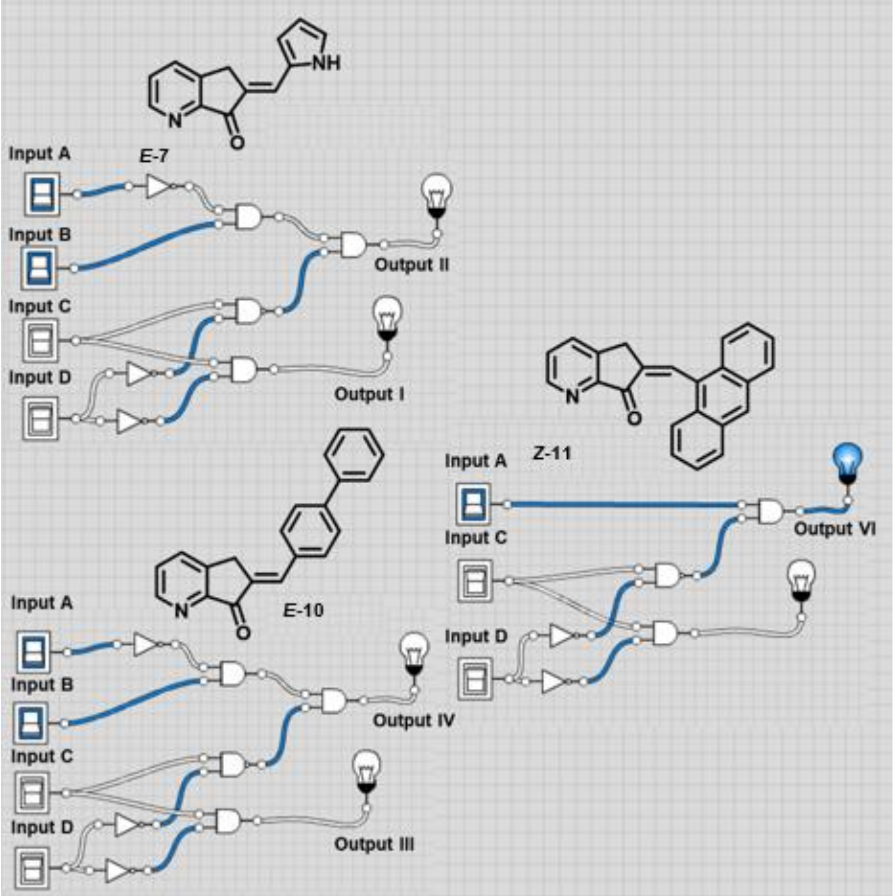

Label f2.

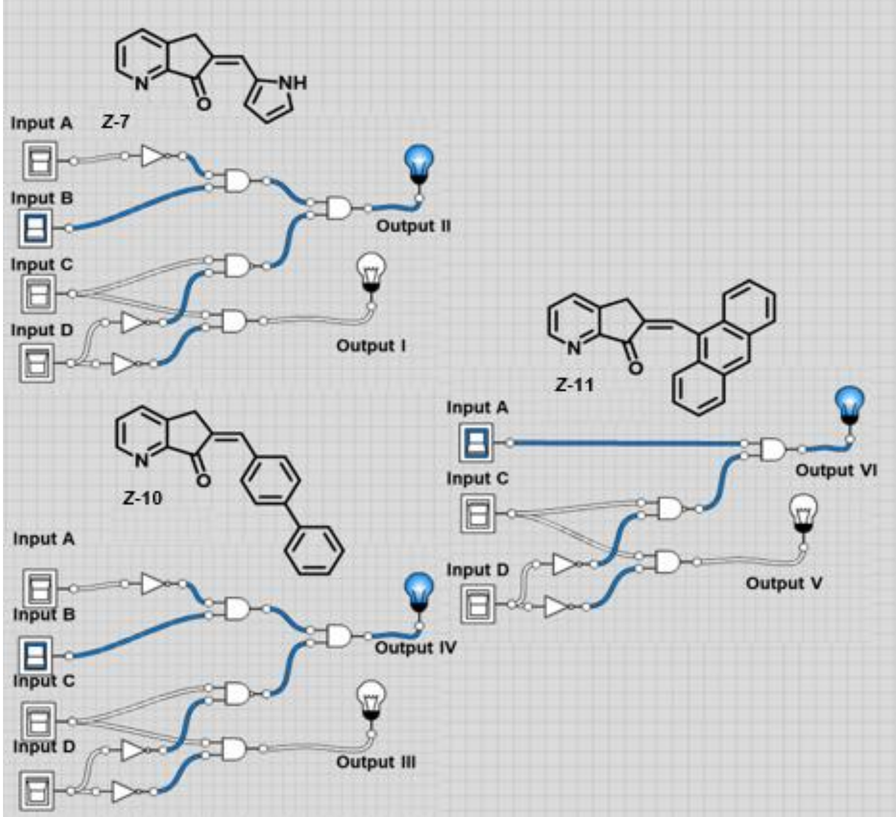

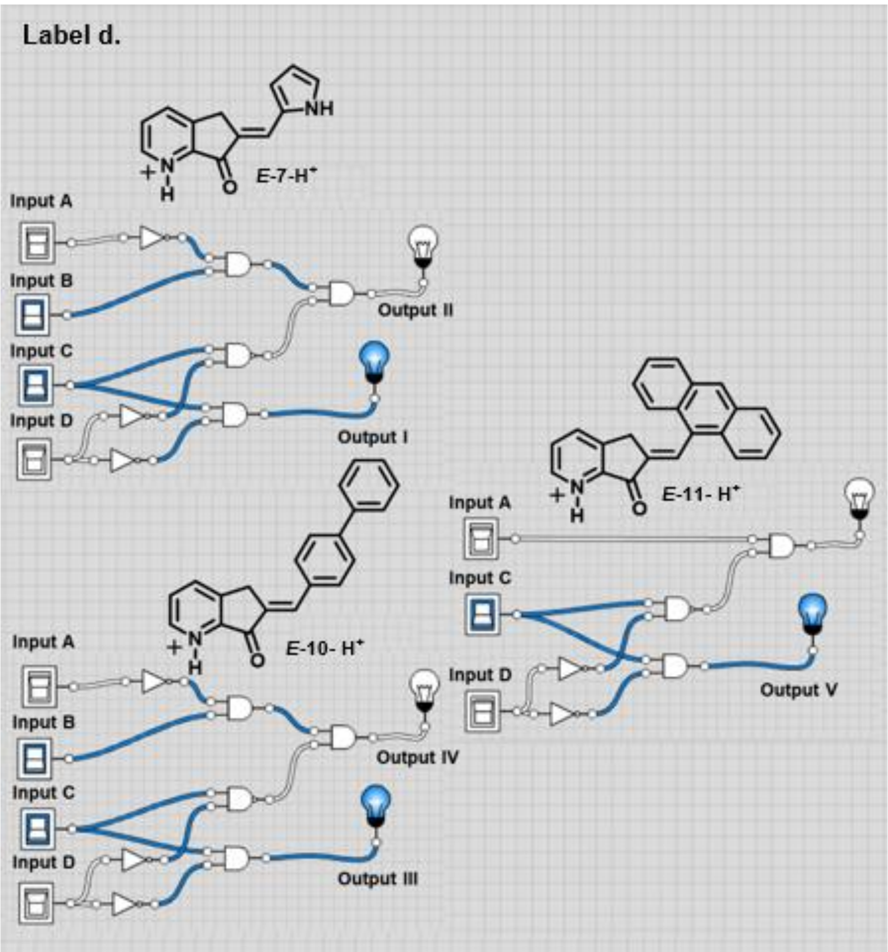
Label d.
E-7-H+
Input A
Input B
Input C
Input D
Output II
Output I
E-10- H+
Input A
Input B
Input C
Input D
Output IV
Output III
E-11- H+
Input A
Input C
Input D
Output V

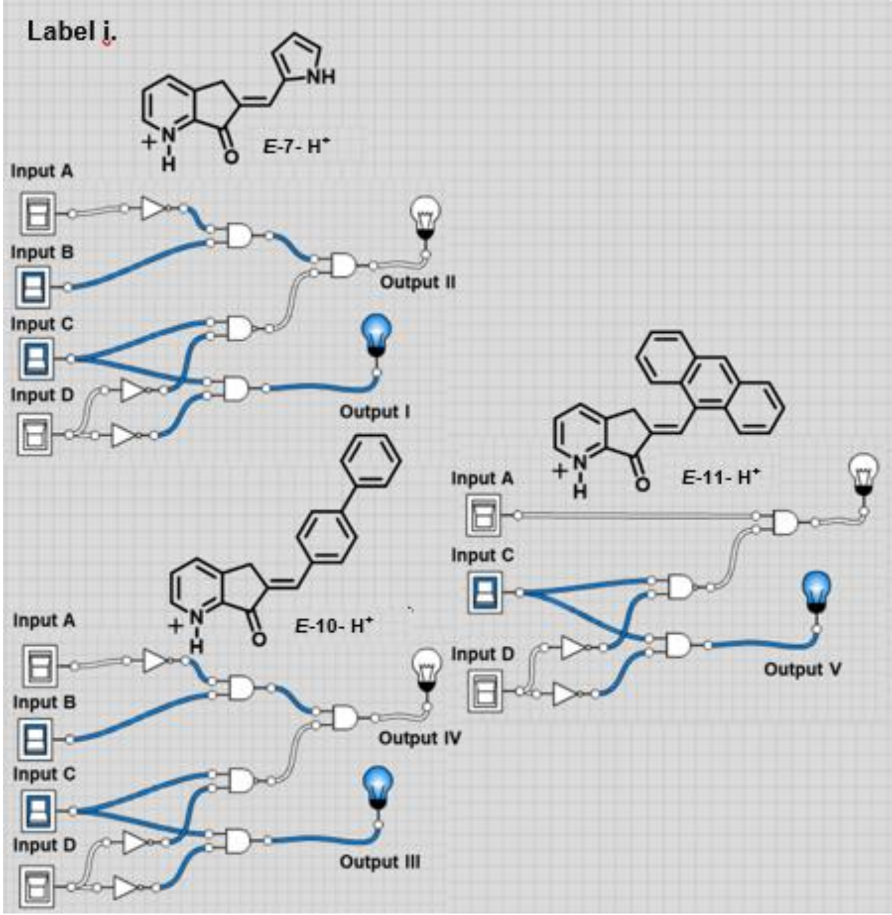
Label i.
E-7- H+
Input A
Input B
Input C
Input D
Output II
Output I
E-10- H+
Input A
Input B
Input C
Input D
Output IV
Output III
E-11- H+
Input A
Input C
Input D
Output V

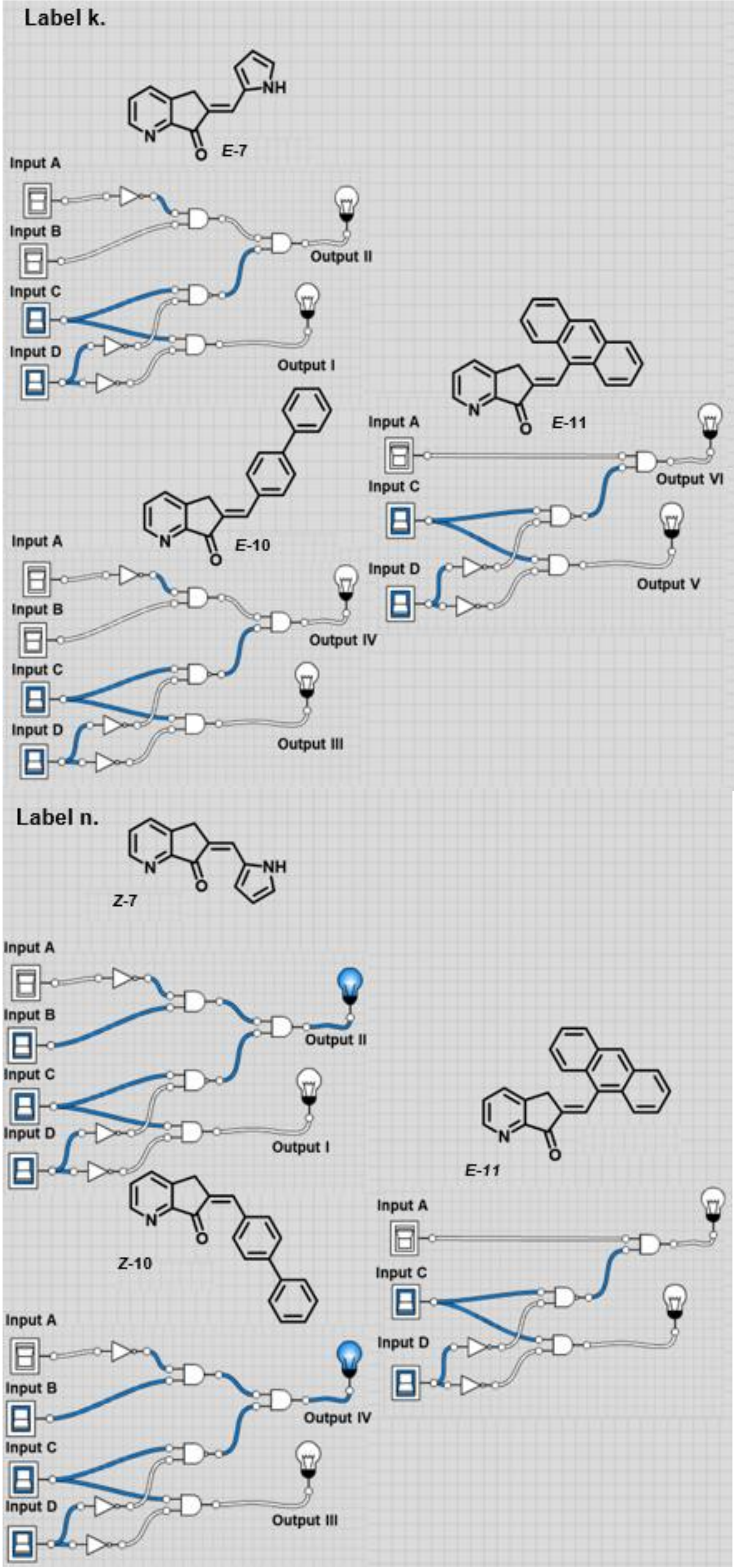
Label k.
E-7
Input A
Input B
Input C
Input D
Output II
Output I
E-11
Input A
Input C
Input D
Output VI
Output V
E-10
Input A
Input B
Input C
Input D
Output IV
Output III
Label n.
Z-7
Input A
Input B
Input C
Input D
Output II
Output I
E-11
Input A
Input C
Input D
Z-10
Input A
Input B
Input C
Input D
Output IV
Output III

Scheme S5. Diagrams show how the logical operation is solved with MLGs **7, 10** and **11** of each process carried out in the experiments with the theoretical labels. The concentrations of the two MLGs were 1.5 mM in $CDCl_3$:DMSO-d_6 (3:0.2 volume ratio), and NMR recorded each process at 500 MHz at room temperature.

Theory calculation of MLG 7

The geometry structure of the MLGs that was used for the theorical calculate, was the structure determinated with NMR results and corroboration with literature molecules; and then the MLG series was refined used DFT b3lyp method with basis set 6-31 + g (d, p).

Symbolic Z-matrix:

Charge = 0 Multiplicity = 1

C	-2.09232	3.41087	-4.33503
C	-1.29433	2.71497	-5.25969
C	-0.3024	1.84996	-4.7963
C	-0.15791	1.72399	-3.41542
C	-1.00783	2.46516	-2.58634
N	-1.96477	3.29965	-3.00973
H	0.32892	1.2994	-5.48943
H	-2.86792	4.08723	-4.68803
H	-1.45717	2.85693	-6.32373
C	-0.68208	2.18107	-1.15083
C	0.44861	1.19506	-1.17089
O	-1.22471	2.65425	-0.16522
C	0.80877	0.88101	-2.61172
H	1.84949	1.14397	-2.84571
H	0.68996	-0.18579	-2.84577
C	0.96285	0.74665	0.00003
H	0.56101	1.09701	0.92776
C	2.12358	-0.26543	-0.00002
C	2.80431	-0.85897	-1.07216

N	2.6713	-0.74306	1.18699
C	3.80026	-1.72763	-0.51326
H	2.61419	-0.69313	-2.12723
C	3.69462	-1.63556	0.88147
H	2.38231	-0.49134	2.09355
H	4.50323	-2.34069	-1.06716
H	4.26662	-2.13435	1.66284

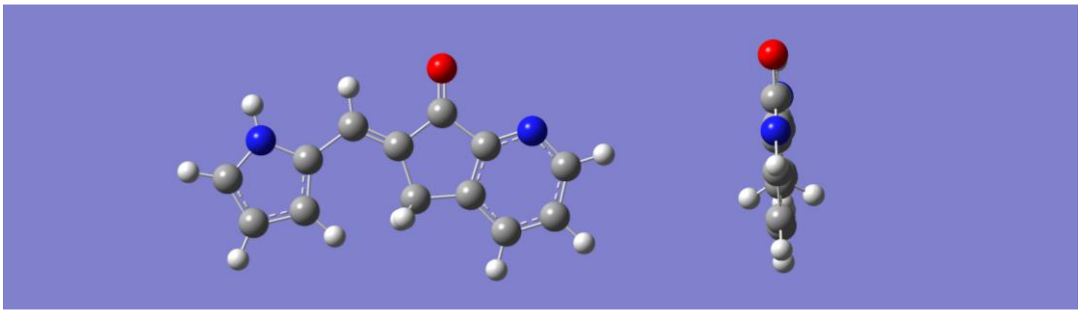

Figure S37. Structure of MLG E-7 refined with DFT.

The next part to obtain the theorical absorption spectrum of ***E*-7**, was tested methods and basis set to find the better match of the experimental results with theorical data. The screening was tested with 3 different DFT methods; b3lyp, b3pw91 and cam-b3lyp and the same basis set 3-21+g*, with b3lyp 3 different basis set 3-21+g*, 6-31+g(d), and Sto-3g. Accordingly with these data, the best method is the b3lyp with the 3-21+g* basis set and the solvation model iefpcm/CH_3Cl.

Table S5. Comparation data of DFT methods for ***E*-7** simulate UV/Vis absorption spectra

Entry	Method	Basis set	Solvatation	λ_{max} (nm)	f	ε≃ ($M^{-1}cm^{-1}$)	Energy (eV)
	Experimental	-	CH_3Cl	400.35	-	20,200	3.0969
1	b3lyp	3-21+g*	-	373.31	0.4288	17,000	3.3212
2	b3lyp	3-21+g*	iefpcm/CH_3Cl	399.74	0.5465	21,000	3.1016
3	b3lyp	6-31+g(d)	iefpcm/ CH_3Cl	408.57	0.5450	21,000	3.0346
4	b3lyp	Sto-3g*	iefpcm/ CH_3Cl	315.42	0.3793	15,000	3.9307
5	b3pw91	3-21+g*	iefpcm/ CH_3Cl	397.17	0.5474	21,000	3.1217
6	cam-b3lyp	3-21+g*	iefpcm/ CH_3Cl	340.81	0.6748	27,000	3.6379

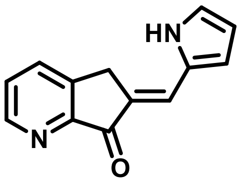

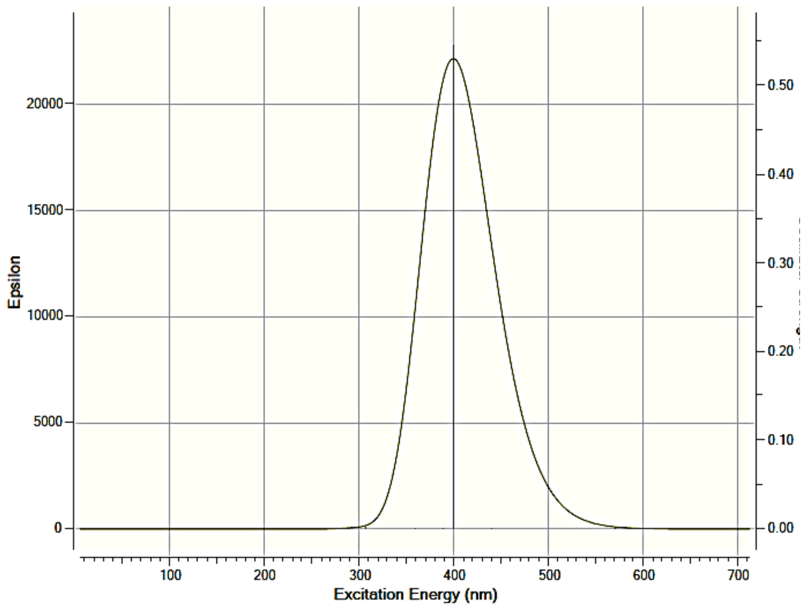

ε=21,000M^{-1}cm^{-1}

f=0.5465

λ_{max}=399.74nm

Figure S37. Theory UV/Vis absorption spectra, and data. Method b3lyp with the 3-21+g* basis set and the solvation model iefpcm/CH_3Cl.

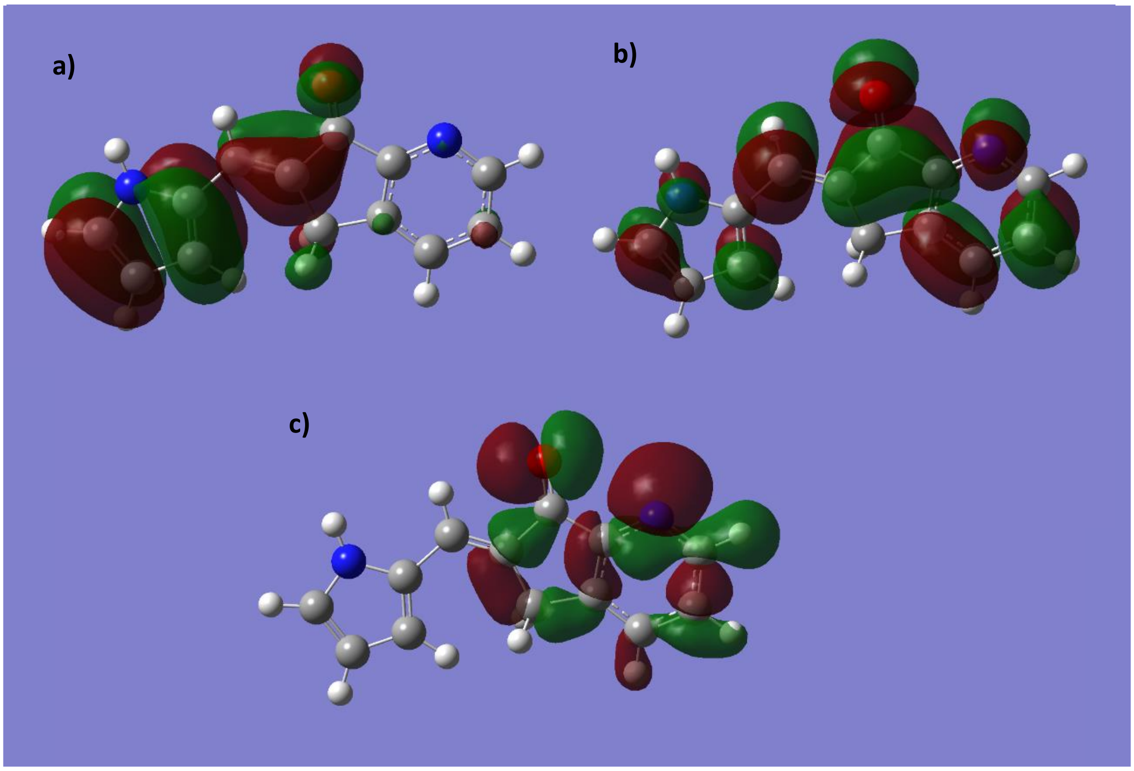

Figure S38. a) Orbital π that correspond of MLG ***E*-7** HOMO. b) Orbital π* that correspond of MLG ***E*-7** LUMO. c) MO that have n orbital of MLG ***E*-7**.
